# Supplementary material for: Analysis of Gene Differences Between F and B Epidemic Lineages of Bandavirus Dabieense
Source: Microorganisms. 2025 Jan 28;13(2):292. doi: 10.3390/microorganisms13020292 (PMC11857831; doi:10.3390/microorganisms13020292)
Supplement: Supplementary file 1 [file microorganisms-13-00292-s001.zip › Supplementary Table 1.pdf]

Supplementary Table 1: SFTSV Timeline Data Collection Table

**Country**	**Location**	**Time**	**Species**	**Quantity and detection**	**reference**
China	Shandong Province	2011	Cattle, sheep, dogs, pigs, chickens	Positive	[1]
China	Shandong Province	2011	Homo sapiens	Positive	
China	Shandong Province	2011	Haemaphysalis Iongicornis、Rhipicephalus sanguineus and Rhipicephalus microplus	Positive	
China	Shandong Province	2011	Homo sapiens	22	[2]
China	Jiangsu Province	2011	Homo sapiens	11	[3]
China	Henan Province	2011-2012	Homo sapiens	311	[4]
China	Shandong Province	2019	Hedgehog/Tick	Positive	[5]
China	Henan Province	2012	Homo sapiens	43	[6]
China	Liaoning Province	2013	Homo sapiens	1	[7]
Ch	Jiangsu Province	2011-	Homo sapiens	28	[8]

ina		2012			
China	Jiangsu Province	2010-2011	Rat	12	[9]
China	Hubei province	2012	Homo sapiens	33	[10]
China	Jiangsu Province	2010	Rat	12	[11]
China	Jiangsu Province	2011	Homo sapiens	1	[12]
China	Liaoning Province	2011	Homo sapiens	4	[13]
China	Jiangsu Province	2010	Sheep, Rat/Mite and Chigger Mite	60 Poisonous Spiny Mites and 100 Small Shield Fibers	[14]
China	Jiangsu Province	2010	Homo sapiens	33	[15]
China	Shandong Province	2010	Tick	Positive	[16]
China	Jiangsu Province	2010-2011	Chicken, cow	Positive	[17]
China	Henan Province	2004-2013	Homo sapiens	946	[18]
China	Henan Province	2011	Homo sapiens	33	[19]
Ch	Jiangsu Province	2011-	Homo sapiens	22	[20]

ina		2012			
China	Henan Province	2011	Homo sapiens	143	[21]
China	Zhejiang Province	2012	Homo sapiens	2	[22]
China	Jiangsu Province	2010	Homo sapiens	5	[23]
China	Jiangsu Province	2010	Goose, sheep, and chicken	Positive	
China	Shandong Province	2011	Homo sapiens	35	[24]
China	Shandong Province	2011	Cattle, sheep, dogs, pigs, chickens	642	
China	Hubei province	2006-2012	Homo sapiens	11	[25]
China	Jiangsu Province	2012	Sheep/Tick	Positive	[26]
China	Zhejiang Province	2012	Homo sapiens	1	[27]
China	Zhejiang Province	2011	Homo sapiens	3	[28]
China	Anhui Province	2010-2011	Homo sapiens	9	[29]
China	Jiangsu Province	2010-2011	Homo sapiens	3	
Ch	Zhejiang Province	2011	Homo sapiens	1	[30]

ina					
China	Anhui Province	2010-2013	Homo sapiens	3	[31]
China	Hubei province	2011-2012	Homo sapiens	Positive	[32]
China	Hubei province	2011-2012	Cattle, sheep, dogs	Positive	
China	Liaoning Province	2009-2011	Homo sapiens	53	[33]
China	Liaoning Province	2009—2011	Tick	20	
China	Liaoning Province	2012-2013	Homo sapiens	124	[34]
China	Liaoning Province	2012	Homo sapiens	38	[35]
China	Henan Province	2004-2014	Homo sapiens	946	[36]
China	Hubei province	2012	Homo sapiens	32	[37]
China	Hubei province	2009	Homo sapiens	85	[38]
China	Anhui Province	2011-2016	Homo sapiens	316	[39]
China	Henan Province	2011-2015	Homo sapiens	3373	[40]

China	Henan Province	2011-2014	Homo sapiens	2377	[41]
China	Shandong Province	2011-2013	Homo sapiens	95	[42]
China	Shandong Province	2011-2014	Homo sapiens	87	[43]
China	Henan Province	2007-2011	Homo sapiens	422	[44]
China	Liaoning Province	2010-2015	Homo sapiens	98	[45]
China	Shandong Province	2011-2015	Homo sapiens	90	[46]
China	Hubei province	2010-2013	Homo sapiens	101	[47]
China	Anhui Province	2013-2015	Homo sapiens	27	[48]
China	Shandong Province	2013-2015	Homo sapiens	1277	[49]
China	Shandong Province	2012	Homo sapiens	11	[50]
China	Jiangsu Province	2012	Rat、Cattle and sheep	49	[51]
China	Zhejiang Province	2012-2014	Homo sapiens	9	[52]
China	Henan Province	2012-2013	Homo sapiens	554	[53]

China	Zhejiang Province	2014	Homo sapiens	57	[54]
China	Anhui Province	2011-2017	Homo sapiens	87	[55]
China	Anhui Province	2011-2015	Homo sapiens	575	[56]
China	Liaoning Province	2010-2015	Homo sapiens	101	[57]
China	Liaoning Province	2011-2013	Homo sapiens	55	[58]
China	Liaoning Province	2011-2013	Homo sapiens	111	[59]
China	Hubei province	2013	Tick	8	[60]
China	Shandong Province	2014	Homo sapiens	21	[61]
China	Anhui Province	2015-2016	Homo sapiens	36	[62]
China	Hubei province	2011-2015	Homo sapiens	1458	[63]
China	Almost nationwide	2011-2016	Homo sapiens	5360	[64]
China	Hubei province	2012-2014	Homo sapiens	17	[65]
China	Hubei province	2013	Homo sapiens	18	[66]

China	Shandong Province	2015	Homo sapiens	2	[67]
China	Zhejiang Province	2011-2013	Homo sapiens	27	[68]
China	Zhejiang Province	2015	Homo sapiens	2	[69]
China	Shandong Province	2013-2017	Homo sapiens	186	[70]
China	Henan Province	2014	Tick	36	[71]
China	Shandong Province	2014	Homo sapiens	16	[72]
China	Anhui Province	2014	Homo sapiens	2	[73]
China	Anhui Province	2011-2015	Homo sapiens	186	[74]
China	Anhui Province	2013-2015	Homo sapiens	30	[75]
China	Anhui Province	2013-2016	Homo sapiens	14	[76]
China	Anhui Province	2013-2016	Rat	97	
China	Shandong Province	2014-2015	Homo sapiens	74	[77]
China	Shandong Province	2013	Homo sapiens	154	[78]

China	Shandong Province	2013-2016	Homo sapiens	17	[79]
China	Zhejiang Province	2017	Homo sapiens	1	[80]
China	Zhejiang Province	2013	Homo sapiens	1	[81]
China	Hubei province	2010-2014	Homo sapiens	13	[82]
China	Shandong Province	2014	Tick	126	[83]
China	Shandong Province	2015-2016	Homo sapiens	30	[84]
China	Hubei province	2014	Homo sapiens	2	[85]
China	Zhejiang Province	2013	Homo sapiens	1	[86]
China	Xinjiang Uyghur Autonomous Region	2014	Tick/Rat	Positive	[87]
China	Zhejiang Province	2013-2015	Tick	Positive	[88]
China	Anhui Province	2014	Homo sapiens	2	[89]
China	Shandong Province	2013-2019	Homo sapiens	370	[90]
China	Zhejiang Province	2012-2018	Homo sapiens	44	[91]

China	Shandong Province	2012-2020	Homo sapiens	253	[92]
China	Anhui Province	2011-2022	Homo sapiens	4298	[93]
China	Shandong Province	2011-2020	Homo sapiens	4642	[94]
China	Shandong Province	2011-2018	Homo sapiens	400	[95]
China	Hubei province	2010-2017	Homo sapiens	1210	[96]
China	Jiangsu Province	2010-2018	Homo sapiens	117	[97]
China	Anhui Province	2016-2019	Homo sapiens	95	[98]
China	Henan Province	2017-2018	Homo sapiens	74	[99]
China	Liaoning Province	2011-2018	Homo sapiens	54	[100]
China	Jiangsu Province	2017	Homo sapiens	1	[101]
China	Zhejiang Province	2015-2019	Homo sapiens	337	[102]
China	Liaoning Province	2015-2018	Homo sapiens	117	[103]
China	Shandong Province	2014-2019	Homo sapiens	102	[104]

China	Shandong Province	2014-2017	Homo sapiens	191	[105]
China	Anhui Province	2013-2022	Homo sapiens	286	[106]
China	Shandong Province	2013-2018	Homo sapiens	385	[107]
China	Zhejiang Province	2021	Homo sapiens	37	[108]
China	Beijing	2021	Homo sapiens	1	[109]
China	Almost nationwide (newly added in Shanghai)	2018	Homo sapiens	1848	[110]
China	Shandong Province	2018-2021	Homo sapiens	610	[111]
China	Henan Province	2018-2021	Homo sapiens	1461	[112]
China	Henan Province	2018	Homo sapiens	1	[113]
China	Henan Province	2018-2020	Homo sapiens	992	[114]
China	Jilin Province	2017	Tick	Positive	[115]
China	Zhejiang Province	2021	Homo sapiens	85	[116]
China	Zhejiang Province	2012-2020	Homo sapiens	65	[117]

China	Zhejiang Province	2017	Homo sapiens	1	[118]
China	Zhejiang Province	2013-2017	Homo sapiens	29	[119]
China	Anhui Province	2020	Homo sapiens	24	[120]
China	Zhejiang Province（The first case in Lanxi City）	2020	Homo sapiens	1	[121]
China	Hubei province	2014-2018	Homo sapiens	34	[122]
China	Liaoning Province	2016-2019	Homo sapiens	17	[123]
China	Liaoning Province	2013-2018	Homo sapiens	40	[124]
China	Anhui Province	2018-2022	Homo sapiens	152	[125]
China	Shandong Province	2010-2019	Homo sapiens	462	[126]
China	Shandong Province	2016-2020	Homo sapiens	137	[127]
China	Jiangsu Province	2010-2016	Homo sapiens	99	[128]
China	The first case in Beijing	2021	Homo sapiens	1	[129]
China	Anhui Province	2011-2016	Homo sapiens	137	[130]

China	Jiangsu Province	2016	Homo sapiens	207	[131]
China	Zhejiang Province	2014-2016	Homo sapiens	7	[132]
China	Shandong Province	2010-2016	Homo sapiens	2319	[133]
China	Henan Province	2010-2019	Homo sapiens	377	[134]
China	Zhejiang Province	2015-2017	Homo sapiens	41	[135]
China	Liaoning Province	2011-2017	Homo sapiens	438	[136]
China	Jilin Province	2016	Tick	26	[137]
China	Zhejiang Province	2011-2018	Homo sapiens	140	[138]
China	Shandong Province	2016-2018	Homo sapiens	100	[139]
China	Henan Province	2022	Homo sapiens	6	[140]
China	Guangdong Province	2017	Homo sapiens	1	[141]
China	Jiangsu Province	2015-2020	Homo sapiens	194	[142]
Korea	Almost nationwide	2015-2017	Deer	Positive	[143]

China	Hubei province	2010-2016	Sheep, cattle, dogs, rodents	544	[144]
China	Anhui Province	2022-2023	Tick	3	[145]
China	Zhejiang Province	2022-2023	Tick	36	
China	Henan Province	2022-2023	Tick	58	
China	Guangdong Province	2021	Homo sapiens	1	[146]
China	Zhejiang Province	2021	Homo sapiens	85	[116]
China	Zhejiang Province	2020-2022	Homo sapiens	9	[147]
China	Henan Province	2017-2020	Homo sapiens	767	[148]
Korea	Gyeongbuk	2018	Haemaphysalis Iongicornis, Haemaphysalis birmaniae, Haemaphysalis mageshimaensis	Positive	[149]
Japan	Nagasaki	2015	Homo sapiens	2	[150]
Japan	Ehime Prefecture	As of 2017	Homo sapiens	297	[151]
Japa	Ehime Prefecture	As of 2017	Animal	Positive	

n					
China	Liaoning Province、Jilin Province and Heilongjiang Province	2013	Haemaphysalis Iongicornis、Dermacentor silvarum、Dermacentor nuttalli	Positive	[152]
Korea	-	2017	Deer	3	[153]
Korea	-	2017	Goat	4	[154]
China	Zhejiang Province	2018	Homo sapiens	13	[155]
Korea	-	2015	Homo sapiens	22	[156]
U.S.A	Minnesota State	2014	Animal	Positive	[157]
Japan	-	1980-2000	Animal	negative	[158]
Korea	-	2013	Haemaphysalis Iongicornis	Positive	[159]
China	Shandong Province	2014-2016	Hedgehog	Positive	[160]
China	Shandong Province	2011	Sheep	38	[161]
China	Jiangsu Province	2012-2013	Goat, cow, dog, pig, chicken, goose, rodent, hedgehog	Positive	[162]

China	Jiangsu Province	2012-2013	Homo sapiens	33	
China	Zhejiang Province	2013	Homo sapiens	71	[163]
Japan	-	2019	Semen	Positive	[164]
Vietnam	Hue	2017	Homo sapiens	80	[165]
China	Henan Province	2011-2017	Homo sapiens	2096	[166]
China	Zhejiang Province	2011-2013	Homo sapiens	65	[167]
China	Taizhou, Zhejiang Province	2020-2021	Animal、Tick	Positive	[168]
China	Zhejiang Province	2020-2021	Homo sapiens	Positive	
Japan	Okinawa Island	2002-2018	Mongoose	Positive	[169]
Japan	-	2013-2017	Homo sapiens	303	[170]
Japa	-	2013	Homo sapiens	96	[171]

n					
China	Shandong Province	2010-2017	Homo sapiens	2731	[172]
Korea	-	2022	Catamount	4	[173, 174]
China	Anhui Province	2015-2021	Homo sapiens	6500	[175]
泰国	-	2019-2020	Homo sapiens	3	[176]
Korea	-	2016-2018	Tick	Positive	[177]
China	Jiangsu Province	2011-2014	Erinaceus europaeus、Sorex araneus<br>migratory birds Anser cygnoides	Positive	[178]
China	Henan Province	2016	Homo sapiens	12	[179]
China	Henan Province	2016-2018	Cattle, sheep, dogs, pigs, chickens, ducks, yellow rats, wolves, wild rabbits、Rat、Hedgehog、Wild boar, badger, rock pigeon, rat, pheasant, spotted dove	374	[180]
Korea	-	2013-2019	Homo sapiens	1086	[181]
Ko	-	2019-	Tick	19	[182]

rea		2020			
Korea	-	2013-2015	Homo sapiens	173	[183]
China	Henan Province	2015	Tick	Positive	[184]
China	Taiwan, China	2019	Homo sapiens	1	[185]
China	Anhui Province	2015	Homo sapiens	257	[186]
Korea	-	2019	Tick	Positive	[187]
China	Shandong Province	2014-2018	Homo sapiens	5670	[188]
Korea	-	2019-2020	Dog	Positive	[189]
Japan	-	2013-2020	Homo sapiens	498	[190]
China	Henan Province、Shandong Province、Hubei province、Anhui Province1、Jiangsu Province、Zhejiang Province、Liaoning Province	2011-2021	Homo sapiens	18902	[191]
China	Shandong Province	2015	Mink	Positive	[192]

Korea	-	2020	Tick	Positive	[193]
China	Zhejiang Province	2011-2019	Homo sapiens	463	[194]

reference

[1] Guoyu N. The epidemiological investigation of potential vectors and hosts of SFTS virus in China.Chinese Center For Disease Control And Prevention, 2013.

[2] Yuanyuan C. Preliminary Study on Epidemilogical Characteristies ofSevere Fever with Thrombocytopenia Syndrome in Yiyuan Country, Shandong Province. Shandong University, 2013.

[3] Liang S. The Investigation on Severe Fever with Thrombocytopenia(SFTS) Virus Infection and the Potential Vector of SFTS Virus. Nanjing Medical University, 2014.

[4] Wang L. Clinical Characteristic in Patients with Severe Fever with Thrombocytopenia Syndrome. Anhui Medical University, 2014.

[5] Geng H,Fachun J,Liyan D, et al. Identification of predominant microbial populations carried by five species of ticks in Qingdao, Shandong. Disease Surveillance, 2023, 38(03):264-269.

[6] Ning C, Zeng L De, Wen G, et al. Study on the clinical characteristics of severe fever with thrombocytopenia syndrome bunyavirus patients. Journal of Logistics University of PAP(Medical Sciences), 2013, 22(04):256-259.

[7] Ying X. Toxic epidermal necrolysis complicated by novel SFTS bunyavirus infection： a case report and literature review. Dalian Medical University, 2014.

[8] Yan L, Ke J, Yaping H, et al. Dynamic Changes of Nucleoprotein-specific Antibodies in the Serum of Patients with Sever Fever With Thrombocytopenia Syndrome. Progress in Modern Biomedicine, 2013, 13(18):3432-3435.

[9] Qingkui W, Zhifeng L, Hu Jianli, et al. Surveillance of vectors and host animals of severe fever with thrombocytopenia syndrome virus in Donghai, China in 2010-2011. Chinese Journal of Vector Biology and Control, 2013, 24(04):313-316.

[10] Yanfang L . The Preliminary Study on the Cellular mmune Funetion ofFever with Thrombocytopenia Syndrome Patients in the Acute Phase and the Recovery

Phase. Huazhong University of Science and Technolog, 2013.
[11] Qingkui W, Hengming G, Jianli H, et al. Survey of infection of severe fever with thrombocytopenia syndrome virus in mice. Modern Preventive Medicine, 2013, 40(15):2902-2904.
[12] Jian C, Hongxia W, Yun C, et al. One severe fever case with thrombocytopenia syndrome and invasive pulmonary aspergillosis and review of the literatures. Chinese Journal of Experimental and Clinical Infectious Diseases, 2013, 7(03):118-121.
[13] Yun J, Jie Z, Bo W, et al. Detection and analysis on severe fever with thrombocytopenia svndrome bunyavirus in blood from a patient bitted by tick in Dalian City. Chinese Journal of Zoonoses, 2013, 29(02):159-161.
[14] Qingkui W, Ge Hengming G, Zhifeng L, et al. Vector research of severe fever with thrombocytopenia syndrome virus in gamasid mites and chigger mites. Chinese Journal of Vector Biology and Control, 2012, 23(05):452-454.
[15] Jun D, Zhen T, Lunbiao C, et al. Detection of RNA and analysis of complete genome of severe fever with thrombocytopenia syndrome virus （SFTSV） in Jiangsu. Modern Preventive Medicine, 2013, 40(14):2686-2689+2696.
[16] Xiaolin J, Xianjun W, Jiandong L, et al. Isolation,Identification and Characterization of SFTS Bunyavirus from Ticks Collected on the Surface of Domestic Animals. Chinese Journal of Virology, 2012, 28(03):252-257.
[17] Yanping W, Qingkui W, Hengming G, et al. Serological surveillance of SFTSV infection. Jiangsu Journal of Preventive Medicine, 2013, 25(02):26-28.
[18] Wenqing F, Zhihua L, Shengwang P, et al. Analysis of clinical and epidemiological characteristics of severe fever with thrombocytopenia syndrome in high affected area. Journal of Luzhou Medical College, 2013, 36(06):591-596.
[19] Ling W, Haixia C, Feng C, et al. Surveillance and analysis of severe fever with thrombocytopenia syndrome in Zibo City. Modern Preventive Medicine, 2013, 40(18):3471-3474.
[20] Yaping H, Jun L, Yongxiang Z, et al. Role of T lymphocyte activation in the development of severe fever accompanied by thrombocytopenia syndrome. Chinese Journal of Cellular and Molecular Immunology, 2013, 29(06):637-640+643.
[21] Yanhua D, Xueyong H, Haifeng W, et al. Comparison on realtime PCR and ELISA methods in detection of severe fever with thrombocvtopenia syndrome (SFlS) cases. Chinese Journal of Zoonoses, 2013, 29(01):101-104.
[22] Xiang L, Chensong C, Tingting K, et al. Clinical and epidemiological characteristics of 2 cases of thrombocytopenia syndrome accompanied by fever in Ningbo. Disease Surveillance, 2013, 28(01):13-16.
[23] Z G ,S G ,Y Z , et al. Probable aerosol transmission of severe fever with thrombocytopenia syndrome virus in southeastern China. Clinical Microbiology and Infection, 2015, 21(12):1115-20.

[24] Zongdong L, Haiying Y, Yufang J, et al. Survey on Antibody of New Bunyavirus Amoug Healthy People and Domestic Animals. Preventive Medicine Tribune, 2014, 20(04):274-276.
[25] Yuping Z, Chuanxin Z, Taozhen C, et al. Clinical analysis on 1l cases of fever with thrombocytopenia syndrome. Journal of Clinical Hematology, 2013, 26(06):411-412.
[26] Chengqiang H , Naizheng D, et al. Discovery of Severe Fever with Thrombocytopenia Syndrome Bunyavirus Strains Originating from Intragenic Recombination. Journal of Virology, 2012, 86(22): 12426-30.
[27] Xuejuan W, Guangjun P, Li G, et al. Epidemiological investigation of a case of fever with thrombocytopenia syndrome. Shanghai Journal of Preventive Medicine, 2012, 24(12):662-663.
[28] Xueyong H , Licheng L , Yanhua D, et al. The Evolutionary History and Spatiotemporal Dynamics of the Fever, Thrombocytopenia and Leukocytopenia Syndrome Virus (FTLSV) in China. PLOS Neglected Tropical Diseases, 2014, 8(10):e3237.
[29] Shoujun Z, Yulan H, Tingting G, et al. Clinical Analysis of 12 Cases of Fever with Thrombocytopenia Syndrome Caused by Novel Bunyavirus. Jiangsu Journal of Preventive Medicine, 2012, 38(05):583-584.
[30] Yun S, Park S, Park S, et al. Molecular genomic characterization of tick- and human-derived severe fever with thrombocytopenia syndrome virus isolates from South Korea. PLOS Neglected Tropical Diseases, 2017, 11(9):e0005893.
[31] Zhiyong S, Jian L, Youliang S, et al. Suspected severe fever with thrombocytopenia syndrome in Tongling area: Clinical features in 24 cases. Journal of Wannan Medical College, 2014, 33(01):60-62.
[32] Weihua H, Fang G, Zuyin L,, et al. Study on the epidemiological characteristics of fever accompanying by thrombocytopenia syndrome in Suizhou. Chinese Journal of Health Laboratory Technology, 2014, 24(01):117-119.
[33] Yun L, Jie Z, Bo W,, et al. Epidemic characteristics and biological features of severe fever with thrombocytopenia syndrome bunyavirus (SFTSV) found in Liaoning province. Chinese Journal of Public Health, 2013, 29(05):721-723.
[34] Tani H, Kawachi K, Kimura M, et al. Identification of the amino acid residue important for fusion of severe fever with thrombocytopenia syndrome virus glycoprotein. Virology, 2019, 535: 102-10.
[35] Noda K, Tsuda Y, Kozawa F, et al. The Polarity of an Amino Acid at Position 1891 of Severe Fever with Thrombocytopenia Syndrome Virus L Protein Is Critical for the Polymerase Activity. Viruses, 2020, 13(1):33.
[36] Choi Y, Park Sj, Sun Y, et al. Severe fever with thrombocytopenia syndrome phlebovirus non-structural protein activates TPL2 signalling pathway for viral immunopathogenesis. Nature Microbiology, 2019, 4(3): 429-37.

[37] Sun L ,Li H ,Yi X , et al.Detection and Clinical Significance of Circulating Microvesicles in Severe Fever with Thrombocytopenia Syndrome. Infection International, 2013, 2(02):49-54.

[38] Lucy G . The Impacts of Climate Change on Ticks and Tick-Borne Disease Risk. Annual Review of Entomology, 2021, 66(1): 373-88.

[39] Ogden Nh, Lindsay Lr, et al. Effects of Climate and Climate Change on Vectors and Vector-Borne Diseases: Ticks Are Different. Trends in Parasitology, 2016, 32(8): 646-56.

[40] Miao D, Liu Mj, Wang Yx, et al. Epidemiology and Ecology of Severe Fever With Thrombocytopenia Syndrome in China, 2010–2018. Clinical Infectious Diseases, 2021, 73(11): e3851-e8.

[41] Aiguo Y, Jianhua Y, Yan H, et al. Analysis of epidemical characteristic of severe fever with thrombocytopenia syndrome in Xinyang, 2011-2014. Modern Preventive Medicine, 2015, 42(17):3082-3084+3096.

[42] Wormser Gp, Mckenna D, Piedmonte N, et al. First Recognized Human Bite in the United States by the Asian Longhorned Tick, Haemaphysalis longicornis. Clinical Infectious Diseases, 2020, 70(2): 314-6.

[43] Yun Y, Heo St, Kim G, et al. Phylogenetic Analysis of Severe Fever with Thrombocytopenia Syndrome Virus in South Korea and Migratory Bird Routes Between China, South Korea, and Japan. The American Journal of Tropical Medicine and Hygiene, 2015, 93(3): 468-74.

[44] Zhe X, Ning C, Weiwei C,, et al. Clinical characteristics in 422 patients with severe fever with thrombocytopenia syndrome. Infectious Disease Information, 2015, 28(01):28-32.

[45] Qiang L, Guijing S, Baocheng D, et al. Risk factor for fatality in 98 patients with severe fever with thrombocytopenia syndrome. Medical Journal of Liaoning, 2017, 31(06):35-37.

[46] Barrett B , Charles W J , Temte L J, et al. Climate change, human health, and epidemiological transition. Preventive Medicine, 2015, 70: 69-75.

[47] Fang G, Weihua H, Xiaohui L, et al. Surveillance for severe fever with thrombocytopenia syndrome in Suizhou,Hubei,2010 － 2013. Disease Surveillance, 2014, 29(10):806-809.

[48] Hofmann H, Li X, Zhang X, , et al. Severe Fever with Thrombocytopenia Virus Glycoproteins Are Targeted by Neutralizing Antibodies and Can Use DC-SIGN as a Receptor for pH-Dependent Entry into Human and Animal Cell Lines. Journal of Virology, 2013, 87(8): 4384-94.

[49] Bo P, Shujun D, Xiaomei Z, et al. Analysis on epidemiological features ot severe tever with bocytopenia syndrome. Preventive Medicine Tribune, 2017, 23(02):81-84.

[50] Yuan F, Zheng A Yuan F ,Zheng A , et al. Entry of severe fever with thrombocytopenia syndrome virus. Virologica Sinica, 2016, 32(1): 44-50.

[51] Haiyan P, Cui L, Lunbiao C, et al. Viruscarrying Status of Animals with Fever and Thrombocytopenia Syndrome in Some Regions of Jiangsu Province during

2012. Acta Medicinae Universitatis Scientiae et Technologiae Huazhong, 2015, 44(02):235-238.
[52] Liu T, Li J, Liu Y, et al. SNX11 Identified as an Essential Host Factor for SFTS Virus Infection by CRISPR Knockout Screening. Virologica Sinica, 2019, 34(05): 508-520.
[53] Drake Mj, Brennan B, Briley K Jr, et al. A role for glycolipid biosynthesis in severe fever with thrombocytopenia syndrome virus entry. Public Library of Science Pathogens, 2017, 13(4):e1006316.
[54] Yamaoka S, Weisend C, Ebihara H, et al. Identifying target cells for a tick-borne virus that causes fatal hemorrhagic fever. Journal of Clinical Investigation, 2020, 130(2): 598-600.
[55] Ting W, Yan L, Hongru L, et al. Clinical features and prognostic risk factors of novel Bunyavirus infection in Chaohu Area. Chinese Journal of Disease Control and Prevention, 2017, 21(01):56-60.
[56] Chen C, Jia W Bing, Minghua C, et al. Epidemiological analysis on severe fever with thrombocytopenia syndrome in Anhui Province,2011 — 2015. Chinese Journal of Disease Control and Prevention, 2016, 20(08):792-795.
[57] Rongmin C, Danmei Y, Changqing M, et al. Analysis of the epidemic characteristics of severe fever with thrombocytopenia syndrome in Dandong city of Liaoning province, 2010-2015. Chinese Journal of Vector Biology and Control, 2017, 28(01):60-63.
[58] Liang Yuhong L, Shang Q, Ling Z, et al. Epidemic of severe fever with thrombocytopenia syndrome in Dalian city, 2011—2013. Chinese Journal of Public Health, 2015, 31(03):373-374.
[59] Lei Y, Peng Y, Bing, X, et al. Fever with thrombocytopenia syndrome bunya virus Surveillance analysis in Dalian during 2011 — 2013. Journal of Medical Pest Control, 2015, 31(04):422-424+427.
[60] Suzuki T, Sato Y, Sano K, et al. Severe fever with thrombocytopenia syndrome virus targets B cells in lethal human infections. Journal of Clinical Investigation, 2020, 130(2): 799-812.
[61] Matsuno K, Orba Y, Maede White K, et al. Animal Models of Emerging Tick-Borne Phleboviruses: Determining Target Cells in a Lethal Model of SFTSV Infection. Frontiers in Microbiology, 2017, 8104.
[62] Kun J, Xiaoling W, Qiang S, et al. Clinical analysis of 36 cases of severe fever with thrombocytopenia syndrome. Anhui Medical and Pharmaceutical Journal, 2018, 22(07):1342-1345.
[63] Xuesen X. Study on epidemiological characteristies and transmission for Severe Fever With Thrombocytopenia Syndrome. Huazhong University of Science and Technolog, 2016.
[64] Jimin S. Study on spatiotemporal prediction of fever with thrombocytopenia syndrome. Chinese Center for Disease Control and Prevention, 2018.

[65] Bo X. Study on the Epidemiological and Clinical features of Severe fever with thrombocytopenia syndrome, Academy of Military Sciences. Academy of Military Sciences, 2018.
[66] Xinjun L, Jinping K, Xin Z, et al. Epidemiological characteristics and epidemic analysis of 18 cases of fever with thrombocytopenia syndrome. Journal of Hubei University of Science and Technology, 2014, 28(03):256-258.
[67] Ping X, Hanwei K. Analysis of 2 cases of severe novel Bunyavirus infection. The Medical Forum, 2017, 21(04):504-505.
[68] Huang M, Wang T, Huang Y, et al. The clinical and immunological characteristics in fatal severe fever with thrombocytopenia syndrome virus (SFTSV) infection. Clinical Immunology, 2023, 248109262-109262.
[69] Yaxin D, Kefeng L, Peng L, et al. Epidemiological investigation of a family cluster of two patients with severe fever with thrombocytopenia syndrome in Gouqi island of Zhoushan city, Zhejiang province. Chinese Journal of Vector Biology and Control, 2018, 29(02):181-183.
[70] Li H, Lu Q, Xing B Li H ,Lu Q ,Xing B , et al. Epidemiological and clinical features of laboratory-diagnosed severe fever with thrombocytopenia syndrome in China, 2011–17: a prospective observational study. The Lancet Infectious Diseases, 2018, 18(10):1127-1137.
[71] Wang M, Huang P, Liu W, et al. Risk factors of severe fever with thrombocytopenia syndrome combined with central neurological complications: A five-year retrospective case–control study. Frontiers in Microbiology, 2022, 131033946-1033946.
[72] Tao L, Qiyan L, Mei J, et al. Survey on seroepidemiology and infection risk factors of severe fever with thrombocytopenia syndrome virus in Zhaoyuan healthy crowd. Modern Preventive Medicine 2018, 2018, 45(14):2654-2657+2679.
[73] Zhao C ,Liu J ,Li B, et al. Multiscale Construction of Bifunctional Electrocatalysts for Long-Lifespan Rechargeable Zinc–Air Batteries. Advanced Functional Materials, 2020, 30(36):n/a-n/a.
[74] Meichen H. Epidemiological characteristics of severe fever with thrombocytopenia syndrome in Zibo. Qingdao University, 2018.
[75] Ji L, Yong L, Min L, et al. Epidemiological and clinical characteristies of severe fever with thrombocytopenia syndrome. Chinese Preventive Medicine, 2017, 18(03):219-222.
[76] Pengpeng X, Yong L, Xingyi G, et al. Seroepidemiology of severe fever with thrombocytopenia syndrome virus, Liu'an. Modern Preventive Medicine, 2015, 42(11):1948-1950.
[77] Luyan S, Xiuzhen Z, Kuihua L, et al. Epidemiological and clinical analysis of 74 cases of severe fever with thrombocytopenia syndrome in Shandong Province, China from 2014 to 2015. Journal of Shandong University(Health Sciences), 2018, 56(12):98-102+117.
[78] Xiaolin J, Shujun D, Bo P, et al. Clinical and epidemiological characteristies of laboratory confirmed cases of severe fever with thrombocvtopenia syndrome infection in Shandong Province, China. CHINESE Journal of Zoonoses, 2017, 33(12):1077-1081.

[79] Tao L, Mei J, Xiaowen X, et al. Analysis on an outbreak of severe fever with thrombocytopenia syndrome from 2013 to 2016 in Yantai, Shandong. Chinese Journal of Vector Biology and Control, 2018, 29(05):511-513.

[80] Ye L, Yuqing L, Fuming L, et al. Investigation on the first case of novel Bunyavirus infection causing fever with thrombocytopenia syndrome in Lishui City. China Preventive Medicine Journal, 2018, 30(10):1031-1033+1036.

[81] Wenzhong H, Xianghua Y, Hongzhao W, et al. Investigation and analysis on the transmission of severe fever with thrombocytopenia syndrome via contact with patient. Chinese Journal of Vector Biology and Control, 2015, 26(02):172-175.

[82] Zhang Y, Bai X, Li J, et al. A CRISPR-based nucleic acid detection method for severe fever with thrombocytopenia syndrome virus. Virus Research, 2022, 311:198691.

[83] Yufang X, Jingyu L, Guoyu N, et al. Epidemiological investigation on the infection status of novel Buni virus among dominant tick species in rural hilly areas of Yantai City. Industrial Competitiveness and Innovation Driven: Proceedings of the 2014 Shandong Association for Science and Technology Annual Conference, 2014.

[84] Qiang L. Molecularepidemiology And Biological Characteristics Of Severe Fever With Thrombocytopenia Syndrome Virus In Taian. Shandong First Medical University, 2014.

[85] Kwangmin Y ,Sujin P ,Minah Y , et al. Cross-genotype protection of live-attenuated vaccine candidate for severe fever with thrombocytopenia syndrome virus in a ferret model. Proceedings of the National Academy of Sciences, 2019, 116(52):26900-26908.

[86] Fangfang D ,Dandan L ,Dan W , et al. Single dose of a rVSV-based vaccine elicits complete protection against severe fever with thrombocytopenia syndrome virus. NPJ vaccines, 2019, 4(1):5.

[87] Gary N E ,Weiner B D . DNA vaccines: prime time is now. Current Opinion in Immunology, 2020, 65: 21-27.

[88] Jungu K ,Kyeongseok J ,Hooncheol C , et al. Vaccination with single plasmid DNA encoding IL-12 and antigens of severe fever with thrombocytopenia syndrome virus elicits complete protection in IFNAR knockout mice. PLOS Neglected Tropical Diseases, 2020, 14(3):e0007813.

[89] Zhouxiang C, Fei W, Bangqun Q, et al. Investigation and disposal on the first cluster outbreak of person to person transmission of severe fever with thrombocytopenia syndrome in southern Anhui Province. Chinese Journal of Disease Control and Prevention, 2014, 18(11):1055-1058.

[90] Hong Z, Ying H, Yan L, et al. Monitoring on fever and bleeding syndrome,Jinan city,2013-2019. China Preventive Medicine Journal, 2020, 26(05):328-330+333.

[91] Weilong P, Ying L, Junhua G, et al. Epidemiological characteristics analysis on severe fever with thrombocytopenia syndrome in Tiantai County of Zhejiang Province,2012 — 2018. Shanghai Journal of Preventive Medicine, 2019, 31(06):500-504.

[92] Fangxiu X, Fachun J, Jianwen H, et al. Analysis on epidemiological characteristics of severe fever with thrombocytopenia syndromer,Qingdao city,2012-2020. Preventive Medicine Tribune, 2022, 28(05):368-370+381.
[93] Jia N . Large-Scale Comparative Analyses of Tick Genomes Elucidate Their Genetic Diversity and Vector Capacities. Sichuan Journal of Physiological Sciences, 2020, 182(5): 1328-40.
[94] Qing D, Bo P, Xiaomei Z, et al. Epidemiological characteristics and spatial aggregation of severe fever with thrombocytopenia syndrome in Shandong Province from 2011 to 2020. Chinese Journal of Disease Control and Prevention, 2022, 26(07):790-797.
[95] Weiru W, Xingyi G, Dong X, et al. The hot spot and epidemiological characteristics of severe fever with thrombocytopenia syndrome in ,inan in 2011-2018. Chinese Preventive Medicine, 2020, 21(04):458-463.
[96] Bennan D, Fen W. Analysis of epidemiological characteristics on cases of severe fever with thrombocytopenia syndrome in Huanggang(2010 —2017). Journal of Public Health and Preventive Medicine, 2019, 30(02):57-60.
[97] Tingting T, Zhiye X, Sen W, et al. Clinical characteristics analysis of 117 patients with fever accompanied by thrombocytopenia syndrome. Chinese Journal of Clinical Laboratory Science, 2020, 38(04):313-316.
[98] Ming Y, Zonghao Z, Yun Y, et al. Analysis of Risk Factors for Death in 95 Patients with Fever and Thrombocytopenia Syndrome. Journal of Anhui Medical College, 2020, 19(04):21-23+25.
[99] Hao L ,Xiaming J ,Ning C , et al. Clinical effect and antiviral mechanism of T-705 in treating severe fever with thrombocytopenia syndrome. Signal Transduction and Targeted Therapy, 2021, 6(1):145-145.
[100] Shang Q, Jun X, Yuhong L, et al. Epidemiological analysis of 54 death cases of severe fever with thrombocytopenia syndrome in Dalian City. Chinese Journal of Hygienic Insecticides & Equipments, 2019, 25(06):579-580.
[101] Wei L, Guoqing C, Dan X, et al. Analysis of the molecular characteristics of the whole genome of a human infection with new bunyavirus in Yancheng city in 2017. Jiangsu Journal of Preventive Medicine, 2021, 32(01):5-7+12.
[102] Xuguang S, Jimin S, Ying L, et al. Epidemiological characteristics of fever with thrombocytopenia syndrome in Zhejiang, 2015–2019. Disease Surveillance, 2021, 36(05):431-435.
[103] Shang Q, Yugong L, Wei W, et al. Epidemiological characteristics of severe fever with thrombocytopenia syndrome in Dalian City from 2015 to 2018. Chinese Journal of Hygienic Insecticides & Equipments, 2019, 25(05):471-473.
[104] Masanori B ,Mika O ,Masaaki T , et al. Amodiaquine derivatives as inhibitors of severe fever with thrombocytopenia syndrome virus (SFTSV) replication. Antiviral Research, 2023, 210:105479.

[105] Shu S ,Yaxian Z ,Zhiyun Y , et al. Antiviral activity and mechanism of the antifungal drug, anidulafungin, suggesting its potential to promote treatment of viral diseases. BMC Medicine, 2022, 20(1):359.

[106] Kun Y, Qilue W, Zhirong Z, et al. Epidemiological characteristics and spatiotemporal aggregation analysis of severe fever with thrombocytopenia syndrome in Maanshan City,Anhui Province from 2013 to 2022. Disease Surveillance, 2023, 39(07):831-835.

[107] Lei C ,Tingting C ,Ruidong L , et al. Recent Advances in the Study of the Immune Escape Mechanism of SFTSV and Its Therapeutic Agents. Viruses, 2023, 15(4):940.

[108] Ruting Z, Jianxia Z. Analysis of Case Characteristics and Vector Tick Surveillance Results of Fever with Thrombocytopenia Syndrome in Lishui City in 2021. Chinese Journal of Control of Endemic Diseases, 2022, 37(06):472-473.

[109] Tomoki Y . Vaccine Development for Severe Fever with Thrombocytopenia Syndrome. Viruses, 2021, 13(4):627-627.

[110] Xiaoxia H, Aqian L, Dexin L, et al. Epidemiological analysis of severe fever with throml bocytopenia syndrome in 2018,China. Chinese Journal of Viral Diseases, 2020, 10(06):417-420.

[111] Guoying Z, Qi S, Jiying J, et al. Epidemiological characteristics and mortality factors of 610 cases of severe fever with thrombocytopenia syndrome in Weihai city, Shandong province from 2018 to 2021. Modern Disease Control and Prevention, 2023, 34(02):91-94+108.

[112] Huirong L, Yanyan Y, Jing Z, et al. AnaIysis of the epidemioIogicaI and pathogenic characteristics of 1 461 cases of severe fever with thrombocytopenia syndrome in Xinyang of Henan, 2018-2021. Modern Disease Control and Prevention, 2022, 33(10):744-747.

[113] Jinxia C. Epidemiology of clustered cases of fever with thrombocytopenia syndrome. Modern Disease Control and Prevention, 2020, 31(09):724-726.

[114] Lan Z, Chun Y, Yuanyi M, et al. Analysis of 992 cases of severe fever with thrombocytopenia syndrome in Xinyang City. Anhui Journal of Preventive Medicine, 2021, 27(06):462-465+482.

[115] Jixu L, Shinhyeong Cho, Wen P, et al. Surveillance and analysis of Haemaphysalis longicornis with SFTS virus in Tumen river basin located in the frontiers of Ｒussia,Korea and Northeast China. Chinese Journal of Disease Control and Prevention, 2019, 23(02):201-206+211-212.

[116] Xiantong Z, Jimin S, Feng L, et al. Analysis of reported cases of fever with thrombocytopenia syndrome and tick vectors surveillance results in Zhejiang province of China in 2021. Chinese Journal of Vector Biology and Control, 2022, 33(04):485-488.

[117] Yanhai H, Yinghua L, Zhaomei X, et al. Investigation on the antibody level of severe fever with thrombocytopenia syndrome bunyavirus in common livestock in Tiantai County. Chinese Journal of Health Laboratory Technology, 2021, 31(22):2773-2776.

[118] Lijing Z, Weiwei B, Meizhai L, et al. Epidemiological investigation on a case of fever with thrombocytopenia syndrome. Shanghai Journal of Preventive Medicine, 2019, 31(07):577-579.

[119] Yuan C, Jibo Y, Shuier S, et al. An analysis of severe fever with thrombocytopenia syndrome infected by Bunyamwera virus in Zhoushan Area. Chinese Journal of Health Laboratory Technology, 2019, 29(19):2414-2417.

[120] Tao C, Gongfei W, Haoming D, et al. Detection and genotype distribution characteristics of severe fever with thrombocytopenia syndrome bunyavirus samples from sentinel hospital in Wuhu City. Anhui Journal of Preventive Medicine, 2023, 29(05):378-381+393.

[121] Yaling F, Lanqin H, Xineng J, et al. Investigation on the first case of fever with thrombocytopenia syndrome in Lanxi City. China Preventive Medicine Journal, 2021, 33(03):284-285.

[122] Zhigang Z. Clinical analysis of 34 cases of fever with thrombocytopenia syndrome. Capital Food Medicine, 2020, 27(02):26.

[123] Yi S, Wenzhi L, Qun L, et al. Clinical analysis of 17 cases of severe fever with thrombocytopenia syndrome. Journal of Dalian Medical University 2020, 2020, 42(01):47-50+70.

[124] Yimeng Z, Yao L, Yue L, et al. Clinical analysis of 40 cases of fever with thrombocytopenia syndrome Chin J Diffic and Compl Cas, 2019, Vol． 18 No． 7. Chinese Journal of Difficult and Complicated Cases, 2019, 18(07):692-695.

[125] Zhengwen Y, Keyu W, Weishun Z, et al. Epidemiological survey of Severe fever with thrombocytopenia syndrome in Chaohu City from 2018 to 2022. Anhui Journal of Preventive Medicine, 2023, 29(03):209-212+262.

[126] Caiyun Q, Shang G, Chaoxia S, et al. Epidemiological characteristics and spatio-temporal clustering analysis of severe fever with thrombocytopenia syndrome in Jinan city in the past 10 years. Chinese Journal of Zoonoses, 2022, 38(02):135-140.

[127] Yuqing Q, Weiyun Z. Epidemiological characteristics analysis of fever with thrombocytopenia syndrome in Zhangqiu District, Jinan City from 2016 to 2020. Chinese Journal of Rural Medicine and Pharmacy, 2023, 30(17):72-74.

[128] Dawei Z. Epidemiological Characteristics And Ecologicainiche Model Of Severe Fever With Thrombocytopeniasyndrome In Jiangsu Province. Southeast University, 2019.

[129] Yulan S, Zhiyue W, Lijun S, et al. Pathogenetic characteristics of the first local case of severe fever with thrombocytopenia syndrome in Beijing. Disease Surveillance, 2023, 38(10):1186-1192.

[130] Jinsheng W, Lei G, Xianxiang L, et al. Epidemiological investigation of 137 cases of severe fever with thrombocytopenia syndrome and monitoring results of vehicle ticks in Anqing City. Occup and Health, 2019, 35(04):516-520.

[131] Min H, Limin S, Mengkai Q, et al. Pathogenic detection and epidemiological characteristics analysis of fever with thrombocytopenia syndrome in Nanjing in 2016. ACTA UNIVERSITATIS MEDICINALIS NANJING, 2018, 38(12):1829-1831.

[132] Lan W, Jianjun Z, Guangming X, et al. Analysis of three family clusters of severe fever with thrombocytopenia syndrome by exposure risk matrix. China

Preventive Medicine Journal, 2020, 32(02):125-129.
[133] Xiaolin J, Xiaomei Z, Bo P, et al. Temporal-spatial Analysis of severe fever with thrombocytopenia syndrome（SFTS）in Shandong Province,China. Chinese Journal of Zoonoses, 2020, 36(09):740-745.
[134] Jinxia C, Yan H, Jing X, et al. Analysis of epidemic characteristics of fever with platelet syndrome in Luoshan county from 2010 to 2019. Journal of Medical Forum, 2020, 41(05):79-83.
[135] Lan W, Jianjun Z, Yu X, et al. Analysis of Characteristics of Fever with Thrombocytopenia Syndrome Cases in Linhai City. China Preventive Medicine Journal, 2019, 31(05):482-483+487.
[136] Zong L, Xuesheng L, Lingling M, et al. Prevalence and M-fragment gene sequencing of fever with thrombocytopenia syndrome virus in Liaoning province, 2011 – 2017. Chinese Journal of Public Health, 2019, 35(05):644-647.
[137] Jixu L, Xing Z, Guangxing J, et al. Investigation of free ticks carrying CＲT and compound infection with SFTSV in Yanbian area of Jilin Province. Chinese Journal of Disease Control and Prevention, 2019, 23(05):561-566+572.
[138] Jianglin L, Yafei H, Jian W, et al. Analysis of the Epidemic Characteristics of Fever with Thrombocytopenia Syndrome in Taizhou City. China Preventive Medicine Journal, 2019, 31(12):1267-1268+1272.
[139] Meng Z. Clinical Characteristics And Riskfactors Of Severe Fever Withthrombocytopenia Syndrome. Shandong First Medical University, 2019.
[140] Aiguo Y, Li L, Xiaoyang W, et al. Epidemiological investigation and pathogenic characteristics of a cluster of severe fever with thrombocytopenia syndrome in Henan. Modern Disease Control and Prevention, 2023, 34(10):740-745.
[141] Jingzhong W, Hongbiao C, Zhiqiang P, et al. The first case of severe fever with thrombocytopenia syndrome in Guang dong Province. Chinese Journal of Infection Control, , 20(01):86-90.
[142] Tao M, Min Z, Xuefei D, et al. The rising trend of severe fever with thrombocytopenia syndrome: based on the data of reported cases in Nanjing from 2015 to 2020,China. Chinese Journal of Disease Control and Prevention, 2022, 26(12):1407-1413.
[143] Minah Y ,Kwangmin Y ,Sujin P , et al. Seroprevalence of Severe Fever with Thrombocytopenia Syndrome Phlebovirus in Domesticated Deer in South Korea. Virologica Sinica, 2019, 34(5):501-507.
[144] Jing C ,Li Z ,Bing H, et al. Prevalence and Molecular Phylogenetic Analysis of Severe Fever with Thrombocytopenia Syndrome Virus in Domestic Animals and Rodents in Hubei Province, China. Virologica Sinica, 2019, 34(5): 596-600.
[145] Han S, Yongzhi Z, Jie C, et al. Investigation of Tick Species and Two New Pathogens Borne in Ticks in Some Tea Gardens of China. Chinese Journal of Animal Infectious Diseases, 2023, 24:1-9.

[146] Zhigao C, Lei C, Shujiang M, et al. Investigation and analysis of emergency monitoring results of a severe fever with thrombocytopenia syndrome in Shenzhen. China Tropical Medicine, 2022, 22(03):284-288.
[147] Jinzhen N, Fangqin H, Jiling W, et al. Clinical characteristics and emergency management strategies of fever with thrombocytopenia syndrome in Shaoxing area. Chinese Journal of Control of Endemic Diseases, 2023, 38(01):59-60.
[148] You Ag, Li Y, Li Dx, et al. Surveillance for sever fever with thrombocytopenia syndrome in Henan province, 2017-2020. Chinese Journal of Epidemiology, 2021, 42(11):2024-2029.
[149] Lee J, Moon K, Kim M, et al. Seasonal distribution of Haemaphysalis longicornis (Acari: Ixodidae) and detection of SFTS virus in Gyeongbuk Province, Republic of Korea, 2018. Acta Tropica, 2021, 221:106012.
[150] Kurihara S, Satoh A, Yu F, et al. The world first two cases of severe fever with thrombocytopenia syndrome: An epidemiological study in Nagasaki, Japan. Journal of Infection and Chemotherapy, 2016, 22(7): 461-465.
[151] Kimura T, Fukuma A, Shimojima M, et al. Seroprevalence of severe fever with thrombocytopenia syndrome (SFTS) virus antibodies in humans and animals in Ehime prefecture, Japan, an endemic region of SFTS. Journal of Infection and Chemotherapy, 2018, 24(10): 802-806.
[152] Liu H, Li Z, Wang Z, et al. The first molecular evidence of severe fever with thrombocytopenia syndrome virus in ticks in Jilin, Northeastern China. Ticks and Tick-borne Diseases, 2016, 7(6): 1280-1283.
[153] Lee H S, Kim J, Son K, et al. Phylogenetic analysis of severe fever with thrombocytopenia syndrome virus in Korean water deer (Hydropotes inermis argyropus) in the Republic of Korea. Ticks and Tick-borne Diseases, 2020, 11(2):101331.
[154] Yu K, Yu M, Park S, et al. Seroprevalence and genetic characterization of severe fever with thrombocytopenia syndrome virus in domestic goats in South Korea. Ticks and Tick-borne Diseases, 2018, 9(5): 1202-1206.
[155] Shen W, Lin H, Weng J, et al. Seroprevalence of severe fever with thrombocytopenia syndrome virus antibodies among inhabitants of Dachen Island, eastern China. Ticks and Tick-borne Diseases, 2019, 10(3): 647-650.
[156] Kim Kh, Ko M K, Kim N, et al. Seroprevalence of Severe Fever with Thrombocytopenia Syndrome in Southeastern Korea, 2015. Journal of Korean Medical Science, 2017, 32(1).
[157] Nasci R S, Brault A C, Lambert A J, et al. Novel Bunyavirus in Domestic and Captive Farmed Animals, Minnesota, USA. Emerging Infectious Diseases, 2014, 20(2): 336.
[158] Okada A, Hotta A, Kimura M, et al. A retrospective survey of the seroprevalence of severe fever with thrombocytopenia syndrome virus in wild animals in Japan. Veterinary Medicine and Science, 2020, 7(2): 600-5.

[159] Yun S, Song B, Choi W, et al. First Isolation of Severe Fever with Thrombocytopenia Syndrome Virus from Haemaphysalis longicornis Ticks Collected in Severe Fever with Thrombocytopenia Syndrome Outbreak Areas in the Republic of Korea. Vector-Borne and Zoonotic Diseases, 2016, 16(1): 66-70.

[160] Sun Y, Liu M, Luo L, et al. Seroprevalence of Severe Fever with Thrombocytopenia Syndrome Virus in Hedgehog from China. Vector-Borne and Zoonotic Diseases, 2017, 17(5): 347-350.

[161] Hirata T. Estimation of Parameters related to SFTSV (Severe Febrile Thrombocytopenia Syndrome Virus) Transmission of Sheep in Shandong, China. Science Journal of Public Health, 2014, 2(3).

[162] Li Z, Hu J, Bao C, et al. Seroprevalence of antibodies against SFTS virus infection in farmers and animals, Jiangsu, China. Journal of Clinical Virology, 2014, 60(3): 185-189.

[163] Zhang L, Sun J, Yan J, et al. Antibodies against Severe Fever with Thrombocytopenia Syndrome Virus in Healthy Persons, China, 2013. Emerging Infectious Diseases, 2014, 20(8): 1355-1357.

[164] Koga S, Takazono T, Ando T, et al. Severe Fever with Thrombocytopenia Syndrome Virus RNA in Semen, Japan. Emerging Infectious Diseases, 2019, 25(11): 2127-2128.

[165] Tran X, Yun Y, Van An L, et al. Endemic Severe Fever with Thrombocytopenia Syndrome, Vietnam. Emerging Infectious Diseases, 2019, 25(5): 1029-1031.

[166] Li H, Lu Qb, Xing B, et al. Epidemiological and clinical features of laboratory-diagnosed severe fever with thrombocytopenia syndrome in China, 2011–17: a prospective observational study. The Lancet Infectious Diseases, 2018, 18(10): 1127-1137.

[167] Sun J, Chai C, Lv H, et al. Epidemiological characteristics of severe fever with thrombocytopenia syndrome in Zhejiang Province, China. International Journal of Infectious Diseases, 2014, 25: 180-185.

[168] Zu Z, Lin H, Hu Y, et al. Seroprevalence and transmission of severe fever with thrombocytopenia syndrome virus in a coastal endemic area in Southeastern China. Ticks and Tick-borne Diseases, 2024, 15(1):102277.

[169] Kuba Y, Kyan H, Azama Y, et al. Seroepidemiological study of severe fever with thrombocytopenia syndrome in animals and humans in Okinawa, Japan. Ticks and Tick-borne Diseases, 2021, 12(6):101821.

[170] Kobayashi Y, Kato H, Yamagishi T, et al. Severe Fever with Thrombocytopenia Syndrome, Japan, 2013–2017. Emerging Infectious Diseases, 2020, 26(4): 692-699.

[171] Xing Z, Kato H, Yamagishi T, et al. Epidemiological and Clinical Features of Severe Fever with Thrombocytopenia Syndrome in Japan, 2013–2014. Public Library of Science ONE, 2016, 11(10):e0165207.

[172] Fung I Ch, Chen R, Kou Z, et al. Analysis of epidemiological characteristics of four natural-focal diseases in Shandong Province, China in 2009-2017: A

descriptive analysis. Public Library of Science ONE, 2019, 14(8):e0221677.
[173] Han S, An J, Rim J, et al. Confirmed cases of severe fever with thrombocytopenia syndrome in companion cats with a history of tick exposure in the Republic of Korea. Journal of Veterinary Science, 2022, 23(6):e83.
[174] Han Sw, An Jh, Rim Jm, et al. Confirmed cases of severe fever with thrombocytopenia syndrome in companion cats with a history of tick exposure in the Republic of Korea. Journal of Veterinary Science, 2022, 23(6):e83.
[175] Zhang Q, Liu W, Wang W, et al. Analysis of spatial-temporal distribution characteristics and natural infection status of SFTS cases in Hefei from 2015 to 2021. Environmental Health and Preventive Medicine, 2023, 28: 70.
[176] Rattanakomol P, Khongwichit S, Linsuwanon P, et al. Severe Fever with Thrombocytopenia Syndrome Virus Infection, Thailand, 2019–2020. Emerging Infectious Diseases, 2022, 28(12): 2572-2574.
[177] Yoo J R, Heo S T, Song S W, et al. Severe Fever with Thrombocytopenia Syndrome Virus in Ticks and SFTS Incidence in Humans, South Korea. Emerging Infectious Diseases, 2020, 26(9): 2292-2574.
[178] Foley J, Li Z, Bao C, et al. Ecology of the Tick-Borne Phlebovirus Causing Severe Fever with Thrombocytopenia Syndrome in an Endemic Area of China. PLOS Neglected Tropical Diseases, 2016, 10(4):e0004574.
[179] Samy A M, Du Y, Cheng N, et al. Seroprevalance of antibodies specific for severe fever with thrombocytopenia syndrome virus and the discovery of asymptomatic infections in Henan Province, China. PLOS Neglected Tropical Diseases, 2019, 13(11):e0007242.
[180] Huang Xy, Du Yh, Wang Hf, et al. Prevalence of severe fever with thrombocytopenia syndrome virus in animals in Henan Province, China. Infectious Diseases of Poverty, 2019, 8(1):56.
[181] Park S, Nam H, Na B, et al. Evaluating the spatial and temporal patterns of the severe fever thrombocytopenia syndrome in Republic of Korea. Geospatial Health, 2021, 16(2).
[182] Kim H G, Jung M, Lee Dh, et al. Seasonal activity of Haemaphysalis longicornis and Haemaphysalis flava (Acari: Ixodida), vectors of severe fever with thrombocytopenia syndrome (SFTS) virus, and their SFTS virus harboring rates in Gyeonggi Province, South Korea. Experimental and Applied Acarology, 2022, 87(1): 97-108.
[183] Zunt J R, Choi S J, Park S, et al. Severe Fever with Thrombocytopenia Syndrome in South Korea, 2013-2015. PLOS Neglected Tropical Diseases, 2016, 10(12):e0005264.
[184] Zhang Q ,Li X ,Guo J , et al. Analysis and Research on Distribution of Ticks and SFTSV and HGA Carried by Ticks in Prevalence Area in South of Henan. Proceedings of 2016 4th International Conference on Electrical&Electronics Engineering and Computer Science(ICEEECS 2016), 2008, 84.0034722222222.

[185] Tsailu L, Shanchia O, Ken M, et al. The first discovery of severe fever with thrombocytopenia syndrome virus in Taiwan. Emerging Microbes & Infections, 2020, 9(1): 148-151.
[186] You E, Wang L, Zhang L, et al. Epidemiological characteristics of severe fever with thrombocytopenia syndrome in Hefei of Anhui Province: a population-based surveillance study from 2011 to 2018. European Journal of Clinical Microbiology & Infectious Diseases, 2020, 40(5): 929-39.
[187] Kim S Y, Seo C W, Lee H I, et al. Severe fever with thrombocytopenia syndrome virus from ticks: a molecular epidemiological study of a patient in the Republic of Korea. Experimental and Applied Acarology, 2023, 89(2): 305-15.
[188] Wang Y, Pang B, Ma W, et al. Spatiotemporal analysis of severe fever with thrombocytopenia syndrome in Shandong Province, China, 2014–2018. BMC Public Health, 2022, 22(1):1998.
[189] Han S, Oh Y, Rim J, et al. Clinical features and epidemiology of severe fever with thrombocytopenia syndrome in dogs in the Republic of Korea: an observational study (2019–2020). Veterinary Research Communications, 2022, 46(4): 1195-207.
[190] Crump A, Tanimoto T, et al. Severe Fever with Thrombocytopenia Syndrome: Japan under Threat from Life-threatening Emerging Tick-borne Disease. Journal of Magnesium and Alloys, 2020, 3(4):295-302.
[191] Qiulan C, Ning C, Dong Y, et al. Epidemiological characteristics of severe fever with thtrombocytopenia syndrome in China, 2011-2021. Chinese Journal of Epidemiology, 2020, 43(6):852-859.
[192] Wang G, Wang J, Tian F, et al. Severe Fever with Thrombocytopenia Syndrome Virus Infection in Minks in China. Vector-Borne and Zoonotic Diseases, 2017, 17(8):596-598.
[193] Seo Mg, Noh Be, Lee H S, et al. Nationwide Temporal and Geographical Distribution of Tick Populations and Phylogenetic Analysis of Severe Fever with Thrombocytopenia Syndrome Virus in Ticks in Korea, 2020. Microorganisms, 2021, 9(8): 1630.
[194] Tao M, Liu Y, Ling F, et al. Severe Fever With Thrombocytopenia Syndrome in Southeastern China, 2011–2019. Frontiers in Public Health, 2022, 9: 803660.
