# Supplementary material for: Analysis of Gene Differences Between F and B Epidemic Lineages of Bandavirus Dabieense
Source: Microorganisms. 2025 Jan 28;13(2):292. doi: 10.3390/microorganisms13020292 (PMC11857831; doi:10.3390/microorganisms13020292)
Supplement: Supplementary file 1 [file microorganisms-13-00292-s001.zip › Supplementary Table 2.pdf]

Supplementary Table 2:

S Segment Lineage Division

number	country	host	collection year	lineage
MT114264.1	China	Homo sapiens	2011	A
OM451561.1	China	Homo sapiens	2012	A
OM451572.1	China	Homo sapiens	2012	A
OM451578.1	China	Homo sapiens	2012	A
OM451580.1	China	Homo sapiens	2012	A
MT114283.1	China	Homo sapiens	2012	A
MT114284.1	China	Homo sapiens	2012	A
OM451588.1	China	Homo sapiens	2013	A
OM451593.1	China	Homo sapiens	2013	A
OM451610.1	China	Homo sapiens	2013	A
OM451619.1	China	Homo sapiens	2013	A
OM451620.1	China	Homo sapiens	2013	A
OM451623.1	China	Homo sapiens	2013	A
OM451640.1	China	Homo sapiens	2013	A
OM451643.1	China	Homo sapiens	2013	A
OM451646.1	China	Homo sapiens	2013	A
OM451648.1	China	Homo sapiens	2013	A
OM451666.1	China	Homo sapiens	2013	A
OM451671.1	China	Homo sapiens	2013	A
OM451672.1	China	Homo sapiens	2013	A
OM451673.1	China	Homo sapiens	2013	A
OM451680.1	China	Homo sapiens	2013	A
OM451681.1	China	Homo sapiens	2013	A
OM451682.1	China	Homo sapiens	2013	A
MT114272.1	China	Homo sapiens	2013	A
MT114313.1	China	Homo sapiens	2013	A
LC579718.1	Japan	Procyon lotor	2013	A
LC579715.1	Japan	Procyon lotor	2013	A
OM451685.1	China	Homo sapiens	2014	A
OM451688.1	China	Homo sapiens	2014	A
OM451694.1	China	Homo sapiens	2014	A
OM451696.1	China	Homo sapiens	2014	A
OM451697.1	China	Homo sapiens	2014	A
OM451704.1	China	Homo sapiens	2014	A
OM451708.1	China	Homo sapiens	2014	A
OM451711.1	China	Homo sapiens	2014	A
OM451712.1	China	Homo sapiens	2014	A

OM451713.1	China	Homo sapiens	2014	A
OM451715.1	China	Homo sapiens	2014	A
OM451719.1	China	Homo sapiens	2014	A
OM451731.1	China	Homo sapiens	2014	A
OM451737.1	China	Homo sapiens	2014	A
OM451743.1	China	Homo sapiens	2014	A
OM451744.1	China	Homo sapiens	2014	A
OM451745.1	China	Homo sapiens	2014	A
OM451759.1	China	Homo sapiens	2014	A
OM451770.1	China	Homo sapiens	2014	A
OM451776.1	China	Homo sapiens	2014	A
OM451779.1	China	Homo sapiens	2014	A
KT254589.1	China	Homo sapiens	2014	A
KP339884.1	China	Homo sapiens	2014	A
KP339892.1	China	Homo sapiens	2014	A
KP339893.1	China	Homo sapiens	2014	A
KP339900.1	China	Homo sapiens	2014	A
KP339903.1	China	Homo sapiens	2014	A
KP339911.1	China	Homo sapiens	2014	A
KP339912.1	China	Homo sapiens	2014	A
KP339913.1	China	Homo sapiens	2014	A
KP339914.1	China	Homo sapiens	2014	A
KP339915.1	China	Homo sapiens	2014	A
KP339917.1	China	Homo sapiens	2014	A
KP339919.1	China	Homo sapiens	2014	A
KP339924.1	China	Homo sapiens	2014	A
KP339926.1	China	Homo sapiens	2014	A
KP339935.1	China	Homo sapiens	2014	A
KP339936.1	China	Homo sapiens	2014	A
KP339939.1	China	Homo sapiens	2014	A
OM451791.1	China	Homo sapiens	2015	A
OM451792.1	China	Homo sapiens	2015	A
OM451796.1	China	Homo sapiens	2015	A
OM451799.1	China	Homo sapiens	2015	A
OM451800.1	China	Homo sapiens	2015	A
OM451805.1	China	Homo sapiens	2015	A
OM451810.1	China	Homo sapiens	2015	A
OM451817.1	China	Homo sapiens	2015	A
OM451843.1	China	Homo sapiens	2015	A
OM451853.1	China	Homo sapiens	2015	A
OM451865.1	China	Homo sapiens	2015	A
MK513922.1	China	Homo sapiens	2015	A
KT721302.1	China	Homo sapiens	2015	A
KT721303.1	China	Homo sapiens	2015	A

KT736092.1	China	Homo sapiens	2015	A
KT736102.1	China	Homo sapiens	2015	A
KT380647.1	China	Homo sapiens	2015	A
OM451867.1	China	Homo sapiens	2016	A
OM451872.1	China	Homo sapiens	2016	A
OM451881.1	China	Homo sapiens	2016	A
OM451889.1	China	Homo sapiens	2016	A
OM451890.1	China	Homo sapiens	2016	A
OM451901.1	China	Homo sapiens	2016	A
KY965083.1	China	Homo sapiens	2016	A
OM451925.1	China	Homo sapiens	2017	A
OM451957.1	China	Homo sapiens	2017	A
OM451972.1	China	Homo sapiens	2017	A
OM451981.1	China	Homo sapiens	2017	A
OM452022.1	China	Homo sapiens	2017	A
OM452025.1	China	Homo sapiens	2017	A
OM452041.1	China	Homo sapiens	2017	A
MT320798.1	China	Homo sapiens	2017	A
OM451911.1	China	Homo sapiens	2018	A
OM451913.1	China	Homo sapiens	2018	A
OM451917.1	China	Homo sapiens	2018	A
OM451922.1	China	Homo sapiens	2018	A
OM451937.1	China	Homo sapiens	2018	A
OM451938.1	China	Homo sapiens	2018	A
OM451965.1	China	Homo sapiens	2018	A
OM451973.1	China	Homo sapiens	2018	A
OM451987.1	China	Homo sapiens	2018	A
OM451993.1	China	Homo sapiens	2018	A
OM452023.1	China	Homo sapiens	2018	A
OM452024.1	China	Homo sapiens	2018	A
LC570784.1	Japan	Dog	2018	A
OM452054.1	China	Homo sapiens	2019	A
OM452058.1	China	Homo sapiens	2019	A
OM452059.1	China	Homo sapiens	2019	A
OM452166.1	China	Homo sapiens	2019	A
OM452172.1	China	Homo sapiens	2019	A
OM452190.1	China	Homo sapiens	2019	A
OM452192.1	China	Homo sapiens	2019	A
OM452193.1	China	Homo sapiens	2019	A
OM452217.1	China	Homo sapiens	2019	A
OM452229.1	China	Homo sapiens	2019	A
OM452232.1	China	Homo sapiens	2019	A
MT232960.1	China	Homo sapiens	2019	A
LC570796.1	Japan	Dog	2019	A

OM452069.1	China	Homo sapiens	2020	A
OM452070.1	China	Homo sapiens	2020	A
OM452080.1	China	Homo sapiens	2020	A
OM452090.1	China	Homo sapiens	2020	A
OM452092.1	China	Homo sapiens	2020	A
OM452093.1	China	Homo sapiens	2020	A
OM452097.1	China	Homo sapiens	2020	A
OM452099.1	China	Homo sapiens	2020	A
OM452102.1	China	Homo sapiens	2020	A
OM452103.1	China	Homo sapiens	2020	A
OM452104.1	China	Homo sapiens	2020	A
OM452109.1	China	Homo sapiens	2020	A
OM452116.1	China	Homo sapiens	2020	A
OM452123.1	China	Homo sapiens	2020	A
OM452125.1	China	Homo sapiens	2020	A
OM452129.1	China	Homo sapiens	2020	A
OM452135.1	China	Homo sapiens	2020	A
OM452158.1	China	Homo sapiens	2020	A
OM452164.1	China	Homo sapiens	2020	A
MZ773045.1	China	Homo sapiens	2020	A
MZ773046.1	China	Homo sapiens	2020	A
MZ773047.1	China	Homo sapiens	2020	A
MZ773048.1	China	Homo sapiens	2020	A
MZ773051.1	China	Homo sapiens	2020	A
MZ773052.1	China	Homo sapiens	2020	A
MZ773053.1	China	Homo sapiens	2020	A
MZ773054.1	China	Homo sapiens	2020	A
MZ773056.1	China	Homo sapiens	2020	A
MZ773057.1	China	Homo sapiens	2020	A
KU507554.1	Korea	Homo sapiens	2013	B
KU507555.1	Korea	Homo sapiens	2013	B
KU507556.1	Korea	Homo sapiens	2013	B
KY933678.1	China	Homo sapiens	2014	B
LC579709.1	Japan	Procyon lotor	2014	B
LC579712.1	Japan	Procyon lotor	2014	B
MG920827.1	Korea	Homo sapiens	2015	B
MG921171.1	Korea	Homo sapiens	2015	B
MK513904.1	China	Homo sapiens	2015	B
MK524357.1	China	Homo sapiens	2017	B
LC462230.1	Japan	Homo sapiens	2018	B
LC462231.1	Japan	Homo sapiens	2018	B
LC570787.1	Japan	Dog	2018	B
ON840548.1	Thailand	Homo sapiens	2019	B
LC570790.1	Japan	Dog	2019	B

LC570793.1	Japan	Dog	2019	B
LC570799.1	Japan	Dog	2019	B
ON840549.1	Thailand	Homo sapiens	2020	B
ON840550.1	Thailand	Homo sapiens	2020	B
OM452088.1	China	Homo sapiens	2020	B
MZ773058.1	China	Homo sapiens	2020	B
MZ773059.1	China	Homo sapiens	2020	B
LC663817.1	Japan	Homo sapiens	2021	B
HM745932.1	China	Homo sapiens	2010	D
MT114281.1	China	Homo sapiens	2011	D
MT114291.1	China	Homo sapiens	2011	D
OM451562.1	China	Homo sapiens	2012	D
OM451575.1	China	Homo sapiens	2012	D
OM451576.1	China	Homo sapiens	2012	D
MT114282.1	China	Homo sapiens	2012	D
MT114304.1	China	Homo sapiens	2012	D
MT114305.1	China	Homo sapiens	2012	D
MT114306.1	China	Homo sapiens	2012	D
OM451585.1	China	Homo sapiens	2013	D
OM451611.1	China	Homo sapiens	2013	D
OM451615.1	China	Homo sapiens	2013	D
OM451627.1	China	Homo sapiens	2013	D
OM451629.1	China	Homo sapiens	2013	D
OM451630.1	China	Homo sapiens	2013	D
OM451650.1	China	Homo sapiens	2013	D
OM451654.1	China	Homo sapiens	2013	D
OM451656.1	China	Homo sapiens	2013	D
OM451662.1	China	Homo sapiens	2013	D
OM451679.1	China	Homo sapiens	2013	D
MT114312.1	China	Homo sapiens	2013	D
MT114314.1	China	Homo sapiens	2013	D
MT114315.1	China	Homo sapiens	2013	D
MT114316.1	China	Homo sapiens	2013	D
MT114318.1	China	Homo sapiens	2013	D
OM451690.1	China	Homo sapiens	2014	D
OM451707.1	China	Homo sapiens	2014	D
OM451717.1	China	Homo sapiens	2014	D
OM451734.1	China	Homo sapiens	2014	D
OM451735.1	China	Homo sapiens	2014	D
OM451739.1	China	Homo sapiens	2014	D
OM451747.1	China	Homo sapiens	2014	D
OM451749.1	China	Homo sapiens	2014	D
OM451754.1	China	Homo sapiens	2014	D
MT114319.1	China	Homo sapiens	2014	D

MT114321.1	China	Homo sapiens	2014	D
MT114322.1	China	Homo sapiens	2014	D
MT114325.1	China	Homo sapiens	2014	D
MT114326.1	China	Homo sapiens	2014	D
KP339891.1	China	Homo sapiens	2014	D
KP339897.1	China	Homo sapiens	2014	D
OM451787.1	China	Homo sapiens	2015	D
OM451797.1	China	Homo sapiens	2015	D
OM451809.1	China	Homo sapiens	2015	D
OM451812.1	China	Homo sapiens	2015	D
OM451813.1	China	Homo sapiens	2015	D
OM451815.1	China	Homo sapiens	2015	D
OM451823.1	China	Homo sapiens	2015	D
OM451829.1	China	Homo sapiens	2015	D
OM451846.1	China	Homo sapiens	2015	D
OM451852.1	China	Homo sapiens	2015	D
OM451860.1	China	Homo sapiens	2015	D
MT114327.1	China	Homo sapiens	2015	D
MT114328.1	China	Homo sapiens	2015	D
MT114329.1	China	Homo sapiens	2015	D
MT114330.1	China	Homo sapiens	2015	D
MT114331.1	China	Homo sapiens	2015	D
MK513931.1	China	Homo sapiens	2015	D
KY440775.1	China	Homo sapiens	2015	D
KT721320.1	China	Homo sapiens	2015	D
KT736096.1	China	Homo sapiens	2015	D
KT736097.1	China	Homo sapiens	2015	D
KT380656.1	China	Homo sapiens	2015	D
OM451782.1	China	Homo sapiens	2016	D
OM451790.1	China	Homo sapiens	2016	D
OM451880.1	China	Homo sapiens	2016	D
OM451888.1	China	Homo sapiens	2016	D
OM451894.1	China	Homo sapiens	2016	D
OM451897.1	China	Homo sapiens	2016	D
OM451902.1	China	Homo sapiens	2016	D
KY965076.1	China	Homo sapiens	2016	D
KY965078.1	China	Homo sapiens	2016	D
KY440769.1	China	Homo sapiens	2016	D
OM451903.1	China	Homo sapiens	2017	D
OM451904.1	China	Homo sapiens	2017	D
OM451905.1	China	Homo sapiens	2017	D
OM451906.1	China	Homo sapiens	2017	D
OM451907.1	China	Homo sapiens	2017	D
OM451944.1	China	Homo sapiens	2017	D

OM451991.1	China	Homo sapiens	2017	D
OM451999.1	China	Homo sapiens	2017	D
MT320789.1	China	Homo sapiens	2017	D
MT320792.1	China	Homo sapiens	2017	D
MT320795.1	China	Homo sapiens	2017	D
MT320801.1	China	Homo sapiens	2017	D
MT320813.1	China	Homo sapiens	2017	D
MT320816.1	China	Homo sapiens	2017	D
MT320819.1	China	Homo sapiens	2017	D
MK524363.1	China	Homo sapiens	2017	D
OM451916.1	China	Homo sapiens	2018	D
OM451920.1	China	Homo sapiens	2018	D
OM451943.1	China	Homo sapiens	2018	D
OM451952.1	China	Homo sapiens	2018	D
OM451959.1	China	Homo sapiens	2018	D
OM451961.1	China	Homo sapiens	2018	D
OM451971.1	China	Homo sapiens	2018	D
OM451982.1	China	Homo sapiens	2018	D
OM451985.1	China	Homo sapiens	2018	D
OM451992.1	China	Homo sapiens	2018	D
OM452014.1	China	Homo sapiens	2018	D
OM452029.1	China	Homo sapiens	2018	D
OM452031.1	China	Homo sapiens	2018	D
OM452032.1	China	Homo sapiens	2018	D
OM452039.1	China	Homo sapiens	2018	D
OM452042.1	China	Homo sapiens	2018	D
OM452047.1	China	Homo sapiens	2019	D
OM452050.1	China	Homo sapiens	2019	D
OM452053.1	China	Homo sapiens	2019	D
OM452056.1	China	Homo sapiens	2019	D
OM452165.1	China	Homo sapiens	2019	D
OM452174.1	China	Homo sapiens	2019	D
OM452214.1	China	Homo sapiens	2019	D
OM452219.1	China	Homo sapiens	2019	D
OM452235.1	China	Homo sapiens	2019	D
OM452236.1	China	Homo sapiens	2019	D
OM452239.1	China	Homo sapiens	2019	D
OM452089.1	China	Homo sapiens	2020	D
OM452110.1	China	Homo sapiens	2020	D
OM452120.1	China	Homo sapiens	2020	D
OM452136.1	China	Homo sapiens	2020	D
OM452137.1	China	Homo sapiens	2020	D
OM452138.1	China	Homo sapiens	2020	D
OM452155.1	China	Homo sapiens	2020	D

OM452241.1	China	Homo sapiens	2020	D
OM452242.1	China	Homo sapiens	2020	D
MT114290.1	China	Homo sapiens	2011	E
MT114303.1	China	Homo sapiens	2012	E
MT114308.1	China	Homo sapiens	2013	E
MT114317.1	China	Homo sapiens	2013	E
MT114320.1	China	Homo sapiens	2014	E
MT114323.1	China	Homo sapiens	2014	E
MK513907.1	China	Homo sapiens	2015	E
MK513919.1	China	Homo sapiens	2015	E
MK513928.1	China	Homo sapiens	2015	E
OM451887.1	China	Homo sapiens	2016	E
KY965082.1	China	Homo sapiens	2016	E
KY965085.1	China	Homo sapiens	2016	E
KY965087.1	China	Homo sapiens	2016	E
MK524369.1	China	Homo sapiens	2017	E
NC_018137.1	China	Homo sapiens	2010	F
MT114260.1	China	Homo sapiens	2010	F
OM451560.1	China	Homo sapiens	2011	F
MT114263.1	China	Homo sapiens	2011	F
MT114276.1	China	Homo sapiens	2011	F
MT114292.1	China	Homo sapiens	2011	F
JQ693001.1	China	Cattle	2011	F
KF358693.1	Korea	Homo sapiens	2012	F
OM451563.1	China	Homo sapiens	2012	F
OM451564.1	China	Homo sapiens	2012	F
OM451565.1	China	Homo sapiens	2012	F
OM451566.1	China	Homo sapiens	2012	F
OM451567.1	China	Homo sapiens	2012	F
OM451568.1	China	Homo sapiens	2012	F
OM451569.1	China	Homo sapiens	2012	F
OM451570.1	China	Homo sapiens	2012	F
OM451571.1	China	Homo sapiens	2012	F
OM451573.1	China	Homo sapiens	2012	F
OM451574.1	China	Homo sapiens	2012	F
OM451577.1	China	Homo sapiens	2012	F
OM451579.1	China	Homo sapiens	2012	F
OM451581.1	China	Homo sapiens	2012	F
OM451582.1	China	Homo sapiens	2012	F
OM451583.1	China	Homo sapiens	2012	F
OM451584.1	China	Homo sapiens	2012	F
MT114266.1	China	Homo sapiens	2012	F
MT114267.1	China	Homo sapiens	2012	F
MT114268.1	China	Homo sapiens	2012	F

MT114269.1	China	Homo sapiens	2012	F
MT114279.1	China	Homo sapiens	2012	F
MT114280.1	China	Homo sapiens	2012	F
MT114285.1	China	Homo sapiens	2012	F
MT114307.1	China	Homo sapiens	2012	F
KU507553.1	Korea	Homo sapiens	2013	F
KU507557.1	Korea	Homo sapiens	2013	F
OM451586.1	China	Homo sapiens	2013	F
OM451587.1	China	Homo sapiens	2013	F
OM451589.1	China	Homo sapiens	2013	F
OM451590.1	China	Homo sapiens	2013	F
OM451591.1	China	Homo sapiens	2013	F
OM451592.1	China	Homo sapiens	2013	F
OM451594.1	China	Homo sapiens	2013	F
OM451595.1	China	Homo sapiens	2013	F
OM451596.1	China	Homo sapiens	2013	F
OM451597.1	China	Homo sapiens	2013	F
OM451598.1	China	Homo sapiens	2013	F
OM451599.1	China	Homo sapiens	2013	F
OM451600.1	China	Homo sapiens	2013	F
OM451601.1	China	Homo sapiens	2013	F
OM451602.1	China	Homo sapiens	2013	F
OM451603.1	China	Homo sapiens	2013	F
OM451604.1	China	Homo sapiens	2013	F
OM451605.1	China	Homo sapiens	2013	F
OM451606.1	China	Homo sapiens	2013	F
OM451607.1	China	Homo sapiens	2013	F
OM451608.1	China	Homo sapiens	2013	F
OM451609.1	China	Homo sapiens	2013	F
OM451612.1	China	Homo sapiens	2013	F
OM451613.1	China	Homo sapiens	2013	F
OM451614.1	China	Homo sapiens	2013	F
OM451616.1	China	Homo sapiens	2013	F
OM451617.1	China	Homo sapiens	2013	F
OM451618.1	China	Homo sapiens	2013	F
OM451621.1	China	Homo sapiens	2013	F
OM451622.1	China	Homo sapiens	2013	F
OM451624.1	China	Homo sapiens	2013	F
OM451625.1	China	Homo sapiens	2013	F
OM451626.1	China	Homo sapiens	2013	F
OM451628.1	China	Homo sapiens	2013	F
OM451631.1	China	Homo sapiens	2013	F
OM451632.1	China	Homo sapiens	2013	F
OM451633.1	China	Homo sapiens	2013	F

OM451634.1	China	Homo sapiens	2013	F
OM451635.1	China	Homo sapiens	2013	F
OM451636.1	China	Homo sapiens	2013	F
OM451637.1	China	Homo sapiens	2013	F
OM451638.1	China	Homo sapiens	2013	F
OM451639.1	China	Homo sapiens	2013	F
OM451641.1	China	Homo sapiens	2013	F
OM451642.1	China	Homo sapiens	2013	F
OM451644.1	China	Homo sapiens	2013	F
OM451645.1	China	Homo sapiens	2013	F
OM451647.1	China	Homo sapiens	2013	F
OM451649.1	China	Homo sapiens	2013	F
OM451651.1	China	Homo sapiens	2013	F
OM451652.1	China	Homo sapiens	2013	F
OM451653.1	China	Homo sapiens	2013	F
OM451655.1	China	Homo sapiens	2013	F
OM451657.1	China	Homo sapiens	2013	F
OM451658.1	China	Homo sapiens	2013	F
OM451659.1	China	Homo sapiens	2013	F
OM451660.1	China	Homo sapiens	2013	F
OM451661.1	China	Homo sapiens	2013	F
OM451663.1	China	Homo sapiens	2013	F
OM451664.1	China	Homo sapiens	2013	F
OM451665.1	China	Homo sapiens	2013	F
OM451667.1	China	Homo sapiens	2013	F
OM451668.1	China	Homo sapiens	2013	F
OM451669.1	China	Homo sapiens	2013	F
OM451670.1	China	Homo sapiens	2013	F
OM451674.1	China	Homo sapiens	2013	F
OM451675.1	China	Homo sapiens	2013	F
OM451676.1	China	Homo sapiens	2013	F
OM451677.1	China	Homo sapiens	2013	F
OM451678.1	China	Homo sapiens	2013	F
OM451683.1	China	Homo sapiens	2013	F
MT114270.1	China	Homo sapiens	2013	F
MT114271.1	China	Homo sapiens	2013	F
MT114273.1	China	Homo sapiens	2013	F
MT114274.1	China	Homo sapiens	2013	F
MT114275.1	China	Homo sapiens	2013	F
MT114287.1	China	Homo sapiens	2013	F
MT114309.1	China	Homo sapiens	2013	F
MT114310.1	China	Homo sapiens	2013	F
MT114311.1	China	Homo sapiens	2013	F
KU361341.1	China	Homo sapiens	2013	F

MZ501603.1	Korea	Goat	2014	F
NC_043452.1	China	Homo sapiens	2014	F
OM451684.1	China	Homo sapiens	2014	F
OM451686.1	China	Homo sapiens	2014	F
OM451687.1	China	Homo sapiens	2014	F
OM451689.1	China	Homo sapiens	2014	F
OM451691.1	China	Homo sapiens	2014	F
OM451692.1	China	Homo sapiens	2014	F
OM451693.1	China	Homo sapiens	2014	F
OM451695.1	China	Homo sapiens	2014	F
OM451698.1	China	Homo sapiens	2014	F
OM451699.1	China	Homo sapiens	2014	F
OM451700.1	China	Homo sapiens	2014	F
OM451701.1	China	Homo sapiens	2014	F
OM451702.1	China	Homo sapiens	2014	F
OM451703.1	China	Homo sapiens	2014	F
OM451705.1	China	Homo sapiens	2014	F
OM451706.1	China	Homo sapiens	2014	F
OM451709.1	China	Homo sapiens	2014	F
OM451710.1	China	Homo sapiens	2014	F
OM451714.1	China	Homo sapiens	2014	F
OM451716.1	China	Homo sapiens	2014	F
OM451718.1	China	Homo sapiens	2014	F
OM451721.1	China	Homo sapiens	2014	F
OM451722.1	China	Homo sapiens	2014	F
OM451723.1	China	Homo sapiens	2014	F
OM451724.1	China	Homo sapiens	2014	F
OM451725.1	China	Homo sapiens	2014	F
OM451727.1	China	Homo sapiens	2014	F
OM451728.1	China	Homo sapiens	2014	F
OM451729.1	China	Homo sapiens	2014	F
OM451730.1	China	Homo sapiens	2014	F
OM451732.1	China	Homo sapiens	2014	F
OM451733.1	China	Homo sapiens	2014	F
OM451736.1	China	Homo sapiens	2014	F
OM451741.1	China	Homo sapiens	2014	F
OM451742.1	China	Homo sapiens	2014	F
OM451746.1	China	Homo sapiens	2014	F
OM451748.1	China	Homo sapiens	2014	F
OM451750.1	China	Homo sapiens	2014	F
OM451751.1	China	Homo sapiens	2014	F
OM451752.1	China	Homo sapiens	2014	F
OM451753.1	China	Homo sapiens	2014	F
OM451755.1	China	Homo sapiens	2014	F

OM451756.1	China	Homo sapiens	2014	F
OM451757.1	China	Homo sapiens	2014	F
OM451758.1	China	Homo sapiens	2014	F
OM451760.1	China	Homo sapiens	2014	F
OM451761.1	China	Homo sapiens	2014	F
OM451762.1	China	Homo sapiens	2014	F
OM451763.1	China	Homo sapiens	2014	F
OM451764.1	China	Homo sapiens	2014	F
OM451765.1	China	Homo sapiens	2014	F
OM451766.1	China	Homo sapiens	2014	F
OM451767.1	China	Homo sapiens	2014	F
OM451768.1	China	Homo sapiens	2014	F
OM451769.1	China	Homo sapiens	2014	F
OM451771.1	China	Homo sapiens	2014	F
OM451773.1	China	Homo sapiens	2014	F
OM451774.1	China	Homo sapiens	2014	F
OM451775.1	China	Homo sapiens	2014	F
OM451777.1	China	Homo sapiens	2014	F
OM451778.1	China	Homo sapiens	2014	F
OM451780.1	China	Homo sapiens	2014	F
OM451781.1	China	Homo sapiens	2014	F
MT114289.1	China	Homo sapiens	2014	F
MT114324.1	China	Homo sapiens	2014	F
MT309106.1	China	Homo sapiens	2014	F
MT309107.1	China	Homo sapiens	2014	F
KY933675.1	China	Homo sapiens	2014	F
KY933676.1	China	Homo sapiens	2014	F
KY933677.1	China	Homo sapiens	2014	F
KY933679.1	China	Homo sapiens	2014	F
KY933680.1	China	Homo sapiens	2014	F
KR706565.1	China	Homo sapiens	2014	F
KP984502.1	China	Homo sapiens	2014	F
KP339885.1	China	Homo sapiens	2014	F
KP339886.1	China	Homo sapiens	2014	F
KP339887.1	China	Homo sapiens	2014	F
KP339888.1	China	Homo sapiens	2014	F
KP339889.1	China	Homo sapiens	2014	F
KP339890.1	China	Homo sapiens	2014	F
KP339894.1	China	Homo sapiens	2014	F
KP339895.1	China	Homo sapiens	2014	F
KP339896.1	China	Homo sapiens	2014	F
KP339898.1	China	Homo sapiens	2014	F
KP339899.1	China	Homo sapiens	2014	F
KP339901.1	China	Homo sapiens	2014	F

KP339902.1	China	Homo sapiens	2014	F
KP339904.1	China	Homo sapiens	2014	F
KP339905.1	China	Homo sapiens	2014	F
KP339906.1	China	Homo sapiens	2014	F
KP339907.1	China	Homo sapiens	2014	F
KP339908.1	China	Homo sapiens	2014	F
KP339909.1	China	Homo sapiens	2014	F
KP339910.1	China	Homo sapiens	2014	F
KP339916.1	China	Homo sapiens	2014	F
KP339920.1	China	Homo sapiens	2014	F
KP339921.1	China	Homo sapiens	2014	F
KP339922.1	China	Homo sapiens	2014	F
KP339923.1	China	Homo sapiens	2014	F
KP339925.1	China	Homo sapiens	2014	F
KP339927.1	China	Homo sapiens	2014	F
KP339928.1	China	Homo sapiens	2014	F
KP339929.1	China	Homo sapiens	2014	F
KP339930.1	China	Homo sapiens	2014	F
KP339931.1	China	Homo sapiens	2014	F
KP339932.1	China	Homo sapiens	2014	F
KP339933.1	China	Homo sapiens	2014	F
KP339934.1	China	Homo sapiens	2014	F
KP339937.1	China	Homo sapiens	2014	F
KP339938.1	China	Homo sapiens	2014	F
KP339940.1	China	Homo sapiens	2014	F
KP339941.1	China	Homo sapiens	2014	F
MN329148.1	Korea	Homo sapiens	2015	F
MH491547.1	Korea	Homo sapiens	2015	F
MG920821.1	Korea	Homo sapiens	2015	F
MG920824.1	Korea	Homo sapiens	2015	F
MG920830.1	Korea	Homo sapiens	2015	F
MG921168.1	Korea	Homo sapiens	2015	F
OM451783.1	China	Homo sapiens	2015	F
OM451784.1	China	Homo sapiens	2015	F
OM451785.1	China	Homo sapiens	2015	F
OM451786.1	China	Homo sapiens	2015	F
OM451788.1	China	Homo sapiens	2015	F
OM451789.1	China	Homo sapiens	2015	F
OM451793.1	China	Homo sapiens	2015	F
OM451794.1	China	Homo sapiens	2015	F
OM451795.1	China	Homo sapiens	2015	F
OM451798.1	China	Homo sapiens	2015	F
OM451801.1	China	Homo sapiens	2015	F
OM451802.1	China	Homo sapiens	2015	F

OM451803.1	China	Homo sapiens	2015	F
OM451804.1	China	Homo sapiens	2015	F
OM451806.1	China	Homo sapiens	2015	F
OM451807.1	China	Homo sapiens	2015	F
OM451808.1	China	Homo sapiens	2015	F
OM451811.1	China	Homo sapiens	2015	F
OM451814.1	China	Homo sapiens	2015	F
OM451816.1	China	Homo sapiens	2015	F
OM451818.1	China	Homo sapiens	2015	F
OM451819.1	China	Homo sapiens	2015	F
OM451820.1	China	Homo sapiens	2015	F
OM451821.1	China	Homo sapiens	2015	F
OM451822.1	China	Homo sapiens	2015	F
OM451824.1	China	Homo sapiens	2015	F
OM451825.1	China	Homo sapiens	2015	F
OM451826.1	China	Homo sapiens	2015	F
OM451827.1	China	Homo sapiens	2015	F
OM451828.1	China	Homo sapiens	2015	F
OM451830.1	China	Homo sapiens	2015	F
OM451831.1	China	Homo sapiens	2015	F
OM451832.1	China	Homo sapiens	2015	F
OM451833.1	China	Homo sapiens	2015	F
OM451834.1	China	Homo sapiens	2015	F
OM451835.1	China	Homo sapiens	2015	F
OM451836.1	China	Homo sapiens	2015	F
OM451837.1	China	Homo sapiens	2015	F
OM451838.1	China	Homo sapiens	2015	F
OM451840.1	China	Homo sapiens	2015	F
OM451842.1	China	Homo sapiens	2015	F
OM451844.1	China	Homo sapiens	2015	F
OM451845.1	China	Homo sapiens	2015	F
OM451847.1	China	Homo sapiens	2015	F
OM451848.1	China	Homo sapiens	2015	F
OM451849.1	China	Homo sapiens	2015	F
OM451850.1	China	Homo sapiens	2015	F
OM451851.1	China	Homo sapiens	2015	F
OM451854.1	China	Homo sapiens	2015	F
OM451855.1	China	Homo sapiens	2015	F
OM451856.1	China	Homo sapiens	2015	F
OM451857.1	China	Homo sapiens	2015	F
OM451858.1	China	Homo sapiens	2015	F
OM451859.1	China	Homo sapiens	2015	F
OM451861.1	China	Homo sapiens	2015	F
OM451862.1	China	Homo sapiens	2015	F

OM451863.1	China	Homo sapiens	2015	F
OM451864.1	China	Homo sapiens	2015	F
OM451866.1	China	Homo sapiens	2015	F
MK513913.1	China	Homo sapiens	2015	F
MK513916.1	China	Homo sapiens	2015	F
MK513925.1	China	Homo sapiens	2015	F
MF140449.1	China	Rat	2015	F
KT721293.1	China	Homo sapiens	2015	F
KT721294.1	China	Homo sapiens	2015	F
KT721295.1	China	Homo sapiens	2015	F
KT721296.1	China	Homo sapiens	2015	F
KT721297.1	China	Homo sapiens	2015	F
KT721298.1	China	Homo sapiens	2015	F
KT721299.1	China	Homo sapiens	2015	F
KT721300.1	China	Homo sapiens	2015	F
KT721301.1	China	Homo sapiens	2015	F
KT721304.1	China	Homo sapiens	2015	F
KT721305.1	China	Homo sapiens	2015	F
KT721306.1	China	Homo sapiens	2015	F
KT721307.1	China	Homo sapiens	2015	F
KT721308.1	China	Homo sapiens	2015	F
KT721309.1	China	Homo sapiens	2015	F
KT721310.1	China	Homo sapiens	2015	F
KT721311.1	China	Homo sapiens	2015	F
KT721312.1	China	Homo sapiens	2015	F
KT721313.1	China	Homo sapiens	2015	F
KT721314.1	China	Homo sapiens	2015	F
KT721315.1	China	Homo sapiens	2015	F
KT721316.1	China	Homo sapiens	2015	F
KT721317.1	China	Homo sapiens	2015	F
KT721318.1	China	Homo sapiens	2015	F
KT721319.1	China	Homo sapiens	2015	F
KT736090.1	China	Homo sapiens	2015	F
KT736091.1	China	Homo sapiens	2015	F
KT736093.1	China	Homo sapiens	2015	F
KT736094.1	China	Homo sapiens	2015	F
KT736095.1	China	Homo sapiens	2015	F
KT736098.1	China	Homo sapiens	2015	F
KT736099.1	China	Homo sapiens	2015	F
KT736100.1	China	Homo sapiens	2015	F
KT736101.1	China	Homo sapiens	2015	F
KT380644.1	China	Homo sapiens	2015	F
KT380645.1	China	Homo sapiens	2015	F
KT380646.1	China	Homo sapiens	2015	F

KT380648.1	China	Homo sapiens	2015	F
KT380649.1	China	Homo sapiens	2015	F
KT380650.1	China	Homo sapiens	2015	F
KT380651.1	China	Homo sapiens	2015	F
KT380652.1	China	Homo sapiens	2015	F
KT380653.1	China	Homo sapiens	2015	F
KT380654.1	China	Homo sapiens	2015	F
KT380655.1	China	Homo sapiens	2015	F
KT380657.1	China	Homo sapiens	2015	F
KT380658.1	China	Homo sapiens	2015	F
KT380659.1	China	Homo sapiens	2015	F
OM451841.1	China	Homo sapiens	2016	F
OM451868.1	China	Homo sapiens	2016	F
OM451869.1	China	Homo sapiens	2016	F
OM451870.1	China	Homo sapiens	2016	F
OM451871.1	China	Homo sapiens	2016	F
OM451873.1	China	Homo sapiens	2016	F
OM451874.1	China	Homo sapiens	2016	F
OM451875.1	China	Homo sapiens	2016	F
OM451876.1	China	Homo sapiens	2016	F
OM451877.1	China	Homo sapiens	2016	F
OM451878.1	China	Homo sapiens	2016	F
OM451879.1	China	Homo sapiens	2016	F
OM451882.1	China	Homo sapiens	2016	F
OM451883.1	China	Homo sapiens	2016	F
OM451884.1	China	Homo sapiens	2016	F
OM451885.1	China	Homo sapiens	2016	F
OM451886.1	China	Homo sapiens	2016	F
OM451891.1	China	Homo sapiens	2016	F
OM451892.1	China	Homo sapiens	2016	F
OM451893.1	China	Homo sapiens	2016	F
OM451895.1	China	Homo sapiens	2016	F
OM451896.1	China	Homo sapiens	2016	F
OM451898.1	China	Homo sapiens	2016	F
OM451899.1	China	Homo sapiens	2016	F
OM451900.1	China	Homo sapiens	2016	F
KY965075.1	China	Homo sapiens	2016	F
KY965077.1	China	Homo sapiens	2016	F
KY965080.1	China	Homo sapiens	2016	F
KY965081.1	China	Homo sapiens	2016	F
KY965084.1	China	Homo sapiens	2016	F
KY965086.1	China	Homo sapiens	2016	F
KY965088.1	China	Homo sapiens	2016	F
KY965089.1	China	Homo sapiens	2016	F

KY965090.1	China	Homo sapiens	2016	F
KY965091.1	China	Homo sapiens	2016	F
KY440772.1	China	Homo sapiens	2016	F
MT683685.1	Korea	Homo sapiens	2017	F
MH937372.1	Korea	Homo sapiens	2017	F
OM451908.1	China	Homo sapiens	2017	F
OM451909.1	China	Homo sapiens	2017	F
OM451912.1	China	Homo sapiens	2017	F
OM451914.1	China	Homo sapiens	2017	F
OM451915.1	China	Homo sapiens	2017	F
OM451919.1	China	Homo sapiens	2017	F
OM451921.1	China	Homo sapiens	2017	F
OM451923.1	China	Homo sapiens	2017	F
OM451926.1	China	Homo sapiens	2017	F
OM451930.1	China	Homo sapiens	2017	F
OM451931.1	China	Homo sapiens	2017	F
OM451932.1	China	Homo sapiens	2017	F
OM451942.1	China	Homo sapiens	2017	F
OM451949.1	China	Homo sapiens	2017	F
OM451954.1	China	Homo sapiens	2017	F
OM451979.1	China	Homo sapiens	2017	F
OM451983.1	China	Homo sapiens	2017	F
OM451988.1	China	Homo sapiens	2017	F
OM451989.1	China	Homo sapiens	2017	F
OM451990.1	China	Homo sapiens	2017	F
OM451994.1	China	Homo sapiens	2017	F
OM451995.1	China	Homo sapiens	2017	F
OM451996.1	China	Homo sapiens	2017	F
OM452000.1	China	Homo sapiens	2017	F
OM452001.1	China	Homo sapiens	2017	F
OM452003.1	China	Homo sapiens	2017	F
OM452007.1	China	Homo sapiens	2017	F
OM452010.1	China	Homo sapiens	2017	F
OM452012.1	China	Homo sapiens	2017	F
OM452018.1	China	Homo sapiens	2017	F
OM452028.1	China	Homo sapiens	2017	F
OM452033.1	China	Homo sapiens	2017	F
OM452036.1	China	Homo sapiens	2017	F
OM452037.1	China	Homo sapiens	2017	F
OM452044.1	China	Homo sapiens	2017	F
MT320804.1	China	Homo sapiens	2017	F
MT320807.1	China	Homo sapiens	2017	F
MT320810.1	China	Homo sapiens	2017	F
MK524351.1	China	Homo sapiens	2017	F

MK524354.1	China	Homo sapiens	2017	F
MK524360.1	China	Homo sapiens	2017	F
MK524366.1	China	Homo sapiens	2017	F
MK524372.1	China	Homo sapiens	2017	F
MK300947.1	China	Homo sapiens	2017	F
MZ501597.1	Korea	Dog	2018	F
OM451910.1	China	Homo sapiens	2018	F
OM451918.1	China	Homo sapiens	2018	F
OM451924.1	China	Homo sapiens	2018	F
OM451927.1	China	Homo sapiens	2018	F
OM451928.1	China	Homo sapiens	2018	F
OM451929.1	China	Homo sapiens	2018	F
OM451933.1	China	Homo sapiens	2018	F
OM451934.1	China	Homo sapiens	2018	F
OM451936.1	China	Homo sapiens	2018	F
OM451939.1	China	Homo sapiens	2018	F
OM451940.1	China	Homo sapiens	2018	F
OM451941.1	China	Homo sapiens	2018	F
OM451945.1	China	Homo sapiens	2018	F
OM451946.1	China	Homo sapiens	2018	F
OM451947.1	China	Homo sapiens	2018	F
OM451948.1	China	Homo sapiens	2018	F
OM451950.1	China	Homo sapiens	2018	F
OM451951.1	China	Homo sapiens	2018	F
OM451953.1	China	Homo sapiens	2018	F
OM451955.1	China	Homo sapiens	2018	F
OM451956.1	China	Homo sapiens	2018	F
OM451958.1	China	Homo sapiens	2018	F
OM451960.1	China	Homo sapiens	2018	F
OM451962.1	China	Homo sapiens	2018	F
OM451963.1	China	Homo sapiens	2018	F
OM451964.1	China	Homo sapiens	2018	F
OM451966.1	China	Homo sapiens	2018	F
OM451967.1	China	Homo sapiens	2018	F
OM451968.1	China	Homo sapiens	2018	F
OM451969.1	China	Homo sapiens	2018	F
OM451970.1	China	Homo sapiens	2018	F
OM451974.1	China	Homo sapiens	2018	F
OM451975.1	China	Homo sapiens	2018	F
OM451977.1	China	Homo sapiens	2018	F
OM451978.1	China	Homo sapiens	2018	F
OM451980.1	China	Homo sapiens	2018	F
OM451984.1	China	Homo sapiens	2018	F
OM451986.1	China	Homo sapiens	2018	F

OM451997.1	China	Homo sapiens	2018	F
OM451998.1	China	Homo sapiens	2018	F
OM452002.1	China	Homo sapiens	2018	F
OM452004.1	China	Homo sapiens	2018	F
OM452005.1	China	Homo sapiens	2018	F
OM452006.1	China	Homo sapiens	2018	F
OM452008.1	China	Homo sapiens	2018	F
OM452009.1	China	Homo sapiens	2018	F
OM452011.1	China	Homo sapiens	2018	F
OM452013.1	China	Homo sapiens	2018	F
OM452015.1	China	Homo sapiens	2018	F
OM452016.1	China	Homo sapiens	2018	F
OM452017.1	China	Homo sapiens	2018	F
OM452019.1	China	Homo sapiens	2018	F
OM452020.1	China	Homo sapiens	2018	F
OM452021.1	China	Homo sapiens	2018	F
OM452026.1	China	Homo sapiens	2018	F
OM452027.1	China	Homo sapiens	2018	F
OM452030.1	China	Homo sapiens	2018	F
OM452034.1	China	Homo sapiens	2018	F
OM452035.1	China	Homo sapiens	2018	F
OM452038.1	China	Homo sapiens	2018	F
OM452040.1	China	Homo sapiens	2018	F
OM452045.1	China	Homo sapiens	2019	F
OM452046.1	China	Homo sapiens	2019	F
OM452048.1	China	Homo sapiens	2019	F
OM452049.1	China	Homo sapiens	2019	F
OM452051.1	China	Homo sapiens	2019	F
OM452052.1	China	Homo sapiens	2019	F
OM452055.1	China	Homo sapiens	2019	F
OM452057.1	China	Homo sapiens	2019	F
OM452060.1	China	Homo sapiens	2019	F
OM452061.1	China	Homo sapiens	2019	F
OM452062.1	China	Homo sapiens	2019	F
OM452063.1	China	Homo sapiens	2019	F
OM452064.1	China	Homo sapiens	2019	F
OM452065.1	China	Homo sapiens	2019	F
OM452066.1	China	Homo sapiens	2019	F
OM452067.1	China	Homo sapiens	2019	F
OM452167.1	China	Homo sapiens	2019	F
OM452168.1	China	Homo sapiens	2019	F
OM452169.1	China	Homo sapiens	2019	F
OM452170.1	China	Homo sapiens	2019	F
OM452171.1	China	Homo sapiens	2019	F

OM452173.1	China	Homo sapiens	2019	F
OM452175.1	China	Homo sapiens	2019	F
OM452176.1	China	Homo sapiens	2019	F
OM452177.1	China	Homo sapiens	2019	F
OM452178.1	China	Homo sapiens	2019	F
OM452179.1	China	Homo sapiens	2019	F
OM452180.1	China	Homo sapiens	2019	F
OM452181.1	China	Homo sapiens	2019	F
OM452182.1	China	Homo sapiens	2019	F
OM452183.1	China	Homo sapiens	2019	F
OM452184.1	China	Homo sapiens	2019	F
OM452185.1	China	Homo sapiens	2019	F
OM452186.1	China	Homo sapiens	2019	F
OM452187.1	China	Homo sapiens	2019	F
OM452188.1	China	Homo sapiens	2019	F
OM452189.1	China	Homo sapiens	2019	F
OM452191.1	China	Homo sapiens	2019	F
OM452194.1	China	Homo sapiens	2019	F
OM452195.1	China	Homo sapiens	2019	F
OM452196.1	China	Homo sapiens	2019	F
OM452197.1	China	Homo sapiens	2019	F
OM452198.1	China	Homo sapiens	2019	F
OM452199.1	China	Homo sapiens	2019	F
OM452200.1	China	Homo sapiens	2019	F
OM452201.1	China	Homo sapiens	2019	F
OM452202.1	China	Homo sapiens	2019	F
OM452203.1	China	Homo sapiens	2019	F
OM452204.1	China	Homo sapiens	2019	F
OM452205.1	China	Homo sapiens	2019	F
OM452206.1	China	Homo sapiens	2019	F
OM452207.1	China	Homo sapiens	2019	F
OM452208.1	China	Homo sapiens	2019	F
OM452209.1	China	Homo sapiens	2019	F
OM452210.1	China	Homo sapiens	2019	F
OM452211.1	China	Homo sapiens	2019	F
OM452212.1	China	Homo sapiens	2019	F
OM452213.1	China	Homo sapiens	2019	F
OM452215.1	China	Homo sapiens	2019	F
OM452216.1	China	Homo sapiens	2019	F
OM452218.1	China	Homo sapiens	2019	F
OM452220.1	China	Homo sapiens	2019	F
OM452222.1	China	Homo sapiens	2019	F
OM452223.1	China	Homo sapiens	2019	F
OM452224.1	China	Homo sapiens	2019	F

OM452225.1	China	Homo sapiens	2019	F
OM452226.1	China	Homo sapiens	2019	F
OM452227.1	China	Homo sapiens	2019	F
OM452228.1	China	Homo sapiens	2019	F
OM452230.1	China	Homo sapiens	2019	F
OM452231.1	China	Homo sapiens	2019	F
OM452233.1	China	Homo sapiens	2019	F
OM452234.1	China	Homo sapiens	2019	F
OM452237.1	China	Homo sapiens	2019	F
OM452238.1	China	Homo sapiens	2019	F
OM452240.1	China	Homo sapiens	2019	F
MN830173.1	China	Homo sapiens	2019	F
MZ501600.1	Korea	Dog	2020	F
MZ342903.1	Korea	feline	2020	F
OM452068.1	China	Homo sapiens	2020	F
OM452071.1	China	Homo sapiens	2020	F
OM452072.1	China	Homo sapiens	2020	F
OM452073.1	China	Homo sapiens	2020	F
OM452074.1	China	Homo sapiens	2020	F
OM452075.1	China	Homo sapiens	2020	F
OM452076.1	China	Homo sapiens	2020	F
OM452077.1	China	Homo sapiens	2020	F
OM452078.1	China	Homo sapiens	2020	F
OM452079.1	China	Homo sapiens	2020	F
OM452081.1	China	Homo sapiens	2020	F
OM452082.1	China	Homo sapiens	2020	F
OM452083.1	China	Homo sapiens	2020	F
OM452084.1	China	Homo sapiens	2020	F
OM452085.1	China	Homo sapiens	2020	F
OM452086.1	China	Homo sapiens	2020	F
OM452087.1	China	Homo sapiens	2020	F
OM452091.1	China	Homo sapiens	2020	F
OM452094.1	China	Homo sapiens	2020	F
OM452095.1	China	Homo sapiens	2020	F
OM452096.1	China	Homo sapiens	2020	F
OM452098.1	China	Homo sapiens	2020	F
OM452100.1	China	Homo sapiens	2020	F
OM452101.1	China	Homo sapiens	2020	F
OM452105.1	China	Homo sapiens	2020	F
OM452106.1	China	Homo sapiens	2020	F
OM452107.1	China	Homo sapiens	2020	F
OM452108.1	China	Homo sapiens	2020	F
OM452111.1	China	Homo sapiens	2020	F
OM452112.1	China	Homo sapiens	2020	F

OM452113.1	China	Homo sapiens	2020	F
OM452114.1	China	Homo sapiens	2020	F
OM452115.1	China	Homo sapiens	2020	F
OM452117.1	China	Homo sapiens	2020	F
OM452118.1	China	Homo sapiens	2020	F
OM452119.1	China	Homo sapiens	2020	F
OM452121.1	China	Homo sapiens	2020	F
OM452122.1	China	Homo sapiens	2020	F
OM452124.1	China	Homo sapiens	2020	F
OM452126.1	China	Homo sapiens	2020	F
OM452127.1	China	Homo sapiens	2020	F
OM452128.1	China	Homo sapiens	2020	F
OM452130.1	China	Homo sapiens	2020	F
OM452131.1	China	Homo sapiens	2020	F
OM452132.1	China	Homo sapiens	2020	F
OM452133.1	China	Homo sapiens	2020	F
OM452134.1	China	Homo sapiens	2020	F
OM452139.1	China	Homo sapiens	2020	F
OM452140.1	China	Homo sapiens	2020	F
OM452141.1	China	Homo sapiens	2020	F
OM452142.1	China	Homo sapiens	2020	F
OM452143.1	China	Homo sapiens	2020	F
OM452144.1	China	Homo sapiens	2020	F
OM452145.1	China	Homo sapiens	2020	F
OM452146.1	China	Homo sapiens	2020	F
OM452147.1	China	Homo sapiens	2020	F
OM452148.1	China	Homo sapiens	2020	F
OM452149.1	China	Homo sapiens	2020	F
OM452150.1	China	Homo sapiens	2020	F
OM452151.1	China	Homo sapiens	2020	F
OM452152.1	China	Homo sapiens	2020	F
OM452153.1	China	Homo sapiens	2020	F
OM452154.1	China	Homo sapiens	2020	F
OM452156.1	China	Homo sapiens	2020	F
OM452157.1	China	Homo sapiens	2020	F
OM452159.1	China	Homo sapiens	2020	F
OM452160.1	China	Homo sapiens	2020	F
OM452161.1	China	Homo sapiens	2020	F
OM452162.1	China	Homo sapiens	2020	F
OM452163.1	China	Homo sapiens	2020	F
OM452243.1	China	Homo sapiens	2020	F
MZ773049.1	China	Homo sapiens	2020	F
MZ773050.1	China	Homo sapiens	2020	F
OR797608.1	Korea	Dog	2021	F

LC730375.1	Japan	Homo sapiens	2021	F
OR797607.1	Korea	Dog	2022	F
ON402259.1	China	Homo sapiens	2022	F
ON402260.1	China	Homo sapiens	2022	F
ON402261.1	China	Homo sapiens	2022	F
ON402262.1	China	Homo sapiens	2022	F
ON402263.1	China	Homo sapiens	2022	F
ON402264.1	China	Homo sapiens	2022	F
OR574963.1	China	Homo sapiens	2023	F

Note: 970 S fragments were classified, 952 human sequences, 18 animal sequences.

M Segment Lineage Division

number	country	host	collection year	lineage
OM452267.1	China	Homo sapiens	2012	B
KY273268.1	China	Homo sapiens	2012	B
KY273137.1	Korea	Tick	2013	B
KU507548.1	Korea	Homo sapiens	2013	B
KU507549.1	Korea	Homo sapiens	2013	B
KU507550.1	Korea	Homo sapiens	2013	B
KU507551.1	Korea	Homo sapiens	2013	B
KU507552.1	Korea	Homo sapiens	2013	B
KR698332.1	China	Homo sapiens	2013	B
KR698333.1	China	Homo sapiens	2013	B
KR698334.1	China	Homo sapiens	2013	B
KR698335.1	China	Homo sapiens	2013	B
KR698337.1	China	Homo sapiens	2013	B
KR698338.1	China	Homo sapiens	2013	B
KP280204.1	China	Homo sapiens	2013	B
KF374684.1	China	Homo sapiens	2013	B
NC_018138.1	China	Homo sapiens	2010	F
MZ501602.1	Korea	Goat	2014	B
LC579713.1	Japan	Procyon lotor	2014	B
LC579710.1	Japan	Procyon lotor	2014	B
MT236316.1	China	Tick	2014	B
KY273270.1	China	Homo sapiens	2014	B
KY933691.1	China	Homo sapiens	2014	B
KY933692.1	China	Homo sapiens	2014	B
JQ684872.1	China	Haemaphysalis longicornis	2010	F
HQ419229.1	China	-	2010	F
OR574964.1	China	Homo sapiens	2023	C
HQ419231.1	China	-	2010	F
HQ419232.1	China	-	2010	F
KY933693.1	China	Homo sapiens	2014	B
KY933694.1	China	Homo sapiens	2014	B
KR698342.1	China	Homo sapiens	2014	B
OQ388959.1	China	Homo sapiens	2020	D
OQ388960.1	China	Homo sapiens	2020	A
KR698343.1	China	Apodemus agrarius ningpoensis	2014	B
OQ388962.1	China	Homo sapiens	2021	E
OM452244.1	China	Homo sapiens	2011	F
KR698344.1	China	Apodemus agrarius ningpoensis	2014	B
OM452245.1	China	Homo sapiens	2011	F

MT114247.1	China	Homo sapiens	2011	F
MT005242.1	China	Homo sapiens	2011	F
OQ388968.1	China	Homo sapiens	2022	A
MT005251.1	China	Homo sapiens	2011	F
OQ388970.1	China	Homo sapiens	2022	E
MH491548.1	Korea	Homo sapiens	2015	B
JQ670931.1	China	Homo sapiens	2011	F
KF358692.1	Korea	Homo sapiens	2012	F
OM452250.1	China	Homo sapiens	2012	F
OM452251.1	China	Homo sapiens	2012	F
OM452252.1	China	Homo sapiens	2012	F
MG920820.1	Korea	Homo sapiens	2015	B
OM452253.1	China	Homo sapiens	2012	F
OM452254.1	China	Homo sapiens	2012	F
MG920823.1	Korea	Homo sapiens	2015	B
OM452257.1	China	Homo sapiens	2012	F
OM452259.1	China	Homo sapiens	2012	F
OM452260.1	China	Homo sapiens	2012	F
OM452262.1	China	Homo sapiens	2012	F
OM452264.1	China	Homo sapiens	2012	F
OM452265.1	China	Homo sapiens	2012	F
OM452266.1	China	Homo sapiens	2012	F
MT114249.1	China	Homo sapiens	2012	F
MT114255.1	China	Homo sapiens	2012	F
OQ388990.1	China	Homo sapiens	2022	D
MT005255.1	China	Homo sapiens	2012	F
KC473541.1	China	Haemaphysalis longicornis	2012	F
MG920826.1	Korea	Homo sapiens	2015	B
MG920829.1	Korea	Homo sapiens	2015	B
OQ388995.1	China	Homo sapiens	2020	D
MG921167.1	Korea	Homo sapiens	2015	B
MG921170.1	Korea	Homo sapiens	2015	B
MK513905.1	China	Homo sapiens	2015	B
OM452268.1	China	Homo sapiens	2013	F
OM452270.1	China	Homo sapiens	2013	F
OM452271.1	China	Homo sapiens	2013	F
OM452272.1	China	Homo sapiens	2013	F
OM452273.1	China	Homo sapiens	2013	F
OM452275.1	China	Homo sapiens	2013	F
OM452276.1	China	Homo sapiens	2013	F
OM452277.1	China	Homo sapiens	2013	F
OM452278.1	China	Homo sapiens	2013	F
OM452280.1	China	Homo sapiens	2013	F

OM452246.1	China	Homo sapiens	2011	A
OM452247.1	China	Homo sapiens	2012	A
OM452248.1	China	Homo sapiens	2012	A
OM452249.1	China	Homo sapiens	2012	A
OM452281.1	China	Homo sapiens	2013	F
OM452282.1	China	Homo sapiens	2013	F
OM452284.1	China	Homo sapiens	2013	F
OM452285.1	China	Homo sapiens	2013	F
OM452286.1	China	Homo sapiens	2013	F
OM452255.1	China	Homo sapiens	2012	A
OM452256.1	China	Homo sapiens	2012	A
OM452287.1	China	Homo sapiens	2013	F
OM452258.1	China	Homo sapiens	2012	A
OM452288.1	China	Homo sapiens	2013	F
OM452289.1	China	Homo sapiens	2013	F
OM452261.1	China	Homo sapiens	2012	A
OM452290.1	China	Homo sapiens	2013	F
OM452263.1	China	Homo sapiens	2012	A
OM452291.1	China	Homo sapiens	2013	F
OM452293.1	China	Homo sapiens	2013	F
OM452294.1	China	Homo sapiens	2013	F
MK513914.1	China	Homo sapiens	2015	B
OM452295.1	China	Homo sapiens	2013	F
OM452269.1	China	Homo sapiens	2013	A
OM452297.1	China	Homo sapiens	2013	F
OM452298.1	China	Homo sapiens	2013	F
OM452299.1	China	Homo sapiens	2013	F
OM452302.1	China	Homo sapiens	2013	F
OM452274.1	China	Homo sapiens	2013	A
OM452303.1	China	Homo sapiens	2013	F
OM452304.1	China	Homo sapiens	2013	F
OM452305.1	China	Homo sapiens	2013	F
OM452306.1	China	Homo sapiens	2013	F
OM452279.1	China	Homo sapiens	2013	A
OM452307.1	China	Homo sapiens	2013	F
OM452309.1	China	Homo sapiens	2013	F
OM452310.1	China	Homo sapiens	2013	F
OM452283.1	China	Homo sapiens	2013	A
OM452312.1	China	Homo sapiens	2013	F
OM452315.1	China	Homo sapiens	2013	F
OM452316.1	China	Homo sapiens	2013	F
OM452319.1	China	Homo sapiens	2013	F
OM452321.1	China	Homo sapiens	2013	F

OM452322.1	China	Homo sapiens	2013	F
OM452324.1	China	Homo sapiens	2013	F
OM452325.1	China	Homo sapiens	2013	F
OM452292.1	China	Homo sapiens	2013	A
OM452327.1	China	Homo sapiens	2013	F
OM452328.1	China	Homo sapiens	2013	F
OM452330.1	China	Homo sapiens	2013	F
OM452296.1	China	Homo sapiens	2013	D
OM452333.1	China	Homo sapiens	2013	F
OM452334.1	China	Homo sapiens	2013	F
OM452335.1	China	Homo sapiens	2013	F
OM452300.1	China	Homo sapiens	2013	A
OM452301.1	China	Homo sapiens	2013	A
OM452339.1	China	Homo sapiens	2013	F
OM452341.1	China	Homo sapiens	2013	F
OM452342.1	China	Homo sapiens	2013	F
OM452343.1	China	Homo sapiens	2013	F
OM452344.1	China	Homo sapiens	2013	F
OM452345.1	China	Homo sapiens	2013	F
OM452308.1	China	Homo sapiens	2013	D
OM452346.1	China	Homo sapiens	2013	F
OM452348.1	China	Homo sapiens	2013	F
OM452311.1	China	Homo sapiens	2013	D
OM452350.1	China	Homo sapiens	2013	F
OM452313.1	China	Homo sapiens	2013	A
OM452314.1	China	Homo sapiens	2013	D
OM452351.1	China	Homo sapiens	2013	F
OM452352.1	China	Homo sapiens	2013	F
OM452317.1	China	Homo sapiens	2013	A
OM452318.1	China	Homo sapiens	2013	A
OM452355.1	China	Homo sapiens	2013	F
OM452320.1	China	Homo sapiens	2013	A
OM452356.1	China	Homo sapiens	2013	F
OM452357.1	China	Homo sapiens	2013	F
OM452323.1	China	Homo sapiens	2013	A
OM452358.1	China	Homo sapiens	2013	F
OM452359.1	China	Homo sapiens	2013	F
OM452326.1	China	Homo sapiens	2013	A
OM452360.1	China	Homo sapiens	2013	F
OM452361.1	China	Homo sapiens	2013	F
OM452329.1	China	Homo sapiens	2013	A
OM452365.1	China	Homo sapiens	2013	F
OM452331.1	China	Homo sapiens	2013	A

OM452332.1	China	Homo sapiens	2013	D
MT005260.1	China	Homo sapiens	2013	F
MT005268.1	China	Homo sapiens	2013	F
KU361342.1	China	Homo sapiens	2013	F
OM452336.1	China	Homo sapiens	2013	D
OM452337.1	China	Homo sapiens	2013	A
OM452338.1	China	Homo sapiens	2013	D
NC_043451.1	China	Homo sapiens	2014	F
OM452340.1	China	Homo sapiens	2013	A
OM452367.1	China	Homo sapiens	2014	F
OM452369.1	China	Homo sapiens	2014	F
OM452370.1	China	Homo sapiens	2014	F
OM452372.1	China	Homo sapiens	2014	F
OM452374.1	China	Homo sapiens	2014	F
OM452375.1	China	Homo sapiens	2014	F
OM452347.1	China	Homo sapiens	2013	A
OM452376.1	China	Homo sapiens	2014	F
OM452349.1	China	Homo sapiens	2013	A
OM452378.1	China	Homo sapiens	2014	F
OM452379.1	China	Homo sapiens	2014	F
OM452382.1	China	Homo sapiens	2014	F
OM452353.1	China	Homo sapiens	2013	A
OM452354.1	China	Homo sapiens	2013	A
OM452383.1	China	Homo sapiens	2014	F
OM452384.1	China	Homo sapiens	2014	F
OM452385.1	China	Homo sapiens	2014	F
OM452387.1	China	Homo sapiens	2014	F
OM452388.1	China	Homo sapiens	2014	F
OM452390.1	China	Homo sapiens	2014	F
OM452391.1	China	Homo sapiens	2014	A
OM452362.1	China	Homo sapiens	2013	A
OM452363.1	China	Homo sapiens	2013	A
OM452364.1	China	Homo sapiens	2013	A
OM452392.1	China	Homo sapiens	2014	F
OM452366.1	China	Homo sapiens	2013	A
OM452393.1	China	Homo sapiens	2014	F
OM452368.1	China	Homo sapiens	2014	A
OM452395.1	China	Homo sapiens	2014	F
OM452397.1	China	Homo sapiens	2014	F
OM452371.1	China	Homo sapiens	2014	A
OM452398.1	China	Homo sapiens	2014	F
OM452373.1	China	Homo sapiens	2014	D
OM452401.1	China	Homo sapiens	2014	F

OM452403.1	China	Homo sapiens	2014	F
OM452405.1	China	Homo sapiens	2014	F
OM452377.1	China	Homo sapiens	2014	A
OM452407.1	China	Homo sapiens	2014	F
OM452409.1	China	Homo sapiens	2014	F
OM452380.1	China	Homo sapiens	2014	A
OM452381.1	China	Homo sapiens	2014	A
OM452410.1	China	Homo sapiens	2014	F
OM452411.1	China	Homo sapiens	2014	F
OM452412.1	China	Homo sapiens	2014	F
OM452413.1	China	Homo sapiens	2014	F
OM452386.1	China	Homo sapiens	2014	A
OM452414.1	China	Homo sapiens	2014	F
OM452416.1	China	Homo sapiens	2014	F
OM452389.1	China	Homo sapiens	2014	A
OM452418.1	China	Homo sapiens	2014	F
OM452419.1	China	Homo sapiens	2014	F
OM452420.1	China	Homo sapiens	2014	F
OM452422.1	China	Homo sapiens	2014	F
OM452394.1	China	Homo sapiens	2014	D
OM452423.1	China	Homo sapiens	2014	F
OM452396.1	China	Homo sapiens	2014	D
OM452424.1	China	Homo sapiens	2014	F
OM452425.1	China	Homo sapiens	2014	F
OM452399.1	China	Homo sapiens	2014	A
OM452400.1	China	Homo sapiens	2014	A
OM452427.1	China	Homo sapiens	2014	F
OM452402.1	China	Homo sapiens	2014	A
OM452432.1	China	Homo sapiens	2014	F
OM452404.1	China	Homo sapiens	2014	A
OM452433.1	China	Homo sapiens	2014	F
OM452406.1	China	Homo sapiens	2014	D
OM452438.1	China	Homo sapiens	2014	F
OM452408.1	China	Homo sapiens	2014	A
OM452439.1	China	Homo sapiens	2014	F
OM452440.1	China	Homo sapiens	2014	F
OM452441.1	China	Homo sapiens	2014	F
OM452442.1	China	Homo sapiens	2014	F
OM452443.1	China	Homo sapiens	2014	F
OM452446.1	China	Homo sapiens	2014	F
OM452415.1	China	Homo sapiens	2014	A
OM452447.1	China	Homo sapiens	2014	F
OM452417.1	China	Homo sapiens	2014	D

OM452448.1	China	Homo sapiens	2014	F
OM452450.1	China	Homo sapiens	2014	F
OM452451.1	China	Homo sapiens	2014	F
OM452421.1	China	Homo sapiens	2014	A
OM452453.1	China	Homo sapiens	2014	F
OM452454.1	China	Homo sapiens	2014	F
OM452455.1	China	Homo sapiens	2014	F
OM452456.1	China	Homo sapiens	2014	F
OM452426.1	China	Homo sapiens	2014	A
OM452457.1	China	Homo sapiens	2014	F
OM452428.1	China	Homo sapiens	2014	A
OM452429.1	China	Homo sapiens	2014	A
OM452430.1	China	Homo sapiens	2014	D
OM452431.1	China	Homo sapiens	2014	D
OM452458.1	China	Homo sapiens	2014	F
OM452459.1	China	Homo sapiens	2014	F
OM452434.1	China	Homo sapiens	2014	A
OM452435.1	China	Homo sapiens	2014	A
OM452436.1	China	Homo sapiens	2014	A
OM452437.1	China	Homo sapiens	2014	D
OM452460.1	China	Homo sapiens	2014	F
OM452461.1	China	Homo sapiens	2014	F
OM452463.1	China	Homo sapiens	2014	F
OM452464.1	China	Homo sapiens	2014	F
OM452465.1	China	Homo sapiens	2014	F
OM452466.1	China	Homo sapiens	2014	F
OM452444.1	China	Homo sapiens	2014	D
OM452445.1	China	Homo sapiens	2014	A
OM452467.1	China	Homo sapiens	2014	F
OM452469.1	China	Homo sapiens	2014	F
OM452470.1	China	Homo sapiens	2014	F
OM452449.1	China	Homo sapiens	2014	A
OM452471.1	China	Homo sapiens	2014	F
OM452473.1	China	Homo sapiens	2014	F
OM452452.1	China	Homo sapiens	2014	D
OM452474.1	China	Homo sapiens	2014	F
MT114259.1	China	Homo sapiens	2014	F
MT005272.1	China	Homo sapiens	2014	F
MT005275.1	China	Homo sapiens	2014	F
KY965128.1	China	Homo sapiens	2014	F
KY933684.1	China	Homo sapiens	2014	F
KR706566.1	China	Homo sapiens	2014	F
KR080475.1	China	Homo sapiens	2014	F

OM452476.1	China	Homo sapiens	2015	F
OM452462.1	China	Homo sapiens	2014	A
OM452477.1	China	Homo sapiens	2015	F
OM452478.1	China	Homo sapiens	2015	F
OM452479.1	China	Homo sapiens	2015	F
OM452481.1	China	Homo sapiens	2015	F
OM452482.1	China	Homo sapiens	2015	F
OM452468.1	China	Homo sapiens	2014	A
OM452486.1	China	Homo sapiens	2015	F
OM452487.1	China	Homo sapiens	2015	F
OM452488.1	China	Homo sapiens	2015	F
OM452472.1	China	Homo sapiens	2014	A
OM452489.1	China	Homo sapiens	2015	F
OM452492.1	China	Homo sapiens	2015	F
OM452475.1	China	Homo sapiens	2016	D
OM452495.1	China	Homo sapiens	2015	F
OM452496.1	China	Homo sapiens	2015	F
OM452497.1	China	Homo sapiens	2015	F
OM452498.1	China	Homo sapiens	2015	F
OM452480.1	China	Homo sapiens	2015	D
OM452500.1	China	Homo sapiens	2015	F
OM452501.1	China	Homo sapiens	2015	F
OM452503.1	China	Homo sapiens	2015	F
OM452484.1	China	Homo sapiens	2015	A
OM452485.1	China	Homo sapiens	2015	A
OM452505.1	China	Homo sapiens	2015	F
OM452507.1	China	Homo sapiens	2015	F
OM452508.1	China	Homo sapiens	2015	F
OM452510.1	China	Homo sapiens	2015	F
OM452490.1	China	Homo sapiens	2015	A
OM452491.1	China	Homo sapiens	2015	D
OM452511.1	China	Homo sapiens	2015	F
OM452493.1	China	Homo sapiens	2015	A
OM452494.1	China	Homo sapiens	2015	A
OM452512.1	China	Homo sapiens	2015	F
OM452513.1	China	Homo sapiens	2015	F
OM452514.1	China	Homo sapiens	2015	F
OM452515.1	China	Homo sapiens	2015	F
OM452499.1	China	Homo sapiens	2015	A
OM452516.1	China	Homo sapiens	2015	F
OM452517.1	China	Homo sapiens	2015	F
OM452502.1	China	Homo sapiens	2015	A
OM452519.1	China	Homo sapiens	2015	F

OM452504.1	China	Homo sapiens	2015	D
OM452520.1	China	Homo sapiens	2015	F
OM452506.1	China	Homo sapiens	2015	D
OM452521.1	China	Homo sapiens	2015	F
OM452522.1	China	Homo sapiens	2015	F
OM452509.1	China	Homo sapiens	2015	A
OM452523.1	China	Homo sapiens	2015	F
OM452524.1	China	Homo sapiens	2015	F
OM452525.1	China	Homo sapiens	2015	F
OM452526.1	China	Homo sapiens	2015	F
OM452528.1	China	Homo sapiens	2015	F
OM452529.1	China	Homo sapiens	2015	F
OM452531.1	China	Homo sapiens	2015	F
OM452533.1	China	Homo sapiens	2015	F
OM452518.1	China	Homo sapiens	2015	D
OM452534.1	China	Homo sapiens	2015	F
OM452535.1	China	Homo sapiens	2015	F
OM452536.1	China	Homo sapiens	2015	F
OM452537.1	China	Homo sapiens	2015	F
OM452538.1	China	Homo sapiens	2015	F
OM452539.1	China	Homo sapiens	2015	F
OM452541.1	China	Homo sapiens	2015	F
OM452542.1	China	Homo sapiens	2015	F
OM452527.1	China	Homo sapiens	2015	A
OM452543.1	China	Homo sapiens	2015	F
OM452544.1	China	Homo sapiens	2015	F
OM452530.1	China	Homo sapiens	2016	D
OM452545.1	China	Homo sapiens	2015	F
OM452532.1	China	Homo sapiens	2015	A
OM452546.1	China	Homo sapiens	2015	F
OM452548.1	China	Homo sapiens	2015	F
OM452549.1	China	Homo sapiens	2015	F
OM452550.1	China	Homo sapiens	2015	F
OM452551.1	China	Homo sapiens	2015	F
OM452553.1	China	Homo sapiens	2015	F
MK513908.1	China	Homo sapiens	2015	F
OM452540.1	China	Homo sapiens	2015	A
MK513917.1	China	Homo sapiens	2015	F
MK513920.1	China	Homo sapiens	2015	F
MK513929.1	China	Homo sapiens	2015	F
MF140448.1	China	Rat	2015	F
KY933683.1	China	Homo sapiens	2015	F
OM452483.1	China	Homo sapiens	2016	F

OM452547.1	China	Homo sapiens	2015	D
OM452555.1	China	Homo sapiens	2016	F
OM452556.1	China	Homo sapiens	2016	F
OM452557.1	China	Homo sapiens	2016	F
OM452558.1	China	Homo sapiens	2016	F
OM452552.1	China	Homo sapiens	2015	A
OM452560.1	China	Homo sapiens	2016	F
OM452554.1	China	Homo sapiens	2016	A
OM452563.1	China	Homo sapiens	2016	F
OM452564.1	China	Homo sapiens	2016	F
OM452565.1	China	Homo sapiens	2016	F
OM452567.1	China	Homo sapiens	2016	F
OM452559.1	China	Homo sapiens	2016	A
OM452569.1	China	Homo sapiens	2016	F
OM452561.1	China	Homo sapiens	2016	A
OM452562.1	China	Homo sapiens	2016	A
OM452570.1	China	Homo sapiens	2016	F
OM452572.1	China	Homo sapiens	2016	F
OM452573.1	China	Homo sapiens	2016	F
OM452566.1	China	Homo sapiens	2016	A
OM452574.1	China	Homo sapiens	2016	F
OM452568.1	China	Homo sapiens	2016	A
OM452575.1	China	Homo sapiens	2016	F
OM452577.1	China	Homo sapiens	2016	F
OM452571.1	China	Homo sapiens	2016	A
OM452578.1	China	Homo sapiens	2016	F
OM452580.1	China	Homo sapiens	2016	F
OM452585.1	China	Homo sapiens	2016	F
OM452587.1	China	Homo sapiens	2016	F
OM452576.1	China	Homo sapiens	2016	A
OM452590.1	China	Homo sapiens	2016	F
OM452591.1	China	Homo sapiens	2016	F
OM452579.1	China	Homo sapiens	2016	A
OM452592.1	China	Homo sapiens	2016	F
OM452581.1	China	Homo sapiens	2016	A
OM452582.1	China	Homo sapiens	2016	A
OM452583.1	China	Homo sapiens	2016	A
OM452584.1	China	Homo sapiens	2016	A
KY773991.1	China	Haemaphysalis longicornis	2016	F
OM452586.1	China	Homo sapiens	2016	A
KY965096.1	China	Homo sapiens	2016	F
OM452588.1	China	Homo sapiens	2016	A
OM452589.1	China	Homo sapiens	2016	D

KY965097.1	China	Homo sapiens	2016	F
KY965099.1	China	Homo sapiens	2016	F
KY965101.1	China	Homo sapiens	2016	F
OM452593.1	China	Homo sapiens	2016	A
OM452594.1	China	Homo sapiens	2016	D
OM452595.1	China	Homo sapiens	2017	A
KY965102.1	China	Homo sapiens	2016	F
KY965104.1	China	Homo sapiens	2016	F
OM452598.1	China	Homo sapiens	2017	A
KY965107.1	China	Homo sapiens	2016	F
OM452600.1	China	Homo sapiens	2017	A
OM452601.1	China	Homo sapiens	2017	A
KY965108.1	China	Homo sapiens	2016	F
KY965127.1	China	Homo sapiens	2016	F
OM452604.1	China	Homo sapiens	2018	A
KY440773.1	China	Homo sapiens	2016	F
OM452596.1	China	Homo sapiens	2017	F
OM452607.1	China	Homo sapiens	2018	A
OM452597.1	China	Homo sapiens	2017	F
OM452599.1	China	Homo sapiens	2017	F
OM452610.1	China	Homo sapiens	2018	D
OM452611.1	China	Homo sapiens	2018	A
OM452602.1	China	Homo sapiens	2017	F
OM452613.1	China	Homo sapiens	2018	D
OM452603.1	China	Homo sapiens	2017	F
OM452615.1	China	Homo sapiens	2018	A
OM452605.1	China	Homo sapiens	2017	F
OM452608.1	China	Homo sapiens	2017	F
OM452618.1	China	Homo sapiens	2017	A
OM452609.1	China	Homo sapiens	2017	F
OM452612.1	China	Homo sapiens	2017	F
OM452614.1	China	Homo sapiens	2017	F
OM452616.1	China	Homo sapiens	2017	F
OM452619.1	China	Homo sapiens	2017	F
OM452623.1	China	Homo sapiens	2017	F
OM452624.1	China	Homo sapiens	2017	F
OM452625.1	China	Homo sapiens	2017	F
OM452635.1	China	Homo sapiens	2017	F
OM452628.1	China	Homo sapiens	2018	A
OM452640.1	China	Homo sapiens	2017	F
OM452630.1	China	Homo sapiens	2018	A
OM452631.1	China	Homo sapiens	2018	A
OM452667.1	China	Homo sapiens	2017	F

OM452670.1	China	Homo sapiens	2017	F
OM452674.1	China	Homo sapiens	2017	F
OM452681.1	China	Homo sapiens	2017	F
OM452682.1	China	Homo sapiens	2017	F
OM452637.1	China	Homo sapiens	2017	D
OM452683.1	China	Homo sapiens	2017	F
OM452685.1	China	Homo sapiens	2017	F
OM452687.1	China	Homo sapiens	2017	F
OM452693.1	China	Homo sapiens	2017	F
OM452694.1	China	Homo sapiens	2017	F
OM452643.1	China	Homo sapiens	2018	D
OM452696.1	China	Homo sapiens	2017	F
OM452645.1	China	Homo sapiens	2017	D
OM452701.1	China	Homo sapiens	2017	F
OM452704.1	China	Homo sapiens	2017	F
OM452648.1	China	Homo sapiens	2017	A
OM452706.1	China	Homo sapiens	2017	F
OM452650.1	China	Homo sapiens	2018	D
OM452651.1	China	Homo sapiens	2018	A
OM452713.1	China	Homo sapiens	2017	F
OM452724.1	China	Homo sapiens	2017	F
OM452729.1	China	Homo sapiens	2017	F
OM452733.1	China	Homo sapiens	2017	F
OM452734.1	China	Homo sapiens	2017	F
OM452657.1	China	Homo sapiens	2018	A
OM452742.1	China	Homo sapiens	2017	F
OM452743.1	China	Homo sapiens	2017	F
MT320803.1	China	Homo sapiens	2017	F
MT320806.1	China	Homo sapiens	2017	F
OM452662.1	China	Homo sapiens	2018	D
OM452663.1	China	Homo sapiens	2017	A
OM452664.1	China	Homo sapiens	2018	A
MT320809.1	China	Homo sapiens	2017	F
MT320812.1	China	Homo sapiens	2017	F
MK524355.1	China	Homo sapiens	2017	F
MK524361.1	China	Homo sapiens	2017	F
MK524367.1	China	Homo sapiens	2017	F
MK524373.1	China	Homo sapiens	2017	F
MK300946.1	China	Homo sapiens	2017	F
OM452672.1	China	Homo sapiens	2017	A
OM452673.1	China	Homo sapiens	2018	D
OP899817.1	China	Erinaceus amurensis	2018	F
OM452606.1	China	Homo sapiens	2018	F

OM452617.1	China	Homo sapiens	2018	F
OM452620.1	China	Homo sapiens	2018	F
OM452678.1	China	Homo sapiens	2018	A
OM452621.1	China	Homo sapiens	2018	F
OM452680.1	China	Homo sapiens	2018	A
OM452622.1	China	Homo sapiens	2018	F
OM452626.1	China	Homo sapiens	2018	F
OM452627.1	China	Homo sapiens	2018	F
OM452684.1	China	Homo sapiens	2018	A
OM452629.1	China	Homo sapiens	2018	F
OM452686.1	China	Homo sapiens	2018	A
OM452632.1	China	Homo sapiens	2018	F
OM452688.1	China	Homo sapiens	2017	A
OM452689.1	China	Homo sapiens	2017	A
OM452633.1	China	Homo sapiens	2018	F
OM452634.1	China	Homo sapiens	2018	F
OM452692.1	China	Homo sapiens	2017	D
OM452636.1	China	Homo sapiens	2018	F
OM452638.1	China	Homo sapiens	2018	F
OM452639.1	China	Homo sapiens	2018	F
OM452641.1	China	Homo sapiens	2018	F
OM452642.1	China	Homo sapiens	2018	F
OM452644.1	China	Homo sapiens	2018	F
OM452646.1	China	Homo sapiens	2018	F
OM452647.1	China	Homo sapiens	2018	F
OM452649.1	China	Homo sapiens	2018	F
OM452652.1	China	Homo sapiens	2018	F
OM452653.1	China	Homo sapiens	2018	F
OM452654.1	China	Homo sapiens	2018	F
OM452655.1	China	Homo sapiens	2018	F
OM452656.1	China	Homo sapiens	2018	F
OM452658.1	China	Homo sapiens	2018	F
OM452708.1	China	Homo sapiens	2018	D
OM452659.1	China	Homo sapiens	2018	F
OM452660.1	China	Homo sapiens	2018	F
OM452661.1	China	Homo sapiens	2018	F
OM452665.1	China	Homo sapiens	2018	F
OM452666.1	China	Homo sapiens	2018	F
OM452668.1	China	Homo sapiens	2018	F
OM452669.1	China	Homo sapiens	2018	F
OM452671.1	China	Homo sapiens	2018	F
OM452717.1	China	Homo sapiens	2017	A
OM452718.1	China	Homo sapiens	2018	A

OM452719.1	China	Homo sapiens	2018	A
OM452720.1	China	Homo sapiens	2017	A
OM452721.1	China	Homo sapiens	2018	A
OM452675.1	China	Homo sapiens	2018	F
OM452676.1	China	Homo sapiens	2018	F
OM452677.1	China	Homo sapiens	2018	F
OM452679.1	China	Homo sapiens	2018	F
OM452690.1	China	Homo sapiens	2018	F
OM452691.1	China	Homo sapiens	2018	F
OM452695.1	China	Homo sapiens	2018	F
OM452697.1	China	Homo sapiens	2018	F
OM452730.1	China	Homo sapiens	2018	A
OM452698.1	China	Homo sapiens	2018	F
OM452699.1	China	Homo sapiens	2018	F
OM452700.1	China	Homo sapiens	2018	F
OM452702.1	China	Homo sapiens	2018	F
OM452703.1	China	Homo sapiens	2018	F
OM452705.1	China	Homo sapiens	2018	F
OM452707.1	China	Homo sapiens	2018	F
OM452738.1	China	Homo sapiens	2017	A
OM452739.1	China	Homo sapiens	2018	D
OM452740.1	China	Homo sapiens	2018	A
OM452709.1	China	Homo sapiens	2018	F
OM452710.1	China	Homo sapiens	2018	F
OM452711.1	China	Homo sapiens	2018	F
OM452712.1	China	Homo sapiens	2018	F
OM452714.1	China	Homo sapiens	2018	F
OM452746.1	China	Homo sapiens	2019	D
OM452715.1	China	Homo sapiens	2018	F
OM452716.1	China	Homo sapiens	2018	F
OM452749.1	China	Homo sapiens	2019	D
OM452722.1	China	Homo sapiens	2018	F
OM452723.1	China	Homo sapiens	2018	F
OM452752.1	China	Homo sapiens	2019	D
OM452753.1	China	Homo sapiens	2019	A
OM452725.1	China	Homo sapiens	2018	F
OM452726.1	China	Homo sapiens	2018	F
OM452727.1	China	Homo sapiens	2018	F
OM452757.1	China	Homo sapiens	2019	A
OM452758.1	China	Homo sapiens	2019	A
OM452728.1	China	Homo sapiens	2018	F
OM452731.1	China	Homo sapiens	2018	F
OM452732.1	China	Homo sapiens	2018	F

OM452735.1	China	Homo sapiens	2018	F
OM452736.1	China	Homo sapiens	2018	F
OM452737.1	China	Homo sapiens	2018	F
OM452741.1	China	Homo sapiens	2018	F
OQ388955.1	China	Homo sapiens	2019	F
OM452744.1	China	Homo sapiens	2019	F
OM452768.1	China	Homo sapiens	2020	A
OM452745.1	China	Homo sapiens	2019	F
OM452747.1	China	Homo sapiens	2019	F
OM452748.1	China	Homo sapiens	2019	F
OM452750.1	China	Homo sapiens	2019	F
OM452751.1	China	Homo sapiens	2019	F
OM452754.1	China	Homo sapiens	2019	F
OM452755.1	China	Homo sapiens	2019	F
OM452756.1	China	Homo sapiens	2019	F
OM452759.1	China	Homo sapiens	2019	F
OM452760.1	China	Homo sapiens	2019	F
OM452779.1	China	Homo sapiens	2020	A
OM452761.1	China	Homo sapiens	2019	F
OM452762.1	China	Homo sapiens	2019	F
OM452763.1	China	Homo sapiens	2019	F
OM452764.1	China	Homo sapiens	2019	F
OM452765.1	China	Homo sapiens	2019	F
OM452766.1	China	Homo sapiens	2019	F
OM452866.1	China	Homo sapiens	2019	F
MH464251.1	Korea	Dog	2016	B
OM452867.1	China	Homo sapiens	2019	F
OM452789.1	China	Homo sapiens	2020	A
OM452868.1	China	Homo sapiens	2019	F
OM452791.1	China	Homo sapiens	2020	A
OM452792.1	China	Homo sapiens	2020	A
OM452793.1	China	Homo sapiens	2020	D
OM452869.1	China	Homo sapiens	2019	F
OM452870.1	China	Homo sapiens	2019	F
OM452796.1	China	Homo sapiens	2020	A
OM452872.1	China	Homo sapiens	2019	F
OM452798.1	China	Homo sapiens	2020	A
OM452873.1	China	Homo sapiens	2019	F
OM452875.1	China	Homo sapiens	2019	F
OM452801.1	China	Homo sapiens	2020	A
OM452802.1	China	Homo sapiens	2020	A
OM452803.1	China	Homo sapiens	2020	A
OM452876.1	China	Homo sapiens	2019	F

OM452877.1	China	Homo sapiens	2019	F
OM452879.1	China	Homo sapiens	2019	F
OM452880.1	China	Homo sapiens	2019	F
OM452808.1	China	Homo sapiens	2020	A
OM452881.1	China	Homo sapiens	2019	F
OM452882.1	China	Homo sapiens	2019	F
OM452883.1	China	Homo sapiens	2019	F
OM452812.1	China	Homo sapiens	2020	A
OM452884.1	China	Homo sapiens	2019	F
OM452885.1	China	Homo sapiens	2019	F
OM452815.1	China	Homo sapiens	2020	A
OM452886.1	China	Homo sapiens	2019	F
OM452817.1	China	Homo sapiens	2020	A
OM452887.1	China	Homo sapiens	2019	F
OM452819.1	China	Homo sapiens	2020	D
OM452888.1	China	Homo sapiens	2019	F
OM452890.1	China	Homo sapiens	2019	F
OM452822.1	China	Homo sapiens	2020	A
OM452893.1	China	Homo sapiens	2019	F
OM452824.1	China	Homo sapiens	2020	A
OM452825.1	China	Homo sapiens	2020	A
OM452894.1	China	Homo sapiens	2019	F
OM452895.1	China	Homo sapiens	2019	F
OM452828.1	China	Homo sapiens	2020	A
OM452896.1	China	Homo sapiens	2019	F
OM452897.1	China	Homo sapiens	2019	F
OM452898.1	China	Homo sapiens	2019	F
OM452899.1	China	Homo sapiens	2019	F
OM452900.1	China	Homo sapiens	2019	F
OM452834.1	China	Homo sapiens	2020	A
OM452835.1	China	Homo sapiens	2020	D
OM452901.1	China	Homo sapiens	2019	F
OM452837.1	China	Homo sapiens	2020	D
OM452902.1	China	Homo sapiens	2019	F
OM452903.1	China	Homo sapiens	2019	F
OM452904.1	China	Homo sapiens	2019	F
OM452905.1	China	Homo sapiens	2019	F
OM452906.1	China	Homo sapiens	2019	F
OM452907.1	China	Homo sapiens	2019	F
OM452908.1	China	Homo sapiens	2019	F
OM452909.1	China	Homo sapiens	2019	F
OM452910.1	China	Homo sapiens	2019	F
OM452911.1	China	Homo sapiens	2019	F

OM452912.1	China	Homo sapiens	2019	F
OM452914.1	China	Homo sapiens	2019	F
OM452915.1	China	Homo sapiens	2019	F
OM452917.1	China	Homo sapiens	2019	F
OM452919.1	China	Homo sapiens	2019	F
OM452920.1	China	Homo sapiens	2019	F
OM452854.1	China	Homo sapiens	2020	D
OM452921.1	China	Homo sapiens	2019	F
OM452922.1	China	Homo sapiens	2019	F
OM452857.1	China	Homo sapiens	2020	A
OM452923.1	China	Homo sapiens	2019	F
OM452924.1	China	Homo sapiens	2019	F
OM452925.1	China	Homo sapiens	2019	F
OM452926.1	China	Homo sapiens	2019	F
OM452927.1	China	Homo sapiens	2019	F
OM452863.1	China	Homo sapiens	2020	A
OM452864.1	China	Homo sapiens	2019	D
OM452865.1	China	Homo sapiens	2019	A
OM452929.1	China	Homo sapiens	2019	F
OM452930.1	China	Homo sapiens	2019	F
OM452932.1	China	Homo sapiens	2019	F
OM452933.1	China	Homo sapiens	2019	F
OM452936.1	China	Homo sapiens	2019	F
OM452871.1	China	Homo sapiens	2019	A
OM452937.1	China	Homo sapiens	2019	F
OM452938.1	China	Homo sapiens	2019	F
OM452874.1	China	Homo sapiens	2019	A
OM452939.1	China	Homo sapiens	2019	F
LC473509.1	Japan	Felis catus	2019	F
OQ388999.1	China	Homo sapiens	2020	F
OM452878.1	China	Homo sapiens	2019	A
OM452767.1	China	Homo sapiens	2020	F
OM452769.1	China	Homo sapiens	2020	F
OM452770.1	China	Homo sapiens	2020	F
OM452771.1	China	Homo sapiens	2020	F
OM452772.1	China	Homo sapiens	2020	F
OM452773.1	China	Homo sapiens	2020	F
OM452774.1	China	Homo sapiens	2020	F
OM452775.1	China	Homo sapiens	2020	F
OM452776.1	China	Homo sapiens	2020	F
OM452777.1	China	Homo sapiens	2020	F
OM452889.1	China	Homo sapiens	2019	A
OM452778.1	China	Homo sapiens	2020	F

OM452891.1	China	Homo sapiens	2019	A
OM452892.1	China	Homo sapiens	2019	A
OM452780.1	China	Homo sapiens	2020	F
OM452781.1	China	Homo sapiens	2020	F
OM452782.1	China	Homo sapiens	2020	F
OM452783.1	China	Homo sapiens	2020	F
OM452784.1	China	Homo sapiens	2020	F
OM452785.1	China	Homo sapiens	2020	F
OM452786.1	China	Homo sapiens	2020	F
OM452788.1	China	Homo sapiens	2020	F
OM452790.1	China	Homo sapiens	2020	F
OM452794.1	China	Homo sapiens	2020	F
OM452795.1	China	Homo sapiens	2020	F
OM452797.1	China	Homo sapiens	2020	F
OM452799.1	China	Homo sapiens	2020	F
OM452800.1	China	Homo sapiens	2020	F
OM452804.1	China	Homo sapiens	2020	F
OM452805.1	China	Homo sapiens	2020	F
OM452806.1	China	Homo sapiens	2020	F
OM452807.1	China	Homo sapiens	2020	F
OM452809.1	China	Homo sapiens	2020	F
OM452810.1	China	Homo sapiens	2020	F
OM452913.1	China	Homo sapiens	2019	D
OM452811.1	China	Homo sapiens	2020	F
OM452813.1	China	Homo sapiens	2020	F
OM452916.1	China	Homo sapiens	2019	A
OM452814.1	China	Homo sapiens	2020	F
OM452918.1	China	Homo sapiens	2019	D
OM452816.1	China	Homo sapiens	2020	F
OM452818.1	China	Homo sapiens	2020	F
OM452820.1	China	Homo sapiens	2020	F
OM452821.1	China	Homo sapiens	2020	F
OM452823.1	China	Homo sapiens	2020	F
OM452826.1	China	Homo sapiens	2020	F
OM452827.1	China	Homo sapiens	2020	F
OM452829.1	China	Homo sapiens	2020	F
OM452830.1	China	Homo sapiens	2020	F
OM452928.1	China	Homo sapiens	2019	A
OM452831.1	China	Homo sapiens	2020	F
OM452832.1	China	Homo sapiens	2020	F
OM452931.1	China	Homo sapiens	2019	A
OM452833.1	China	Homo sapiens	2020	F
OM452836.1	China	Homo sapiens	2020	F

OM452934.1	China	Homo sapiens	2019	D
OM452935.1	China	Homo sapiens	2019	D
OM452838.1	China	Homo sapiens	2020	F
OM452839.1	China	Homo sapiens	2020	F
OM452840.1	China	Homo sapiens	2020	F
OM452841.1	China	Homo sapiens	2020	F
OM452842.1	China	Homo sapiens	2020	F
OM452941.1	China	Homo sapiens	2020	D
OM452942.1	China	Homo sapiens	2020	D
MZ773030.1	China	Homo sapiens	2020	A
MZ773031.1	China	Homo sapiens	2020	A
MZ773032.1	China	Homo sapiens	2020	A
MZ773033.1	China	Homo sapiens	2020	A
LC536546.1	Japan	Haemaphysalis formosensis	2016	B
LC536547.1	Japan	Amblyomma testudinarium	2016	B
MZ773036.1	China	Homo sapiens	2020	A
MZ773037.1	China	Homo sapiens	2020	A
MZ773038.1	China	Homo sapiens	2020	A
MZ773039.1	China	Homo sapiens	2020	A
MZ773040.1	China	Homo sapiens	2020	A
MZ773041.1	China	Homo sapiens	2020	A
MZ773042.1	China	Homo sapiens	2020	A
LC536548.1	Japan	Amblyomma testudinarium	2016	B
LC536549.1	Japan	Haemaphysalis flava	2016	B
MW721875.1	China	Haemaphysalis longicornis	2019	D
MW721876.1	China	Haemaphysalis longicornis	2019	D
MZ965011.1	China	Haemaphysalis longicornis	2019	D
MZ965012.1	China	Haemaphysalis longicornis	2019	D
MT114246.1	China	Homo sapiens	2011	D
OM452843.1	China	Homo sapiens	2020	F
MT114248.1	China	Homo sapiens	2011	E
OM452844.1	China	Homo sapiens	2020	F
MT114250.1	China	Homo sapiens	2012	A
MT114251.1	China	Homo sapiens	2011	D
MT114252.1	China	Homo sapiens	2012	D
MT114253.1	China	Homo sapiens	2012	A
MT114254.1	China	Homo sapiens	2012	A
OM452845.1	China	Homo sapiens	2020	F
MT114256.1	China	Homo sapiens	2012	C
MT114257.1	China	Homo sapiens	2013	A
MT114258.1	China	Homo sapiens	2013	D
OM452846.1	China	Homo sapiens	2020	F
MT320788.1	China	Homo sapiens	2017	D

MT320791.1	China	Homo sapiens	2017	D
MT320794.1	China	Homo sapiens	2017	D
MT320797.1	China	Homo sapiens	2017	A
MT320800.1	China	Homo sapiens	2017	A
OM452847.1	China	Homo sapiens	2020	F
OM452848.1	China	Homo sapiens	2020	F
OM452849.1	China	Homo sapiens	2020	F
OM452850.1	China	Homo sapiens	2020	F
MT320815.1	China	Homo sapiens	2017	D
MT320818.1	China	Homo sapiens	2017	D
OM452851.1	China	Homo sapiens	2020	F
MT005243.1	China	Homo sapiens	2011	D
MT005244.1	China	Homo sapiens	2011	A
MT005245.1	China	Homo sapiens	2011	D
MT005246.1	China	Homo sapiens	2011	D
MT005247.1	China	Homo sapiens	2011	D
MT005248.1	China	Homo sapiens	2011	D
MT005249.1	China	Homo sapiens	2011	D
MT005250.1	China	Homo sapiens	2011	D
OM452852.1	China	Homo sapiens	2020	F
MT005252.1	China	Homo sapiens	2011	D
MT005253.1	China	Homo sapiens	2011	D
MT005254.1	China	Homo sapiens	2011	D
OM452853.1	China	Homo sapiens	2020	F
MT005256.1	China	Homo sapiens	2012	D
MT005257.1	China	Homo sapiens	2012	D
MT005258.1	China	Homo sapiens	2012	D
MT005259.1	China	Homo sapiens	2012	A
OM452855.1	China	Homo sapiens	2020	F
MT005261.1	China	Homo sapiens	2013	A
MT005262.1	China	Homo sapiens	2013	A
MT005263.1	China	Homo sapiens	2013	D
MT005264.1	China	Homo sapiens	2013	D
MT005265.1	China	Homo sapiens	2013	A
MT005266.1	China	Homo sapiens	2013	D
MT005267.1	China	Homo sapiens	2013	D
OM452856.1	China	Homo sapiens	2020	F
MT005269.1	China	Homo sapiens	2013	D
MT005270.1	China	Homo sapiens	2013	D
MT005271.1	China	Homo sapiens	2014	D
OM452858.1	China	Homo sapiens	2020	F
MT005273.1	China	Homo sapiens	2014	D
MT005274.1	China	Homo sapiens	2014	D

OM452859.1	China	Homo sapiens	2020	F
MT005276.1	China	Homo sapiens	2014	D
MT005277.1	China	Homo sapiens	2014	D
MT005278.1	China	Homo sapiens	2014	D
MT005279.1	China	Homo sapiens	2015	D
MT005280.1	China	Homo sapiens	2015	D
MT005281.1	China	Homo sapiens	2015	D
MT005282.1	China	Homo sapiens	2015	D
MT005283.1	China	Homo sapiens	2015	D
MT413433.1	China	Tick	2017	D
LC536550.1	Japan	Haemaphysalis formosensis	2016	B
MT309102.1	China	Homo sapiens	2014	E
MT309103.1	China	Homo sapiens	2014	D
MT309104.1	China	Homo sapiens	2014	D
MT232961.1	China	Homo sapiens	2019	A
LC536551.1	Japan	Haemaphysalis formosensis	2016	B
OM452860.1	China	Homo sapiens	2020	F
KY965103.1	China	Homo sapiens	2016	B
OM452861.1	China	Homo sapiens	2020	F
MK524364.1	China	Homo sapiens	2017	D
OM452862.1	China	Homo sapiens	2020	F
OM452940.1	China	Homo sapiens	2020	F
KY965105.1	China	Homo sapiens	2016	B
OQ832098.1	China	Homo sapiens	2021	F
MK513911.1	China	Homo sapiens	2015	C
LC536552.1	Japan	Haemaphysalis formosensis	2016	B
OQ388963.1	China	Homo sapiens	2021	F
OQ388966.1	China	Homo sapiens	2021	F
MK513923.1	China	Homo sapiens	2015	A
MK513926.1	China	Homo sapiens	2015	A
OQ388974.1	China	Homo sapiens	2021	F
MK513932.1	China	Homo sapiens	2015	D
OQ388992.1	China	Homo sapiens	2021	F
OQ388965.1	China	Homo sapiens	2022	F
KY965092.1	China	Homo sapiens	2016	D
KY965093.1	China	Homo sapiens	2016	D
KY965094.1	China	Homo sapiens	2016	D
KY965095.1	China	Homo sapiens	2016	D
OQ388967.1	China	Homo sapiens	2022	F
OQ388969.1	China	Homo sapiens	2022	F
KY965098.1	China	Homo sapiens	2016	E
OQ388972.1	China	Homo sapiens	2022	F
KY965100.1	China	Homo sapiens	2016	A

OQ388973.1	China	Homo sapiens	2022	F
OQ388975.1	China	Homo sapiens	2022	F
LC536553.1	Japan	-	2016	B
OQ388976.1	China	Homo sapiens	2022	F
LC536554.1	Japan	-	2016	B
KY965106.1	China	Homo sapiens	2016	D
OQ388978.1	China	Homo sapiens	2022	F
OQ388979.1	China	Homo sapiens	2022	F
OQ388981.1	China	Homo sapiens	2022	F
OQ388982.1	China	Homo sapiens	2022	F
OQ388983.1	China	Homo sapiens	2022	F
LC536555.1	Japan	-	2016	B
KY273269.1	China	Homo sapiens	2013	A
MK524352.1	China	Homo sapiens	2017	B
KY273271.1	China	Homo sapiens	2014	A
KY933682.1	China	Homo sapiens	2014	D
OQ388984.1	China	Homo sapiens	2022	F
OQ388985.1	China	Homo sapiens	2022	F
KY933686.1	China	Homo sapiens	2014	A
MK524358.1	China	Homo sapiens	2017	B
MT683684.1	Korea	Homo sapiens	2017	B
MN450762.1	Korea	Haemaphysalis longicornis	2017	B
MH937373.1	Korea	Homo sapiens	2017	B
KY440770.1	China	Homo sapiens	2016	D
OQ388986.1	China	Homo sapiens	2022	F
KY440776.1	China	Homo sapiens	2015	D
OQ388987.1	China	Homo sapiens	2022	F
LC325236.1	Japan	Acinonyx jubatus (Cheetah)	2017	B
LC325237.1	Japan	Acinonyx jubatus (Cheetah)	2017	B
MZ501596.1	Korea	Dog	2018	B
LC462232.1	Japan	Felis catus	2018	B
KR698336.1	China	Homo sapiens	2013	A
LC462233.1	Japan	Homo sapiens	2018	B
LC462234.1	Japan	Homo sapiens	2018	B
KR698339.1	China	Homo sapiens	2013	A
KR698340.1	China	Homo sapiens	2013	A
KR698341.1	China	Homo sapiens	2013	A
LC590894.1	Japan	Homo sapiens	2018	B
LC570788.1	Japan	Dog	2018	B
OQ388956.1	China	Homo sapiens	2019	B
KT254590.1	China	Homo sapiens	2014	A
OQ388988.1	China	Homo sapiens	2022	F
OQ388989.1	China	Homo sapiens	2022	D

LC473515.1	Japan	Felis catus	2019	B
LC473516.1	Japan	Felis catus	2019	B
OQ388991.1	China	Homo sapiens	2022	F
ON402253.1	China	Homo sapiens	2022	F
JQ670930.1	China	Homo sapiens	2011	C
ON402254.1	China	Homo sapiens	2022	F
JQ733560.1	China	Homo sapiens	2011	D
ON402255.1	China	Homo sapiens	2022	F
HQ419230.1	China	-	2010	A
ON402256.1	China	Homo sapiens	2022	F
ON402257.1	China	Homo sapiens	2022	F
HQ419233.1	China	-	2010	A
HQ419234.1	China	-	2010	A
HQ419235.1	China	-	2010	D
HQ419236.1	China	-	2010	A
HQ419237.1	China	-	2010	A
HQ419238.1	China	-	2010	E
HM745931.1	China	Homo sapiens	2010	D
MN995253.1	Japan	Felis catus		B
MN995254.1	Japan	Felis catus		B
MN995255.1	Japan	Felis catus		B
MN995256.1	Japan	Felis catus		B
MN995257.1	Japan	Felis catus		B
MN995258.1	Japan	Felis catus		B
MN995259.1	Japan	Felis catus		B
MN995260.1	Japan	Felis catus		B
MN995261.1	Japan	Felis catus		B
MN995262.1	Japan	Felis catus		B
MN995263.1	Japan	Felis catus		B
MN995264.1	Japan	Felis catus		B
MN995265.1	Japan	Felis catus		B
MN995266.1	Japan	Felis catus		B
MN995267.1	Japan	Felis catus		B
MN995268.1	Japan	Felis catus		B
MN995269.1	Japan	Felis catus		B
MN995270.1	Japan	Felis catus		B
MN995271.1	Japan	Felis catus		B
MN995272.1	Japan	Felis catus		B
MN995273.1	Japan	Felis catus		B
MN995274.1	Japan	Felis catus		B
MN995275.1	Japan	Felis catus		B
MN995276.1	Japan	Felis catus		B
MN995277.1	Japan	Felis catus		B

MN995278.1	Japan	Felis catus		B
MN995279.1	Japan	Felis catus		B
MN995280.1	Japan	Felis catus		B
MN995281.1	Japan	Felis catus		B
LC570794.1	Japan	Dog	2019	B
LC570800.1	Japan	Dog	2019	B
LC570791.1	Japan	Dog	2019	B
LC549338.1	Japan	Homo sapiens	2019	B
LC473510.1	Japan	Felis catus	2019	B
LC473504.1	Japan	Felis catus	2019	B
LC473505.1	Japan	Felis catus	2019	B
LC473506.1	Japan	Felis catus	2019	B
LC473511.1	Japan	Felis catus	2019	B
LC473512.1	Japan	Felis catus	2019	B
LC473513.1	Japan	Felis catus	2019	B
LC473517.1	Japan	Felis catus	2019	B
LC473507.1	Japan	Felis catus	2019	B
LC473514.1	Japan	Felis catus	2019	B
LC473519.1	Japan	Felis catus	2019	B
LC473502.1	Japan	Felis catus	2019	A
LC473503.1	Japan	Felis catus	2019	A
LC473518.1	Japan	Felis catus	2019	B
LC579719.1	Japan	Procyon lotor	2013	C
LC473501.1	Japan	Felis catus	2019	B
OQ388957.1	China	Homo sapiens	2020	B
OQ388958.1	China	Homo sapiens	2020	B
OQ388996.1	China	Homo sapiens	2020	B
LC579716.1	Japan	Procyon lotor	2013	C
OQ388997.1	China	Homo sapiens	2020	B
OQ388998.1	China	Homo sapiens	2020	B
LC590891.1	Japan	Homo sapiens	2017	C
OM452787.1	China	Homo sapiens	2020	B
LC570785.1	Japan	Dog	2018	A
MZ773034.1	China	Homo sapiens	2020	B
MZ773035.1	China	Homo sapiens	2020	B
LC570797.1	Japan	Dog	2019	C
MZ773043.1	China	Homo sapiens	2020	B
MZ773044.1	China	Homo sapiens	2020	B
MZ501599.1	Korea	Dog	2020	B
LC473520.1	Japan	Felis catus	2019	C
MZ352108.1	Korea	Felis catus	2020	B
MN830174.1	China	Homo sapiens	2020	B
OQ388961.1	China	Homo sapiens	2021	B

OQ388964.1	China	Homo sapiens	2021	B
OQ388980.1	China	Homo sapiens	2021	B
OQ388993.1	China	Homo sapiens	2021	B
OQ388994.1	China	Homo sapiens	2021	B
LC473508.1	Japan	Felis catus	2019	A
OR797609.1	Korea	Dog	2021	B
LC663818.1	Japan	Homo sapiens	2021	B
LC663821.1	Japan	Felis catus	2021	B
LC730376.1	Japan	Homo sapiens	2021	B
OQ388971.1	China	Homo sapiens	2022	B
OQ388977.1	China	Homo sapiens	2022	B
OR797606.1	Korea	Dog	2022	B
LC715250.1	Japan	Felis catus	2022	B
LC705155.1	Japan	Felis catus	2022	B
ON402258.1	China	Homo sapiens	2022	F
LC705156.1	Japan	Felis catus	2022	B
AB985292.1	Japan	Homo sapiens	-	B
AB985293.1	Japan	Homo sapiens	-	B
AB985294.1	Japan	Homo sapiens	-	B
AB985295.1	Japan	Homo sapiens	-	B
AB985296.1	Japan	Homo sapiens	-	B
AB985297.1	Japan	Homo sapiens	-	B
AB985298.1	Japan	Homo sapiens	-	B
AB985299.1	Japan	Homo sapiens	-	B
AB985300.1	Japan	Homo sapiens	-	B
AB985301.1	Japan	Homo sapiens	-	B
AB985302.1	Japan	Homo sapiens	-	B
AB985303.1	Japan	Homo sapiens	-	B
AB985304.1	Japan	Homo sapiens	-	B
AB985305.1	Japan	Homo sapiens	-	B
AB985306.1	Japan	Homo sapiens	-	B
AB985307.1	Japan	Homo sapiens	-	B
AB985308.1	Japan	Homo sapiens	-	B
AB985309.1	Japan	Homo sapiens	-	B
AB985310.1	Japan	Homo sapiens	-	B
AB985311.1	Japan	Homo sapiens	-	B
AB985312.1	Japan	Homo sapiens	-	B
AB985313.1	Japan	Homo sapiens	-	B
AB985314.1	Japan	Homo sapiens	-	B
AB985315.1	Japan	Homo sapiens	-	B
AB985316.1	Japan	Homo sapiens	-	B
AB985317.1	Japan	Homo sapiens	-	B
AB985318.1	Japan	Homo sapiens	-	B

AB985319.1	Japan	Homo sapiens	-	B
AB985320.1	Japan	Homo sapiens	-	B
AB985321.1	Japan	Homo sapiens	-	B
AB985322.1	Japan	Homo sapiens	-	B
AB985323.1	Japan	Homo sapiens	-	B
AB985324.1	Japan	Homo sapiens	-	B
AB985325.1	Japan	Homo sapiens	-	B
AB985326.1	Japan	Homo sapiens	-	B
AB985635.1	Japan	Homo sapiens	-	D
AB985639.1	Japan	Homo sapiens	-	F
AB985647.1	Japan	Homo sapiens	-	B
AB985653.1	Japan	Homo sapiens	-	B
AB985654.1	Japan	Homo sapiens	-	B
AB817987.1	Japan	Homo sapiens	-	B
AB817988.1	Japan	Homo sapiens	-	B
AB817989.1	Japan	Homo sapiens	-	B
AB817990.1	Japan	Homo sapiens	-	B
AB817991.1	Japan	Homo sapiens	-	B
AB817992.1	Japan	Homo sapiens	-	B
AB817993.1	Japan	Homo sapiens	-	B
AB817994.1	Japan	Homo sapiens	-	B
AB821347.1	Japan	Homo sapiens	-	B
LC705157.1	Japan	Dog	-	B

Note: 1094 M fragments were classified, 985 human sequences, 96 animal sequences, 13 Blank Host.

Table: L Segment Lineage Division

number	country	host	collection year	lineage
HQ419227.1	China	-	2010	A
HQ171190.1	China	Homo sapiens	2010	A
MT005202.1	China	Homo sapiens	2011	A
OM452943.1	China	Homo sapiens	2012	A
OM452944.1	China	Homo sapiens	2012	A
OM452960.1	China	Homo sapiens	2012	A
MT005217.1	China	Homo sapiens	2012	A
OM452969.1	China	Homo sapiens	2013	A
OM452974.1	China	Homo sapiens	2013	A
OM452980.1	China	Homo sapiens	2013	A
OM452983.1	China	Homo sapiens	2013	A
OM452990.1	China	Homo sapiens	2013	A
OM452998.1	China	Homo sapiens	2013	A
OM452999.1	China	Homo sapiens	2013	A
OM453002.1	China	Homo sapiens	2013	A
OM453009.1	China	Homo sapiens	2013	A
OM453013.1	China	Homo sapiens	2013	A
OM453014.1	China	Homo sapiens	2013	A
OM453016.1	China	Homo sapiens	2013	A
OM453020.1	China	Homo sapiens	2013	A
OM453022.1	China	Homo sapiens	2013	A
OM453025.1	China	Homo sapiens	2013	A
OM453027.1	China	Homo sapiens	2013	A
OM453033.1	China	Homo sapiens	2013	A
OM453034.1	China	Homo sapiens	2013	A
OM453041.1	China	Homo sapiens	2013	A
OM453043.1	China	Homo sapiens	2013	A
OM453047.1	China	Homo sapiens	2013	A
OM453048.1	China	Homo sapiens	2013	A
OM453049.1	China	Homo sapiens	2013	A
OM453056.1	China	Homo sapiens	2013	A
OM453057.1	China	Homo sapiens	2013	A
OM453058.1	China	Homo sapiens	2013	A
OM453060.1	China	Homo sapiens	2013	A
MT005220.1	China	Homo sapiens	2013	A
MT005223.1	China	Homo sapiens	2013	A
KY273265.1	China	Homo sapiens	2013	A
KR698349.1	China	Homo sapiens	2013	A
KR698353.1	China	Homo sapiens	2013	A

KR698354.1	China	Homo sapiens	2013	A
LC579720.1	Japan	Procyon lotor	2013	A
NC_043450.1	China	Homo sapiens	2014	A
OM453062.1	China	Homo sapiens	2014	A
OM453065.1	China	Homo sapiens	2014	A
OM453071.1	China	Homo sapiens	2014	A
OM453074.1	China	Homo sapiens	2014	A
OM453075.1	China	Homo sapiens	2014	A
OM453080.1	China	Homo sapiens	2014	A
OM453083.1	China	Homo sapiens	2014	A
OM453085.1	China	Homo sapiens	2014	A
OM453089.1	China	Homo sapiens	2014	A
OM453092.1	China	Homo sapiens	2014	A
OM453093.1	China	Homo sapiens	2014	A
OM453095.1	China	Homo sapiens	2014	A
OM453097.1	China	Homo sapiens	2014	A
OM453101.1	China	Homo sapiens	2014	A
OM453105.1	China	Homo sapiens	2014	A
OM453109.1	China	Homo sapiens	2014	A
OM453112.1	China	Homo sapiens	2014	A
OM453114.1	China	Homo sapiens	2014	A
OM453115.1	China	Homo sapiens	2014	A
OM453119.1	China	Homo sapiens	2014	A
OM453120.1	China	Homo sapiens	2014	A
OM453121.1	China	Homo sapiens	2014	A
OM453129.1	China	Homo sapiens	2014	A
OM453133.1	China	Homo sapiens	2014	A
OM453142.1	China	Homo sapiens	2014	A
OM453151.1	China	Homo sapiens	2014	A
KY273267.1	China	Homo sapiens	2014	A
KT254588.1	China	Homo sapiens	2014	A
OM453162.1	China	Homo sapiens	2015	A
OM453167.1	China	Homo sapiens	2015	A
OM453170.1	China	Homo sapiens	2015	A
OM453171.1	China	Homo sapiens	2015	A
OM453176.1	China	Homo sapiens	2015	A
OM453180.1	China	Homo sapiens	2015	A
OM453184.1	China	Homo sapiens	2015	A
OM453186.1	China	Homo sapiens	2015	A
OM453198.1	China	Homo sapiens	2015	A
OM453204.1	China	Homo sapiens	2015	A
OM453209.1	China	Homo sapiens	2015	A
OM453215.1	China	Homo sapiens	2015	A

OM453217.1	China	Homo sapiens	2015	A
OM453229.1	China	Homo sapiens	2015	A
MK513912.1	China	Homo sapiens	2015	A
MK513924.1	China	Homo sapiens	2015	A
MK513927.1	China	Homo sapiens	2015	A
OM453231.1	China	Homo sapiens	2016	A
OM453236.1	China	Homo sapiens	2016	A
OM453238.1	China	Homo sapiens	2016	A
OM453239.1	China	Homo sapiens	2016	A
OM453243.1	China	Homo sapiens	2016	A
OM453245.1	China	Homo sapiens	2016	A
OM453247.1	China	Homo sapiens	2016	A
OM453252.1	China	Homo sapiens	2016	A
OM453254.1	China	Homo sapiens	2016	A
OM453255.1	China	Homo sapiens	2016	A
OM453257.1	China	Homo sapiens	2016	A
OM453258.1	China	Homo sapiens	2016	A
OM453259.1	China	Homo sapiens	2016	A
OM453260.1	China	Homo sapiens	2016	A
OM453262.1	China	Homo sapiens	2016	A
OM453265.1	China	Homo sapiens	2016	A
OM453270.1	China	Homo sapiens	2016	A
KY965118.1	China	Homo sapiens	2016	A
OM453272.1	China	Homo sapiens	2017	A
OM453276.1	China	Homo sapiens	2017	A
OM453279.1	China	Homo sapiens	2017	A
OM453298.1	China	Homo sapiens	2017	A
OM453333.1	China	Homo sapiens	2017	A
OM453346.1	China	Homo sapiens	2017	A
OM453354.1	China	Homo sapiens	2017	A
OM453371.1	China	Homo sapiens	2017	A
OM453372.1	China	Homo sapiens	2017	A
OM453398.1	China	Homo sapiens	2017	A
OM453401.1	China	Homo sapiens	2017	A
OM453418.1	China	Homo sapiens	2017	A
MT320796.1	China	Homo sapiens	2017	A
MT320799.1	China	Homo sapiens	2017	A
MH049431.1	China	Haemaphysalis longicornis	2017	A
LC590890.1	Japan	Homo sapiens	2017	A
OM453283.1	China	Homo sapiens	2018	A
OM453286.1	China	Homo sapiens	2018	A
OM453290.1	China	Homo sapiens	2018	A
OM453295.1	China	Homo sapiens	2018	A
OM453308.1	China	Homo sapiens	2018	A

OM453311.1	China	Homo sapiens	2018	A
OM453312.1	China	Homo sapiens	2018	A
OM453335.1	China	Homo sapiens	2018	A
OM453340.1	China	Homo sapiens	2018	A
OM453347.1	China	Homo sapiens	2018	A
OM453360.1	China	Homo sapiens	2018	A
OM453362.1	China	Homo sapiens	2018	A
OM453366.1	China	Homo sapiens	2018	A
OM453369.1	China	Homo sapiens	2018	A
OM453399.1	China	Homo sapiens	2018	A
OM453400.1	China	Homo sapiens	2018	A
OM453402.1	China	Homo sapiens	2018	A
OM453411.1	China	Homo sapiens	2018	A
OM453419.1	China	Homo sapiens	2018	A
LC570786.1	Japan	Dog	2018	A
OM453432.1	China	Homo sapiens	2019	A
OM453436.1	China	Homo sapiens	2019	A
OM453437.1	China	Homo sapiens	2019	A
OM453441.1	China	Homo sapiens	2019	A
OM453544.1	China	Homo sapiens	2019	A
OM453550.1	China	Homo sapiens	2019	A
OM453553.1	China	Homo sapiens	2019	A
OM453557.1	China	Homo sapiens	2019	A
OM453568.1	China	Homo sapiens	2019	A
OM453570.1	China	Homo sapiens	2019	A
OM453571.1	China	Homo sapiens	2019	A
OM453595.1	China	Homo sapiens	2019	A
OM453607.1	China	Homo sapiens	2019	A
OM453610.1	China	Homo sapiens	2019	A
MT232962.1	China	Homo sapiens	2019	A
OQ388746.1	China	Homo sapiens	2020	A
OM453447.1	China	Homo sapiens	2020	A
OM453448.1	China	Homo sapiens	2020	A
OM453449.1	China	Homo sapiens	2020	A
OM453458.1	China	Homo sapiens	2020	A
OM453462.1	China	Homo sapiens	2020	A
OM453468.1	China	Homo sapiens	2020	A
OM453470.1	China	Homo sapiens	2020	A
OM453471.1	China	Homo sapiens	2020	A
OM453475.1	China	Homo sapiens	2020	A
OM453477.1	China	Homo sapiens	2020	A
OM453480.1	China	Homo sapiens	2020	A
OM453481.1	China	Homo sapiens	2020	A
OM453491.1	China	Homo sapiens	2020	A

OM453494.1	China	Homo sapiens	2020	A
OM453496.1	China	Homo sapiens	2020	A
OM453501.1	China	Homo sapiens	2020	A
OM453503.1	China	Homo sapiens	2020	A
OM453507.1	China	Homo sapiens	2020	A
OM453508.1	China	Homo sapiens	2020	A
OM453513.1	China	Homo sapiens	2020	A
OM453536.1	China	Homo sapiens	2020	A
OM453542.1	China	Homo sapiens	2020	A
MZ773015.1	China	Homo sapiens	2020	A
MZ773016.1	China	Homo sapiens	2020	A
MZ773017.1	China	Homo sapiens	2020	A
MZ773018.1	China	Homo sapiens	2020	A
MZ773021.1	China	Homo sapiens	2020	A
MZ773022.1	China	Homo sapiens	2020	A
MZ773023.1	China	Homo sapiens	2020	A
MZ773024.1	China	Homo sapiens	2020	A
MZ773025.1	China	Homo sapiens	2020	A
MZ773026.1	China	Homo sapiens	2020	A
MZ773027.1	China	Homo sapiens	2020	A
OQ388754.1	China	Homo sapiens	2022	A
OM452966.1	China	Homo sapiens	2012	B
KY273264.1	China	Homo sapiens	2012	B
KY273136.1	Korea	Ticks	2013	B
KU507543.1	Korea	Homo sapiens	2013	B
KU507544.1	Korea	Homo sapiens	2013	B
KU507545.1	Korea	Homo sapiens	2013	B
KU507546.1	Korea	Homo sapiens	2013	B
KU507547.1	Korea	Homo sapiens	2013	B
KR698345.1	China	Homo sapiens	2013	B
KR698346.1	China	Homo sapiens	2013	B
KR698347.1	China	Homo sapiens	2013	B
KR698348.1	China	Homo sapiens	2013	B
KR698350.1	China	Homo sapiens	2013	B
KR698351.1	China	Homo sapiens	2013	B
KR698352.1	China	Homo sapiens	2013	B
KP280206.1	China	Homo sapiens	2013	B
KF374682.1	China	Homo sapiens	2013	B
MZ501601.1	Korea	Goat	2014	B
MT236315.1	China	Ticks	2014	B
KY273266.1	China	Homo sapiens	2014	B
KY933704.1	China	Homo sapiens	2014	B
KY933705.1	China	Homo sapiens	2014	B
KY933706.1	China	Homo sapiens	2014	B

KY933707.1	China	Homo sapiens	2014	B
KR698355.1	China	Homo sapiens	2014	B
KR698356.1	China	Apodemus agrarius ningpoensis	2014	B
KR698357.1	China	Apodemus agrarius ningpoensis	2014	B
LC579711.1	Japan	Procyon lotor	2014	B
LC579714.1	Japan	Procyon lotor	2014	B
MH491549.1	Korea	Homo sapiens	2015	B
MN329150.1	Korea	Homo sapiens	2015	B
MG920819.1	Korea	Homo sapiens	2015	B
MG920822.1	Korea	Homo sapiens	2015	B
MG920825.1	Korea	Homo sapiens	2015	B
MG920828.1	Korea	Homo sapiens	2015	B
MG921166.1	Korea	Homo sapiens	2015	B
MG921169.1	Korea	Homo sapiens	2015	B
MK513906.1	China	Homo sapiens	2015	B
MK513915.1	China	Homo sapiens	2015	B
MH464252.1	Korea	Dog	2016	B
KY965121.1	China	Homo sapiens	2016	B
KY965123.1	China	Homo sapiens	2016	B
LC536556.1	Japan	Haemaphysalis formosensis	2016	B
LC536557.1	Japan	Amblyomma testudinarium	2016	B
LC536558.1	Japan	Amblyomma testudinarium	2016	B
LC536559.1	Japan	Haemaphysalis flava	2016	B
LC536560.1	Japan	Haemaphysalis formosensis	2016	B
LC536561.1	Japan	Haemaphysalis formosensis	2016	B
LC536562.1	Japan	Haemaphysalis formosensis	2016	B
LC536563.1	Japan	-	2016	B
LC536564.1	Japan	-	2016	B
LC536565.1	Japan	-	2016	B
MT683683.1	Korea	Homo sapiens	2017	B
MN450761.1	Korea	Haemaphysalis longicornis	2017	B
MH937374.1	Korea	Homo sapiens	2017	B
MK524359.1	China	Homo sapiens	2017	B
LC325234.1	Japan	Acinonyx jubatus (Cheetah)	2017	B
LC325235.1	Japan	Acinonyx jubatus (Cheetah)	2017	B
MZ501595.1	Korea	Dog	2018	B
LC462235.1	Japan	Felis catus	2018	B
LC462236.1	Japan	Homo sapiens	2018	B
LC462237.1	Japan	Homo sapiens	2018	B
LC570789.1	Japan	Dog	2018	B
LC590893.1	Japan	Homo sapiens	2018	B
OQ388742.1	China	Homo sapiens	2019	B

ON812166.1	China	Haemaphysalis hystricis	2019	B
LC549339.1	Japan	Homo sapiens	2019	B
LC570792.1	Japan	Dog	2019	B
LC570795.1	Japan	Dog	2019	B
LC570801.1	Japan	Dog	2019	B
MZ501598.1	Korea	Dog	2020	B
OQ388743.1	China	Homo sapiens	2020	B
OQ388744.1	China	Homo sapiens	2020	B
OQ388782.1	China	Homo sapiens	2020	B
OQ388783.1	China	Homo sapiens	2020	B
OQ388785.1	China	Homo sapiens	2020	B
OM453466.1	China	Homo sapiens	2020	B
MZ773019.1	China	Homo sapiens	2020	B
MZ773020.1	China	Homo sapiens	2020	B
MZ773028.1	China	Homo sapiens	2020	B
MZ773029.1	China	Homo sapiens	2020	B
MN910270.1	China	Homo sapiens	2020	B
OR797610.1	Korea	Dog	2021	B
OQ388747.1	China	Homo sapiens	2021	B
OQ388750.1	China	Homo sapiens	2021	B
OQ388760.1	China	Homo sapiens	2021	B
OQ388778.1	China	Homo sapiens	2021	B
OQ388780.1	China	Homo sapiens	2021	B
LC663819.1	Japan	Homo sapiens	2021	B
LC663822.1	Japan	Felis catus	2021	B
LC732568.1	Japan	Homo sapiens	2021	B
OR797605.1	Korea	Dog	2022	B
OQ388757.1	China	Homo sapiens	2022	B
OQ388758.1	China	Homo sapiens	2022	B
OQ388764.1	China	Homo sapiens	2022	B
OR574965.1	China	Homo sapiens	2023	B
MK513909.1	China	Homo sapiens	2015	B
JQ670934.1	China	Homo sapiens	2011	C
LC579717.1	Japan	Procyon lotor	2013	C
LC570798.1	Japan	Dog	2019	C
NC_018136.1	China	Homo sapiens	2010	D
HQ419226.1	China	-	2010	D
HQ171188.1	China	Homo sapiens	2010	D
HM745930.1	China	Homo sapiens	2010	D
MT005201.1	China	Homo sapiens	2011	D
MT005203.1	China	Homo sapiens	2011	D
MT005204.1	China	Homo sapiens	2011	D
MT005205.1	China	Homo sapiens	2011	D

MT005206.1	China	Homo sapiens	2011	D
MT005207.1	China	Homo sapiens	2011	D
MT005208.1	China	Homo sapiens	2011	D
MT005209.1	China	Homo sapiens	2011	D
MT005210.1	China	Homo sapiens	2011	D
MT005211.1	China	Homo sapiens	2011	D
MT005212.1	China	Homo sapiens	2011	D
OM452957.1	China	Homo sapiens	2012	D
OM452958.1	China	Homo sapiens	2012	D
MT005214.1	China	Homo sapiens	2012	D
MT005215.1	China	Homo sapiens	2012	D
MT005216.1	China	Homo sapiens	2012	D
OM452975.1	China	Homo sapiens	2013	D
OM452991.1	China	Homo sapiens	2013	D
OM452994.1	China	Homo sapiens	2013	D
OM453005.1	China	Homo sapiens	2013	D
OM453007.1	China	Homo sapiens	2013	D
OM453010.1	China	Homo sapiens	2013	D
OM453017.1	China	Homo sapiens	2013	D
OM453018.1	China	Homo sapiens	2013	D
OM453029.1	China	Homo sapiens	2013	D
OM453032.1	China	Homo sapiens	2013	D
OM453037.1	China	Homo sapiens	2013	D
MT005219.1	China	Homo sapiens	2013	D
MT005221.1	China	Homo sapiens	2013	D
MT005222.1	China	Homo sapiens	2013	D
MT005224.1	China	Homo sapiens	2013	D
MT005225.1	China	Homo sapiens	2013	D
MT005226.1	China	Homo sapiens	2013	D
MT005227.1	China	Homo sapiens	2013	D
MT005228.1	China	Homo sapiens	2013	D
OM453067.1	China	Homo sapiens	2014	D
OM453088.1	China	Homo sapiens	2014	D
OM453090.1	China	Homo sapiens	2014	D
OM453116.1	China	Homo sapiens	2014	D
OM453117.1	China	Homo sapiens	2014	D
OM453122.1	China	Homo sapiens	2014	D
OM453127.1	China	Homo sapiens	2014	D
MT005229.1	China	Homo sapiens	2014	D
MT005230.1	China	Homo sapiens	2014	D
MT005231.1	China	Homo sapiens	2014	D
MT005232.1	China	Homo sapiens	2014	D
MT005234.1	China	Homo sapiens	2014	D
MT005235.1	China	Homo sapiens	2014	D

MT005236.1	China	Homo sapiens	2014	D
MT309099.1	China	Homo sapiens	2014	D
MT309100.1	China	Homo sapiens	2014	D
MT309101.1	China	Homo sapiens	2014	D
OM453163.1	China	Homo sapiens	2015	D
OM453179.1	China	Homo sapiens	2015	D
OM453181.1	China	Homo sapiens	2015	D
OM453182.1	China	Homo sapiens	2015	D
OM453188.1	China	Homo sapiens	2015	D
OM453196.1	China	Homo sapiens	2015	D
OM453205.1	China	Homo sapiens	2015	D
OM453206.1	China	Homo sapiens	2015	D
OM453210.1	China	Homo sapiens	2015	D
OM453212.1	China	Homo sapiens	2015	D
OM453224.1	China	Homo sapiens	2015	D
MT005237.1	China	Homo sapiens	2015	D
MT005238.1	China	Homo sapiens	2015	D
MT005240.1	China	Homo sapiens	2015	D
MT005241.1	China	Homo sapiens	2015	D
MK513921.1	China	Homo sapiens	2015	D
MK513933.1	China	Homo sapiens	2015	D
KY440777.1	China	Homo sapiens	2015	D
OM453154.1	China	Homo sapiens	2016	D
OM453161.1	China	Homo sapiens	2016	D
OM453207.1	China	Homo sapiens	2016	D
OM453246.1	China	Homo sapiens	2016	D
OM453256.1	China	Homo sapiens	2016	D
OM453266.1	China	Homo sapiens	2016	D
OM453271.1	China	Homo sapiens	2016	D
KY965109.1	China	Homo sapiens	2016	D
KY965111.1	China	Homo sapiens	2016	D
KY965112.1	China	Homo sapiens	2016	D
KY965113.1	China	Homo sapiens	2016	D
KY965120.1	China	Homo sapiens	2016	D
KY965124.1	China	Homo sapiens	2016	D
KY440771.1	China	Homo sapiens	2016	D
OM453273.1	China	Homo sapiens	2017	D
OM453274.1	China	Homo sapiens	2017	D
OM453275.1	China	Homo sapiens	2017	D
OM453277.1	China	Homo sapiens	2017	D
OM453278.1	China	Homo sapiens	2017	D
OM453318.1	China	Homo sapiens	2017	D
OM453330.1	China	Homo sapiens	2017	D
OM453367.1	China	Homo sapiens	2017	D

OM453375.1	China	Homo sapiens	2017	D
MT320787.1	China	Homo sapiens	2017	D
MT320790.1	China	Homo sapiens	2017	D
MT320793.1	China	Homo sapiens	2017	D
MT320814.1	China	Homo sapiens	2017	D
MT320817.1	China	Homo sapiens	2017	D
MT413432.1	China	Ticks	2017	D
MK524353.1	China	Homo sapiens	2017	D
MK524365.1	China	Homo sapiens	2017	D
OM453289.1	China	Homo sapiens	2018	D
OM453293.1	China	Homo sapiens	2018	D
OM453309.1	China	Homo sapiens	2018	D
OM453317.1	China	Homo sapiens	2018	D
OM453322.1	China	Homo sapiens	2018	D
OM453327.1	China	Homo sapiens	2018	D
OM453334.1	China	Homo sapiens	2018	D
OM453341.1	China	Homo sapiens	2018	D
OM453355.1	China	Homo sapiens	2018	D
OM453358.1	China	Homo sapiens	2018	D
OM453368.1	China	Homo sapiens	2018	D
OM453390.1	China	Homo sapiens	2018	D
OM453406.1	China	Homo sapiens	2018	D
OM453417.1	China	Homo sapiens	2018	D
OM453425.1	China	Homo sapiens	2019	D
OM453428.1	China	Homo sapiens	2019	D
OM453431.1	China	Homo sapiens	2019	D
OM453434.1	China	Homo sapiens	2019	D
OM453543.1	China	Homo sapiens	2019	D
OM453552.1	China	Homo sapiens	2019	D
OM453592.1	China	Homo sapiens	2019	D
OM453597.1	China	Homo sapiens	2019	D
OM453598.1	China	Homo sapiens	2019	D
OM453613.1	China	Homo sapiens	2019	D
OM453614.1	China	Homo sapiens	2019	D
OM453617.1	China	Homo sapiens	2019	D
MW721873.1	China	Haemaphysalis longicornis	2019	D
MW721874.1	China	Haemaphysalis longicornis	2019	D
MZ965009.1	China	Haemaphysalis longicornis	2019	D
MZ965010.1	China	Haemaphysalis longicornis	2019	D
OQ388745.1	China	Homo sapiens	2020	D
OQ388781.1	China	Homo sapiens	2020	D
OM453451.1	China	Homo sapiens	2020	D
OM453467.1	China	Homo sapiens	2020	D
OM453472.1	China	Homo sapiens	2020	D

OM453498.1	China	Homo sapiens	2020	D
OM453514.1	China	Homo sapiens	2020	D
OM453515.1	China	Homo sapiens	2020	D
OM453516.1	China	Homo sapiens	2020	D
OM453529.1	China	Homo sapiens	2020	D
OM453533.1	China	Homo sapiens	2020	D
OM453619.1	China	Homo sapiens	2020	D
OM453620.1	China	Homo sapiens	2020	D
OM453621.1	China	Homo sapiens	2020	D
OQ832099.1	China	Homo sapiens	2021	D
OQ388749.1	China	Homo sapiens	2021	D
OQ388751.1	China	Homo sapiens	2022	D
OQ388755.1	China	Homo sapiens	2022	D
OQ388756.1	China	Homo sapiens	2022	D
OQ388759.1	China	Homo sapiens	2022	D
OQ388765.1	China	Homo sapiens	2022	D
OQ388766.1	China	Homo sapiens	2022	D
OQ388767.1	China	Homo sapiens	2022	D
OQ388769.1	China	Homo sapiens	2022	D
OQ388771.1	China	Homo sapiens	2022	D
OQ388774.1	China	Homo sapiens	2022	D
OQ388775.1	China	Homo sapiens	2022	D
OQ388776.1	China	Homo sapiens	2022	D
MT005239.1	China	Homo sapiens	2015	D
MT005200.1	China	Homo sapiens	2011	E
MT005213.1	China	Homo sapiens	2012	E
MT005218.1	China	Homo sapiens	2013	E
MT005233.1	China	Homo sapiens	2014	E
MK513930.1	China	Homo sapiens	2015	E
KY965117.1	China	Homo sapiens	2016	E
KY965122.1	China	Homo sapiens	2016	E
MK524371.1	China	Homo sapiens	2017	E
HQ171186.1	China	Homo sapiens	2009	F
JQ684871.1	China	Haemaphysalis longicornis	2010	F
HQ419228.1	China	-	2010	F
HQ171187.1	China	Homo sapiens	2010	F
HQ171189.1	China	Homo sapiens	2010	F
OR260285.1	China	Homo sapiens	2011	F
JQ670929.1	China	Homo sapiens	2011	F
KF358691.1	Korea	Homo sapiens	2012	F
OM452945.1	China	Homo sapiens	2012	F
OM452946.1	China	Homo sapiens	2012	F
OM452947.1	China	Homo sapiens	2012	F
OM452948.1	China	Homo sapiens	2012	F

OM452949.1	China	Homo sapiens	2012	F
OM452950.1	China	Homo sapiens	2012	F
OM452951.1	China	Homo sapiens	2012	F
OM452952.1	China	Homo sapiens	2012	F
OM452953.1	China	Homo sapiens	2012	F
OM452954.1	China	Homo sapiens	2012	F
OM452955.1	China	Homo sapiens	2012	F
OM452956.1	China	Homo sapiens	2012	F
OM452959.1	China	Homo sapiens	2012	F
OM452961.1	China	Homo sapiens	2012	F
OM452962.1	China	Homo sapiens	2012	F
OM452963.1	China	Homo sapiens	2012	F
OM452964.1	China	Homo sapiens	2012	F
OM452965.1	China	Homo sapiens	2012	F
KC473540.1	China	Haemaphysalis longicornis	2012	F
OM452967.1	China	Homo sapiens	2013	F
OM452968.1	China	Homo sapiens	2013	F
OM452970.1	China	Homo sapiens	2013	F
OM452971.1	China	Homo sapiens	2013	F
OM452972.1	China	Homo sapiens	2013	F
OM452973.1	China	Homo sapiens	2013	F
OM452976.1	China	Homo sapiens	2013	F
OM452977.1	China	Homo sapiens	2013	F
OM452978.1	China	Homo sapiens	2013	F
OM452979.1	China	Homo sapiens	2013	F
OM452981.1	China	Homo sapiens	2013	F
OM452982.1	China	Homo sapiens	2013	F
OM452984.1	China	Homo sapiens	2013	F
OM452985.1	China	Homo sapiens	2013	F
OM452986.1	China	Homo sapiens	2013	F
OM452987.1	China	Homo sapiens	2013	F
OM452988.1	China	Homo sapiens	2013	F
OM452989.1	China	Homo sapiens	2013	F
OM452992.1	China	Homo sapiens	2013	F
OM452993.1	China	Homo sapiens	2013	F
OM452995.1	China	Homo sapiens	2013	F
OM452996.1	China	Homo sapiens	2013	F
OM452997.1	China	Homo sapiens	2013	F
OM453000.1	China	Homo sapiens	2013	F
OM453001.1	China	Homo sapiens	2013	F
OM453003.1	China	Homo sapiens	2013	F
OM453004.1	China	Homo sapiens	2013	F
OM453006.1	China	Homo sapiens	2013	F
OM453008.1	China	Homo sapiens	2013	F

OM453011.1	China	Homo sapiens	2013	F
OM453012.1	China	Homo sapiens	2013	F
OM453015.1	China	Homo sapiens	2013	F
OM453019.1	China	Homo sapiens	2013	F
OM453021.1	China	Homo sapiens	2013	F
OM453023.1	China	Homo sapiens	2013	F
OM453024.1	China	Homo sapiens	2013	F
OM453026.1	China	Homo sapiens	2013	F
OM453028.1	China	Homo sapiens	2013	F
OM453030.1	China	Homo sapiens	2013	F
OM453031.1	China	Homo sapiens	2013	F
OM453035.1	China	Homo sapiens	2013	F
OM453036.1	China	Homo sapiens	2013	F
OM453038.1	China	Homo sapiens	2013	F
OM453039.1	China	Homo sapiens	2013	F
OM453040.1	China	Homo sapiens	2013	F
OM453042.1	China	Homo sapiens	2013	F
OM453044.1	China	Homo sapiens	2013	F
OM453045.1	China	Homo sapiens	2013	F
OM453046.1	China	Homo sapiens	2013	F
OM453050.1	China	Homo sapiens	2013	F
OM453051.1	China	Homo sapiens	2013	F
OM453052.1	China	Homo sapiens	2013	F
OM453053.1	China	Homo sapiens	2013	F
OM453054.1	China	Homo sapiens	2013	F
OM453055.1	China	Homo sapiens	2013	F
OM453059.1	China	Homo sapiens	2013	F
KU361343.1	China	Homo sapiens	2013	F
OM453061.1	China	Homo sapiens	2014	F
OM453063.1	China	Homo sapiens	2014	F
OM453064.1	China	Homo sapiens	2014	F
OM453066.1	China	Homo sapiens	2014	F
OM453068.1	China	Homo sapiens	2014	F
OM453069.1	China	Homo sapiens	2014	F
OM453070.1	China	Homo sapiens	2014	F
OM453072.1	China	Homo sapiens	2014	F
OM453073.1	China	Homo sapiens	2014	F
OM453076.1	China	Homo sapiens	2014	F
OM453077.1	China	Homo sapiens	2014	F
OM453078.1	China	Homo sapiens	2014	F
OM453079.1	China	Homo sapiens	2014	F
OM453081.1	China	Homo sapiens	2014	F
OM453082.1	China	Homo sapiens	2014	F
OM453084.1	China	Homo sapiens	2014	F

OM453086.1	China	Homo sapiens	2014	F
OM453087.1	China	Homo sapiens	2014	F
OM453091.1	China	Homo sapiens	2014	F
OM453094.1	China	Homo sapiens	2014	F
OM453096.1	China	Homo sapiens	2014	F
OM453098.1	China	Homo sapiens	2014	F
OM453099.1	China	Homo sapiens	2014	F
OM453100.1	China	Homo sapiens	2014	F
OM453102.1	China	Homo sapiens	2014	F
OM453103.1	China	Homo sapiens	2014	F
OM453104.1	China	Homo sapiens	2014	F
OM453106.1	China	Homo sapiens	2014	F
OM453107.1	China	Homo sapiens	2014	F
OM453108.1	China	Homo sapiens	2014	F
OM453110.1	China	Homo sapiens	2014	F
OM453111.1	China	Homo sapiens	2014	F
OM453113.1	China	Homo sapiens	2014	F
OM453118.1	China	Homo sapiens	2014	F
OM453123.1	China	Homo sapiens	2014	F
OM453124.1	China	Homo sapiens	2014	F
OM453125.1	China	Homo sapiens	2014	F
OM453126.1	China	Homo sapiens	2014	F
OM453128.1	China	Homo sapiens	2014	F
OM453130.1	China	Homo sapiens	2014	F
OM453131.1	China	Homo sapiens	2014	F
OM453132.1	China	Homo sapiens	2014	F
OM453134.1	China	Homo sapiens	2014	F
OM453135.1	China	Homo sapiens	2014	F
OM453136.1	China	Homo sapiens	2014	F
OM453137.1	China	Homo sapiens	2014	F
OM453138.1	China	Homo sapiens	2014	F
OM453139.1	China	Homo sapiens	2014	F
OM453140.1	China	Homo sapiens	2014	F
OM453141.1	China	Homo sapiens	2014	F
OM453143.1	China	Homo sapiens	2014	F
OM453144.1	China	Homo sapiens	2014	F
OM453145.1	China	Homo sapiens	2014	F
OM453146.1	China	Homo sapiens	2014	F
OM453147.1	China	Homo sapiens	2014	F
OM453148.1	China	Homo sapiens	2014	F
OM453149.1	China	Homo sapiens	2014	F
OM453150.1	China	Homo sapiens	2014	F
OM453152.1	China	Homo sapiens	2014	F
OM453153.1	China	Homo sapiens	2014	F

KY933696.1	China	Homo sapiens	2014	F
KY933697.1	China	Homo sapiens	2014	F
KY933698.1	China	Homo sapiens	2014	F
KY933700.1	China	Homo sapiens	2014	F
KY933701.1	China	Homo sapiens	2014	F
KY933702.1	China	Homo sapiens	2014	F
KY933703.1	China	Homo sapiens	2014	F
KR706567.1	China	Homo sapiens	2014	F
OM453155.1	China	Homo sapiens	2015	F
OM453156.1	China	Homo sapiens	2015	F
OM453157.1	China	Homo sapiens	2015	F
OM453158.1	China	Homo sapiens	2015	F
OM453159.1	China	Homo sapiens	2015	F
OM453160.1	China	Homo sapiens	2015	F
OM453164.1	China	Homo sapiens	2015	F
OM453165.1	China	Homo sapiens	2015	F
OM453166.1	China	Homo sapiens	2015	F
OM453168.1	China	Homo sapiens	2015	F
OM453169.1	China	Homo sapiens	2015	F
OM453172.1	China	Homo sapiens	2015	F
OM453173.1	China	Homo sapiens	2015	F
OM453174.1	China	Homo sapiens	2015	F
OM453175.1	China	Homo sapiens	2015	F
OM453177.1	China	Homo sapiens	2015	F
OM453178.1	China	Homo sapiens	2015	F
OM453183.1	China	Homo sapiens	2015	F
OM453185.1	China	Homo sapiens	2015	F
OM453187.1	China	Homo sapiens	2015	F
OM453189.1	China	Homo sapiens	2015	F
OM453190.1	China	Homo sapiens	2015	F
OM453191.1	China	Homo sapiens	2015	F
OM453192.1	China	Homo sapiens	2015	F
OM453193.1	China	Homo sapiens	2015	F
OM453194.1	China	Homo sapiens	2015	F
OM453195.1	China	Homo sapiens	2015	F
OM453197.1	China	Homo sapiens	2015	F
OM453199.1	China	Homo sapiens	2015	F
OM453200.1	China	Homo sapiens	2015	F
OM453201.1	China	Homo sapiens	2015	F
OM453202.1	China	Homo sapiens	2015	F
OM453203.1	China	Homo sapiens	2015	F
OM453208.1	China	Homo sapiens	2015	F
OM453211.1	China	Homo sapiens	2015	F
OM453213.1	China	Homo sapiens	2015	F

OM453214.1	China	Homo sapiens	2015	F
OM453216.1	China	Homo sapiens	2015	F
OM453218.1	China	Homo sapiens	2015	F
OM453219.1	China	Homo sapiens	2015	F
OM453220.1	China	Homo sapiens	2015	F
OM453221.1	China	Homo sapiens	2015	F
OM453222.1	China	Homo sapiens	2015	F
OM453223.1	China	Homo sapiens	2015	F
OM453225.1	China	Homo sapiens	2015	F
OM453226.1	China	Homo sapiens	2015	F
OM453227.1	China	Homo sapiens	2015	F
OM453228.1	China	Homo sapiens	2015	F
OM453230.1	China	Homo sapiens	2015	F
MK513918.1	China	Homo sapiens	2015	F
MF140447.1	China	Rat	2015	F
KY933695.1	China	Homo sapiens	2015	F
OM453232.1	China	Homo sapiens	2016	F
OM453233.1	China	Homo sapiens	2016	F
OM453234.1	China	Homo sapiens	2016	F
OM453235.1	China	Homo sapiens	2016	F
OM453237.1	China	Homo sapiens	2016	F
OM453240.1	China	Homo sapiens	2016	F
OM453241.1	China	Homo sapiens	2016	F
OM453242.1	China	Homo sapiens	2016	F
OM453244.1	China	Homo sapiens	2016	F
OM453248.1	China	Homo sapiens	2016	F
OM453249.1	China	Homo sapiens	2016	F
OM453250.1	China	Homo sapiens	2016	F
OM453251.1	China	Homo sapiens	2016	F
OM453253.1	China	Homo sapiens	2016	F
OM453261.1	China	Homo sapiens	2016	F
OM453263.1	China	Homo sapiens	2016	F
OM453264.1	China	Homo sapiens	2016	F
OM453267.1	China	Homo sapiens	2016	F
OM453268.1	China	Homo sapiens	2016	F
OM453269.1	China	Homo sapiens	2016	F
KY773990.1	China	Haemaphysalis longicornis	2016	F
KY965110.1	China	Homo sapiens	2016	F
KY965114.1	China	Homo sapiens	2016	F
KY965115.1	China	Homo sapiens	2016	F
KY965116.1	China	Homo sapiens	2016	F
KY965119.1	China	Homo sapiens	2016	F
KY965125.1	China	Homo sapiens	2016	F
KY965126.1	China	Homo sapiens	2016	F

KY440774.1	China	Homo sapiens	2016	F
OM453280.1	China	Homo sapiens	2017	F
OM453281.1	China	Homo sapiens	2017	F
OM453284.1	China	Homo sapiens	2017	F
OM453287.1	China	Homo sapiens	2017	F
OM453288.1	China	Homo sapiens	2017	F
OM453292.1	China	Homo sapiens	2017	F
OM453294.1	China	Homo sapiens	2017	F
OM453296.1	China	Homo sapiens	2017	F
OM453299.1	China	Homo sapiens	2017	F
OM453303.1	China	Homo sapiens	2017	F
OM453304.1	China	Homo sapiens	2017	F
OM453305.1	China	Homo sapiens	2017	F
OM453316.1	China	Homo sapiens	2017	F
OM453323.1	China	Homo sapiens	2017	F
OM453349.1	China	Homo sapiens	2017	F
OM453352.1	China	Homo sapiens	2017	F
OM453356.1	China	Homo sapiens	2017	F
OM453363.1	China	Homo sapiens	2017	F
OM453364.1	China	Homo sapiens	2017	F
OM453365.1	China	Homo sapiens	2017	F
OM453370.1	China	Homo sapiens	2017	F
OM453376.1	China	Homo sapiens	2017	F
OM453377.1	China	Homo sapiens	2017	F
OM453379.1	China	Homo sapiens	2017	F
OM453383.1	China	Homo sapiens	2017	F
OM453386.1	China	Homo sapiens	2017	F
OM453388.1	China	Homo sapiens	2017	F
OM453394.1	China	Homo sapiens	2017	F
OM453405.1	China	Homo sapiens	2017	F
OM453410.1	China	Homo sapiens	2017	F
OM453414.1	China	Homo sapiens	2017	F
OM453415.1	China	Homo sapiens	2017	F
OM453421.1	China	Homo sapiens	2017	F
OM453422.1	China	Homo sapiens	2017	F
MT320802.1	China	Homo sapiens	2017	F
MT320805.1	China	Homo sapiens	2017	F
MT320808.1	China	Homo sapiens	2017	F
MT320811.1	China	Homo sapiens	2017	F
MK524356.1	China	Homo sapiens	2017	F
MK524362.1	China	Homo sapiens	2017	F
MK524368.1	China	Homo sapiens	2017	F
MK524374.1	China	Homo sapiens	2017	F
MK300945.1	China	Homo sapiens	2017	F

OP899816.1	China	Erinaceus amurensis	2018	F
OM453282.1	China	Homo sapiens	2018	F
OM453285.1	China	Homo sapiens	2018	F
OM453291.1	China	Homo sapiens	2018	F
OM453297.1	China	Homo sapiens	2018	F
OM453300.1	China	Homo sapiens	2018	F
OM453301.1	China	Homo sapiens	2018	F
OM453302.1	China	Homo sapiens	2018	F
OM453306.1	China	Homo sapiens	2018	F
OM453307.1	China	Homo sapiens	2018	F
OM453310.1	China	Homo sapiens	2018	F
OM453313.1	China	Homo sapiens	2018	F
OM453314.1	China	Homo sapiens	2018	F
OM453315.1	China	Homo sapiens	2018	F
OM453319.1	China	Homo sapiens	2018	F
OM453320.1	China	Homo sapiens	2018	F
OM453321.1	China	Homo sapiens	2018	F
OM453324.1	China	Homo sapiens	2018	F
OM453325.1	China	Homo sapiens	2018	F
OM453326.1	China	Homo sapiens	2018	F
OM453328.1	China	Homo sapiens	2018	F
OM453329.1	China	Homo sapiens	2018	F
OM453331.1	China	Homo sapiens	2018	F
OM453332.1	China	Homo sapiens	2018	F
OM453336.1	China	Homo sapiens	2018	F
OM453337.1	China	Homo sapiens	2018	F
OM453338.1	China	Homo sapiens	2018	F
OM453339.1	China	Homo sapiens	2018	F
OM453342.1	China	Homo sapiens	2018	F
OM453343.1	China	Homo sapiens	2018	F
OM453344.1	China	Homo sapiens	2018	F
OM453345.1	China	Homo sapiens	2018	F
OM453348.1	China	Homo sapiens	2018	F
OM453350.1	China	Homo sapiens	2018	F
OM453351.1	China	Homo sapiens	2018	F
OM453353.1	China	Homo sapiens	2018	F
OM453357.1	China	Homo sapiens	2018	F
OM453359.1	China	Homo sapiens	2018	F
OM453361.1	China	Homo sapiens	2018	F
OM453373.1	China	Homo sapiens	2018	F
OM453374.1	China	Homo sapiens	2018	F
OM453378.1	China	Homo sapiens	2018	F
OM453380.1	China	Homo sapiens	2018	F
OM453381.1	China	Homo sapiens	2018	F

OM453382.1	China	Homo sapiens	2018	F
OM453384.1	China	Homo sapiens	2018	F
OM453385.1	China	Homo sapiens	2018	F
OM453387.1	China	Homo sapiens	2018	F
OM453389.1	China	Homo sapiens	2018	F
OM453391.1	China	Homo sapiens	2018	F
OM453392.1	China	Homo sapiens	2018	F
OM453393.1	China	Homo sapiens	2018	F
OM453395.1	China	Homo sapiens	2018	F
OM453396.1	China	Homo sapiens	2018	F
OM453397.1	China	Homo sapiens	2018	F
OM453403.1	China	Homo sapiens	2018	F
OM453404.1	China	Homo sapiens	2018	F
OM453407.1	China	Homo sapiens	2018	F
OM453408.1	China	Homo sapiens	2018	F
OM453409.1	China	Homo sapiens	2018	F
OM453412.1	China	Homo sapiens	2018	F
OM453413.1	China	Homo sapiens	2018	F
OM453416.1	China	Homo sapiens	2018	F
OM453420.1	China	Homo sapiens	2018	F
OQ388741.1	China	Homo sapiens	2019	F
ON812183.1	China	Haemaphysalis longicornis	2019	F
OM453423.1	China	Homo sapiens	2019	F
OM453424.1	China	Homo sapiens	2019	F
OM453426.1	China	Homo sapiens	2019	F
OM453427.1	China	Homo sapiens	2019	F
OM453429.1	China	Homo sapiens	2019	F
OM453430.1	China	Homo sapiens	2019	F
OM453433.1	China	Homo sapiens	2019	F
OM453435.1	China	Homo sapiens	2019	F
OM453438.1	China	Homo sapiens	2019	F
OM453439.1	China	Homo sapiens	2019	F
OM453440.1	China	Homo sapiens	2019	F
OM453442.1	China	Homo sapiens	2019	F
OM453443.1	China	Homo sapiens	2019	F
OM453444.1	China	Homo sapiens	2019	F
OM453445.1	China	Homo sapiens	2019	F
OM453545.1	China	Homo sapiens	2019	F
OM453546.1	China	Homo sapiens	2019	F
OM453547.1	China	Homo sapiens	2019	F
OM453548.1	China	Homo sapiens	2019	F
OM453549.1	China	Homo sapiens	2019	F
OM453551.1	China	Homo sapiens	2019	F
OM453554.1	China	Homo sapiens	2019	F

OM453555.1	China	Homo sapiens	2019	F
OM453556.1	China	Homo sapiens	2019	F
OM453558.1	China	Homo sapiens	2019	F
OM453559.1	China	Homo sapiens	2019	F
OM453560.1	China	Homo sapiens	2019	F
OM453561.1	China	Homo sapiens	2019	F
OM453562.1	China	Homo sapiens	2019	F
OM453563.1	China	Homo sapiens	2019	F
OM453564.1	China	Homo sapiens	2019	F
OM453565.1	China	Homo sapiens	2019	F
OM453566.1	China	Homo sapiens	2019	F
OM453567.1	China	Homo sapiens	2019	F
OM453569.1	China	Homo sapiens	2019	F
OM453572.1	China	Homo sapiens	2019	F
OM453573.1	China	Homo sapiens	2019	F
OM453574.1	China	Homo sapiens	2019	F
OM453575.1	China	Homo sapiens	2019	F
OM453576.1	China	Homo sapiens	2019	F
OM453577.1	China	Homo sapiens	2019	F
OM453578.1	China	Homo sapiens	2019	F
OM453579.1	China	Homo sapiens	2019	F
OM453580.1	China	Homo sapiens	2019	F
OM453581.1	China	Homo sapiens	2019	F
OM453582.1	China	Homo sapiens	2019	F
OM453583.1	China	Homo sapiens	2019	F
OM453584.1	China	Homo sapiens	2019	F
OM453585.1	China	Homo sapiens	2019	F
OM453586.1	China	Homo sapiens	2019	F
OM453587.1	China	Homo sapiens	2019	F
OM453588.1	China	Homo sapiens	2019	F
OM453589.1	China	Homo sapiens	2019	F
OM453590.1	China	Homo sapiens	2019	F
OM453591.1	China	Homo sapiens	2019	F
OM453593.1	China	Homo sapiens	2019	F
OM453594.1	China	Homo sapiens	2019	F
OM453596.1	China	Homo sapiens	2019	F
OM453599.1	China	Homo sapiens	2019	F
OM453600.1	China	Homo sapiens	2019	F
OM453601.1	China	Homo sapiens	2019	F
OM453602.1	China	Homo sapiens	2019	F
OM453603.1	China	Homo sapiens	2019	F
OM453604.1	China	Homo sapiens	2019	F
OM453605.1	China	Homo sapiens	2019	F
OM453606.1	China	Homo sapiens	2019	F

OM453608.1	China	Homo sapiens	2019	F
OM453609.1	China	Homo sapiens	2019	F
OM453611.1	China	Homo sapiens	2019	F
OM453612.1	China	Homo sapiens	2019	F
OM453615.1	China	Homo sapiens	2019	F
OM453616.1	China	Homo sapiens	2019	F
OM453618.1	China	Homo sapiens	2019	F
OQ388784.1	China	Homo sapiens	2020	F
OM453446.1	China	Homo sapiens	2020	F
OM453450.1	China	Homo sapiens	2020	F
OM453452.1	China	Homo sapiens	2020	F
OM453453.1	China	Homo sapiens	2020	F
OM453454.1	China	Homo sapiens	2020	F
OM453455.1	China	Homo sapiens	2020	F
OM453456.1	China	Homo sapiens	2020	F
OM453457.1	China	Homo sapiens	2020	F
OM453459.1	China	Homo sapiens	2020	F
OM453460.1	China	Homo sapiens	2020	F
OM453461.1	China	Homo sapiens	2020	F
OM453463.1	China	Homo sapiens	2020	F
OM453464.1	China	Homo sapiens	2020	F
OM453465.1	China	Homo sapiens	2020	F
OM453469.1	China	Homo sapiens	2020	F
OM453473.1	China	Homo sapiens	2020	F
OM453474.1	China	Homo sapiens	2020	F
OM453476.1	China	Homo sapiens	2020	F
OM453478.1	China	Homo sapiens	2020	F
OM453479.1	China	Homo sapiens	2020	F
OM453482.1	China	Homo sapiens	2020	F
OM453483.1	China	Homo sapiens	2020	F
OM453484.1	China	Homo sapiens	2020	F
OM453485.1	China	Homo sapiens	2020	F
OM453486.1	China	Homo sapiens	2020	F
OM453487.1	China	Homo sapiens	2020	F
OM453488.1	China	Homo sapiens	2020	F
OM453489.1	China	Homo sapiens	2020	F
OM453490.1	China	Homo sapiens	2020	F
OM453492.1	China	Homo sapiens	2020	F
OM453493.1	China	Homo sapiens	2020	F
OM453495.1	China	Homo sapiens	2020	F
OM453497.1	China	Homo sapiens	2020	F
OM453499.1	China	Homo sapiens	2020	F
OM453500.1	China	Homo sapiens	2020	F
OM453502.1	China	Homo sapiens	2020	F

OM453504.1	China	Homo sapiens	2020	F
OM453505.1	China	Homo sapiens	2020	F
OM453506.1	China	Homo sapiens	2020	F
OM453509.1	China	Homo sapiens	2020	F
OM453510.1	China	Homo sapiens	2020	F
OM453511.1	China	Homo sapiens	2020	F
OM453512.1	China	Homo sapiens	2020	F
OM453517.1	China	Homo sapiens	2020	F
OM453518.1	China	Homo sapiens	2020	F
OM453519.1	China	Homo sapiens	2020	F
OM453520.1	China	Homo sapiens	2020	F
OM453521.1	China	Homo sapiens	2020	F
OM453522.1	China	Homo sapiens	2020	F
OM453523.1	China	Homo sapiens	2020	F
OM453524.1	China	Homo sapiens	2020	F
OM453525.1	China	Homo sapiens	2020	F
OM453526.1	China	Homo sapiens	2020	F
OM453527.1	China	Homo sapiens	2020	F
OM453528.1	China	Homo sapiens	2020	F
OM453530.1	China	Homo sapiens	2020	F
OM453531.1	China	Homo sapiens	2020	F
OM453532.1	China	Homo sapiens	2020	F
OM453534.1	China	Homo sapiens	2020	F
OM453535.1	China	Homo sapiens	2020	F
OM453537.1	China	Homo sapiens	2020	F
OM453538.1	China	Homo sapiens	2020	F
OM453539.1	China	Homo sapiens	2020	F
OM453540.1	China	Homo sapiens	2020	F
OM453541.1	China	Homo sapiens	2020	F
OQ388748.1	China	Homo sapiens	2021	F
OQ388753.1	China	Homo sapiens	2021	F
OQ388777.1	China	Homo sapiens	2021	F
OQ388779.1	China	Homo sapiens	2021	F
OQ388752.1	China	Homo sapiens	2022	F
OQ388761.1	China	Homo sapiens	2022	F
OQ388762.1	China	Homo sapiens	2022	F
OQ388763.1	China	Homo sapiens	2022	F
OQ388768.1	China	Homo sapiens	2022	F
OQ388770.1	China	Homo sapiens	2022	F
OQ388772.1	China	Homo sapiens	2022	F
OQ388773.1	China	Homo sapiens	2022	F
ON402247.1	China	Homo sapiens	2022	F
ON402248.1	China	Homo sapiens	2022	F
ON402249.1	China	Homo sapiens	2022	F

ON402250.1	China	Homo sapiens	2022	F
ON402251.1	China	Homo sapiens	2022	F
ON402252.1	China	Homo sapiens	2022	F

Note: 962 L fragments were classified, 911 human sequences, 45 animal sequences, 13 Blank Host.
