# Supplementary material for: Analysis of Gene Differences Between F and B Epidemic Lineages of Bandavirus Dabieense
Source: Microorganisms. 2025 Jan 28;13(2):292. doi: 10.3390/microorganisms13020292 (PMC11857831; doi:10.3390/microorganisms13020292)
Supplement: Supplementary file 1 [file microorganisms-13-00292-s001.zip › Supplementary Table 3.pdf]

Supplementary Table 3:

SFTSV Genome wide Gene Recombination Prediction Results

**Prediction results of S fragment gene recombination**	**Prediction results of M fragment gene recombination**	**Prediction results of L fragment gene recombination**
MK513931.1 (D)	OQ388971.1 (B)	MT320802.1 (F)
OM451720.1 (A)	OQ388970.1 (E)	OM452957.1 (D)
MT309105.1 (D)	OM452391.1 (A)	OM453477.1 (A)
	MG920820.1 (B)	MT005236.1 (D)
	OQ388989.1 (D)	AB983501.1 (B)
	OM452787.1 (B)	OM452994.1 (D)
	KR698339.1 (A)	OM453602.1 (F)
	OM452259.1 (D)	OM453119.1 (A)
	OM452332.1 (D)	OM453577.1 (F)
	OM452613.1 (D)	KR698352.1 (B)
	MT309104.1 (D)	MT005206.1 (D)
		OM453580.1 (F)
		OM453579.1 (F)
		OM453017.1 (D)
		OM453562.1 (F)
		OM453599.1 (F)
		OM452967.1 (F)
		OM453583.1 (F)

Note:The words in () represent lineages
