# Supplementary material for: Analysis of Gene Differences Between F and B Epidemic Lineages of Bandavirus Dabieense
Source: Microorganisms. 2025 Jan 28;13(2):292. doi: 10.3390/microorganisms13020292 (PMC11857831; doi:10.3390/microorganisms13020292)
Supplement: Supplementary file 1 [file microorganisms-13-00292-s001.zip › Supplementary Table 4.pdf]

Supplementary Table 4

Final SFTSV evolution rate and earliest ancestor prediction analysis statistical table

	**Evolution rate**	**The earliest ancestors predicted**	**Model data selection**
SF	2.43E-04	[1937.7509, 1998.1754]	GTR+F+G4+I+Uncorrelated relaxed clock+BayesianSkyline
SB	3.21E-04	[1869.404, 1983.3517]	
-	-		
MF	4.06E-05	[-824.7992, 1869.4691]	
MB	1.94E-04	[1729.975, 1958.5471]	
-	-		
LF	6.20E-05	[1204.63, 1927.8385]	
LB	7.44E-05	[-5428.4627, 1294.327]	

Note: SF represents the S fragment F lineage sequence of SFTSV

First SFTSV evolution rate and earliest ancestor prediction analysis statistical table

	**Evolution rate**	**The earliest ancestors predicted**	**Model data selection**
SF	9.18E-05	[-1576946717.9508, 1997.8654]	GTR+ALL+G4+Strictclock+BayesianSkyline
SB	2.20E-04	[716.4516, 1980.1685]	
-			
MF	9.48E-05	[-35237547093.1566, 1980.8137]	GTR+F+G4+Strictclock+BayesianSkyline
MB	1.46E-04	[-35312317.5234, 1998.4524]	
-			
LF	8.65E-05	[-27293343716627.06, 2008.6455]	GTR+F+G4+Strictclock+ConstantSize
LB	6.09E-05	[-67504735288563.086, 1855.2578]	
Incorporating geographical factors	-		
SF	6.18E-05	[-3824911017.8292, 1992.2937]	GTR+ALL+G4+Strictclock+BayesianSkyline
SB	2.74E-04	[-31787831.3504, 1891.9482]	
-			
MF	8.16E-05	[-49677803426.8066, 1998.6163]	GTR+F+G4+Strictclock+BayesianSkyline
MB	1.11E-04	[-7283474.0882, 1996.4333]	
-			
LF	6.60E-05	[-5799880785931215900, 2008.5802]	GTR+F+G4+Strictclock+ConstantSize
LB	7.84E-05	[-29877633013436364, 2011.493]	

Note: SF represents the S fragment F lineage sequence of SFTSV

Second SFTSV fragment evolution rate and earliest ancestor prediction analysis statistical table

Change in sequence length	Evolution rate	The earliest ancestors predicted	Model data selection
SF	5.68E-05	[237.4507, 1992.078]	GTR+ALL+GI4+Uncorrelated relaxed clock+ConstantSize
SB	0.273	[1974.7985, 2007.6728]	
-			
MF	4.35E-05	[-2070.6509, 1906.2468]	GTR+ALL+GI4+Uncorrelated relaxed clock+ConstantSize
MB	1.41E-04	[1480.4447, 1950.6274]	
-			
LF	3.64E-05	[-932.1633, 1906.4833]	GTR+ALL+GI4+Uncorrelated relaxed clock+ConstantSize
LB	3.68E-05	[-52658.5264, 1270.8168]	
-			
SFcds	2.81E-04	[1924.4338, 1997.2708]	GTR+ALL+GI4+Uncorrelated relaxed clock+ConstantSize
SBcds	4.37E-04	[1915.1346, 1988.5041]	
-			
MFcds	1.42E-06	[-113729.0425, -2589.9661]	GTR+ALL+GI4+Uncorrelated relaxed clock+ConstantSize
MBcds	1.78E-04	[1693.283, 1949.3146]	
-			
LFcds	4.23E-05	[534.6888, 1839.7362]	GTR+ALL+GI4+Uncorrelated relaxed clock+ConstantSize
LBcds	5.70E-05	[-10753.6015, 1154.4016]	

Note: SF represents the S fragment F lineage sequence of SFTSV
