# Supplementary material for: Analysis of Gene Differences Between F and B Epidemic Lineages of Bandavirus Dabieense
Source: Microorganisms. 2025 Jan 28;13(2):292. doi: 10.3390/microorganisms13020292 (PMC11857831; doi:10.3390/microorganisms13020292)
Supplement: Supplementary file 1 [file microorganisms-13-00292-s001.zip › Supplementary Table 5.pdf]

Supplementary Table 5: SFTSV Fragment Propagation Probability Data Table

fragment	from	TO	BAYES_FACTOR
SF	China_Liaoning	China_Hubei	1.038041601
SF	China_Liaoning	China	1.134340489
SF	China_Liaoning	China_Hebei	1.785289296
SF	China_Liaoning	China_Henan	1.028821701
SF	China_Liaoning	SouthKorea	1.789491268
SF	China_Liaoning	China_Jiangxi	1.202544364
SF	China_Hubei	China	1.253310802
SF	China_Hubei	China_Hebei	1.284879398
SF	China_Hubei	China_Henan	1.83391585
SF	China_Hubei	SouthKorea	1.351714941
SF	China_Hubei	China_Jiangxi	1.333268762
SF	China	China_Hebei	0.862586579
SF	China	China_Henan	29.13141011
SF	China	SouthKorea	0.771143004
SF	China	China_Jiangxi	0.940522587
SF	China_Hebei	China_Henan	0.482983454
SF	China_Hebei	SouthKorea	4.506744064
SF	China_Hebei	China_Jiangxi	11.34408831
SF	China_Henan	SouthKorea	1.415209616
SF	China_Henan	China_Jiangxi	1.400160473
SF	SouthKorea	China_Jiangxi	1.933206445
SF	China_Hubei	China_Liaoning	1.563604232
SF	China	China_Liaoning	1.714612899
SF	China_Hebei	China_Liaoning	5.622549443
SF	China_Henan	China_Liaoning	1.825411001
SF	SouthKorea	China_Liaoning	3.857633168
SF	China_Jiangxi	China_Liaoning	2.153108646
SF	China	China_Hubei	10.42110668
SF	China_Hebei	China_Hubei	2.035309892
SF	China_Henan	China_Hubei	1.739395364
SF	SouthKorea	China_Hubei	0.947038742
SF	China_Jiangxi	China_Hubei	1.564589349
SF	China_Hebei	China	534.2811676
SF	China_Henan	China	1.307615965
SF	SouthKorea	China	1.009630487
SF	China_Jiangxi	China	1.458856345
SF	China_Henan	China_Hebei	1.48377908

SF	SouthKorea	China_Hebei	7.69431851
SF	China_Jiangxi	China_Hebei	2.051101496
SF	SouthKorea	China_Henan	0.789677839
SF	China_Jiangxi	China_Henan	1.310354949
SF	China_Jiangxi	SouthKorea	1.693071448
SB	SouthKorea	China	1.186007661
SB	SouthKorea	China_Henan	1.320516008
SB	SouthKorea	Japan	1.348207301
SB	SouthKorea	China_Hubei	1.161438107
SB	SouthKorea	Thailand	1.111814355
SB	SouthKorea	China_Taiwan	0.999497504
SB	SouthKorea	China_Zhejiang	1.332798386
SB	China	China_Henan	2.277816292
SB	China	Japan	1.513311923
SB	China	China_Hubei	1.219683825
SB	China	Thailand	1.134111504
SB	China	China_Taiwan	1.14092432
SB	China	China_Zhejiang	1.935784167
SB	China_Henan	Japan	1.094457753
SB	China_Henan	China_Hubei	0.865166742
SB	China_Henan	Thailand	0.856147209
SB	China_Henan	China_Taiwan	0.710545773
SB	China_Henan	China_Zhejiang	11.55017282
SB	Japan	China_Hubei	1.353357479
SB	Japan	Thailand	1.282893624
SB	Japan	China_Taiwan	1.302166579
SB	Japan	China_Zhejiang	1.222669844
SB	China_Hubei	Thailand	2.682127774
SB	China_Hubei	China_Taiwan	1.425146853
SB	China_Hubei	China_Zhejiang	1.135083997
SB	Thailand	China_Taiwan	13.84152405
SB	Thailand	China_Zhejiang	0.753160905
SB	China_Taiwan	China_Zhejiang	1.424096785
SB	China	SouthKorea	2.539507084
SB	China_Henan	SouthKorea	4.117985273
SB	Japan	SouthKorea	1.564137391
SB	China_Hubei	SouthKorea	1.465262692
SB	Thailand	SouthKorea	1.051420807
SB	China_Taiwan	SouthKorea	3.731267228
SB	China_Zhejiang	SouthKorea	2.071729624
SB	China_Henan	China	32.91575249
SB	Japan	China	1.177143951
SB	China_Hubei	China	1.068575159
SB	Thailand	China	0.821187566

SB	China_Taiwan	China	3.875147542
SB	China_Zhejiang	China	1.447264612
SB	Japan	China_Henan	1.238635528
SB	China_Hubei	China_Henan	1.12537053
SB	Thailand	China_Henan	0.835488458
SB	China_Taiwan	China_Henan	21.60393676
SB	China_Zhejiang	China_Henan	2.2429792
SB	China_Hubei	Japan	2.356261106
SB	Thailand	Japan	5.145724577
SB	China_Taiwan	Japan	2.281704669
SB	China_Zhejiang	Japan	1.54242872
SB	Thailand	China_Hubei	18.88514416
SB	China_Taiwan	China_Hubei	2.864805012
SB	China_Zhejiang	China_Hubei	1.324605728
SB	China_Taiwan	Thailand	7.769795042
SB	China_Zhejiang	Thailand	1.390645071
SB	China_Zhejiang	China_Taiwan	1.465262692
MF	SouthKorea	China	1.780523823
MF	SouthKorea	China_Henan	35.59534891
MF	SouthKorea	China_Hubei	2.939230879
MF	SouthKorea	ChinaJiangxi	0.687260085
MF	SouthKorea	ChinaBeijing	32.26064729
MF	China	China_Henan	49.10510094
MF	China	China_Hubei	0.93003436
MF	China	ChinaJiangxi	6.052749068
MF	China	ChinaBeijing	0.504383599
MF	China_Henan	China_Hubei	1.070933173
MF	China_Henan	ChinaJiangxi	3.265743673
MF	China_Henan	ChinaBeijing	0.768486376
MF	China_Hubei	ChinaJiangxi	1.040677891
MF	China_Hubei	ChinaBeijing	1.050233846
MF	ChinaJiangxi	ChinaBeijing	1.396933836
MF	China	SouthKorea	376.2226626
MF	China_Henan	SouthKorea	0.761231791
MF	China_Hubei	SouthKorea	2.797389491
MF	ChinaJiangxi	SouthKorea	1.638225192
MF	ChinaBeijing	SouthKorea	1.545260642
MF	China_Henan	China	220.4656398
MF	China_Hubei	China	1.859646852
MF	ChinaJiangxi	China	1.819828322
MF	ChinaBeijing	China	1.841129812
MF	China_Hubei	China_Henan	1.278320885
MF	ChinaJiangxi	China_Henan	1.610205108
MF	ChinaBeijing	China_Henan	1.653704725

MF	ChinaJiangxi	China_Hubei	1.847938848
MF	ChinaBeijing	China_Hubei	2.335819295
MF	ChinaBeijing	ChinaJiangxi	1.295996758
MB	SouthKorea	China	4.714605145
MB	SouthKorea	China_Henan	4.855274845
MB	SouthKorea	Japan	0.587494453
MB	SouthKorea	China_Hubei	0.53086166
MB	SouthKorea	China_Taiwan	2.430369636
MB	SouthKorea	China_Zhejiang	2.012868188
MB	China	China_Henan	1.38331091
MB	China	Japan	1.008798737
MB	China	China_Hubei	0.87929311
MB	China	China_Taiwan	1.335108757
MB	China	China_Zhejiang	1.376781252
MB	China_Henan	Japan	0.563703899
MB	China_Henan	China_Hubei	0.492776071
MB	China_Henan	China_Taiwan	1.230055831
MB	China_Henan	China_Zhejiang	1.466505281
MB	Japan	China_Hubei	1.329591842
MB	Japan	China_Taiwan	1.199894309
MB	Japan	China_Zhejiang	1.148213683
MB	China_Hubei	China_Taiwan	1.203428198
MB	China_Hubei	China_Zhejiang	1.209621796
MB	China_Taiwan	China_Zhejiang	68.6827901
MB	China	SouthKorea	1.283067256
MB	China_Henan	SouthKorea	426.3699191
MB	Japan	SouthKorea	1.381443985
MB	China_Hubei	SouthKorea	1.567546402
MB	China_Taiwan	SouthKorea	1.13088155
MB	China_Zhejiang	SouthKorea	4.112222302
MB	China_Henan	China	7.581938172
MB	Japan	China	1.231838791
MB	China_Hubei	China	1.244346933
MB	China_Taiwan	China	0.620250304
MB	China_Zhejiang	China	1.62121402
MB	Japan	China_Henan	1.361904302
MB	China_Hubei	China_Henan	1.416152441
MB	China_Taiwan	China_Henan	1.001322879
MB	China_Zhejiang	China_Henan	4.143879524
MB	China_Hubei	Japan	2.157759982
MB	China_Taiwan	Japan	13.69454128
MB	China_Zhejiang	Japan	1.128289794
MB	China_Taiwan	China_Hubei	56.14918038
MB	China_Zhejiang	China_Hubei	0.61513093

MB	China_Zhejiang	China_Taiwan	6.791240035
LF	SouthKorea	China	0.444651579
LF	SouthKorea	China_Henan	0.876423806
LF	SouthKorea	China_Hubei	981.1076282
LF	SouthKorea	China_Hebei	10.75972381
LF	SouthKorea	ChinaAnhei	14.76453744
LF	China	China_Henan	1.167647175
LF	China	China_Hubei	1.197037123
LF	China	China_Hebei	1.131730718
LF	China	ChinaAnhei	1.054655897
LF	China_Henan	China_Hubei	65.34954261
LF	China_Henan	China_Hebei	0.512105435
LF	China_Henan	ChinaAnhei	0.503197838
LF	China_Hubei	China_Hebei	0.972589488
LF	China_Hubei	ChinaAnhei	0.942021336
LF	China_Hebei	ChinaAnhei	1.753972024
LF	China	SouthKorea	1.290360412
LF	China_Henan	SouthKorea	24.09191478
LF	China_Hubei	SouthKorea	3.993183218
LF	China_Hebei	SouthKorea	1.754916289
LF	ChinaAnhei	SouthKorea	1.71924112
LF	China_Henan	China	32.86065926
LF	China_Hubei	China	0.776424369
LF	China_Hebei	China	1.309732724
LF	ChinaAnhei	China	1.291164905
LF	China_Hubei	China_Henan	197.9921265
LF	China_Hebei	China_Henan	1.584232336
LF	ChinaAnhei	China_Henan	1.507672787
LF	China_Hebei	China_Hubei	1.537352999
LF	ChinaAnhei	China_Hubei	1.512888405
LF	ChinaAnhei	China_Hebei	2.118423706
LB	SouthKorea	China	0.468541487
LB	SouthKorea	China_Henan	2.185543478
LB	SouthKorea	Japan	0.808721363
LB	SouthKorea	China_Hubei	0.981730885
LB	SouthKorea	China_Taiwan	0.398385327
LB	SouthKorea	China_Zhejiang	10.30991374
LB	SouthKorea	China_Guizhou	4.206718257
LB	China	China_Henan	0.943686362
LB	China	Japan	1.054274301
LB	China	China_Hubei	1.606817784
LB	China	China_Taiwan	1.062848128
LB	China	China_Zhejiang	0.974276125
LB	China	China_Guizhou	1.026783231

LB	China_Henan	Japan	1.080056067
LB	China_Henan	China_Hubei	1.875336255
LB	China_Henan	China_Taiwan	0.857048137
LB	China_Henan	China_Zhejiang	1.325628845
LB	China_Henan	China_Guizhou	1.483344818
LB	Japan	China_Hubei	0.680421244
LB	Japan	China_Taiwan	0.466930662
LB	Japan	China_Zhejiang	1.339981458
LB	Japan	China_Guizhou	0.864263763
LB	China_Hubei	China_Taiwan	0.743549072
LB	China_Hubei	China_Zhejiang	1.282893624
LB	China_Hubei	China_Guizhou	1.763932239
LB	China_Taiwan	China_Zhejiang	1.188966916
LB	China_Taiwan	China_Guizhou	1.33484931
LB	China_Zhejiang	China_Guizhou	302.5684969
LB	China	SouthKorea	1.203798451
LB	China_Henan	SouthKorea	1.263718409
LB	Japan	SouthKorea	4703.648258
LB	China_Hubei	SouthKorea	1.862414912
LB	China_Taiwan	SouthKorea	1.570673511
LB	China_Zhejiang	SouthKorea	1.164377911
LB	China_Guizhou	SouthKorea	1.464201661
LB	China_Henan	China	0.928947646
LB	Japan	China	0.51151044
LB	China_Hubei	China	0.7856856
LB	China_Taiwan	China	2.29729358
LB	China_Zhejiang	China	20.98520809
LB	China_Guizhou	China	0.770704541
LB	Japan	China_Henan	26.27789669
LB	China_Hubei	China_Henan	0.806055726
LB	China_Taiwan	China_Henan	1.111814355
LB	China_Zhejiang	China_Henan	0.605814451
LB	China_Guizhou	China_Henan	1.988660052
LB	China_Hubei	Japan	1.610120139
LB	China_Taiwan	Japan	1.297085231
LB	China_Zhejiang	Japan	0.612530725
LB	China_Guizhou	Japan	72.99026083
LB	China_Taiwan	China_Hubei	1.773099514
LB	China_Zhejiang	China_Hubei	0.711410306
LB	China_Guizhou	China_Hubei	0.690720359
LB	China_Zhejiang	China_Taiwan	98.77493857
LB	China_Guizhou	China_Taiwan	0.458888058
LB	China_Guizhou	China_Zhejiang	1.902486582

Note: SF represents the full-length sequence of the F lineage S fragment of SFTSV, SFcds represents the coding region sequence analysis of the F lineage S fragment of DBV, and other abbreviations are the same as above.
