# Supplementary figures and images for: Analysis of Gene Differences Between F and B Epidemic Lineages of Bandavirus Dabieense
Source: Microorganisms. 2025 Jan 28;13(2):292. doi: 10.3390/microorganisms13020292 (PMC11857831; doi:10.3390/microorganisms13020292)

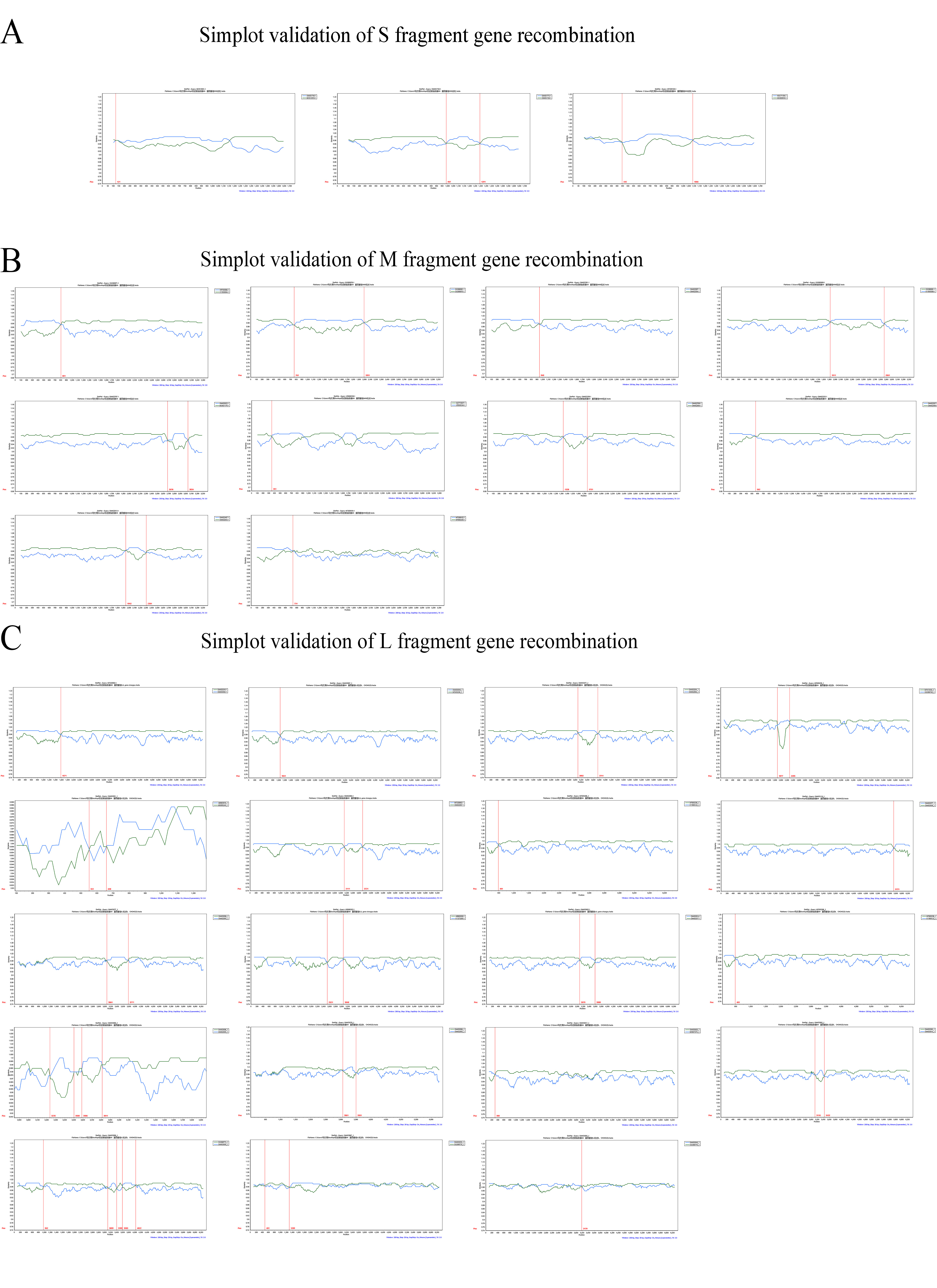

Supplement: Supplementary file 1 [file microorganisms-13-00292-s001.zip › Supplementary Figure 1.jpg]

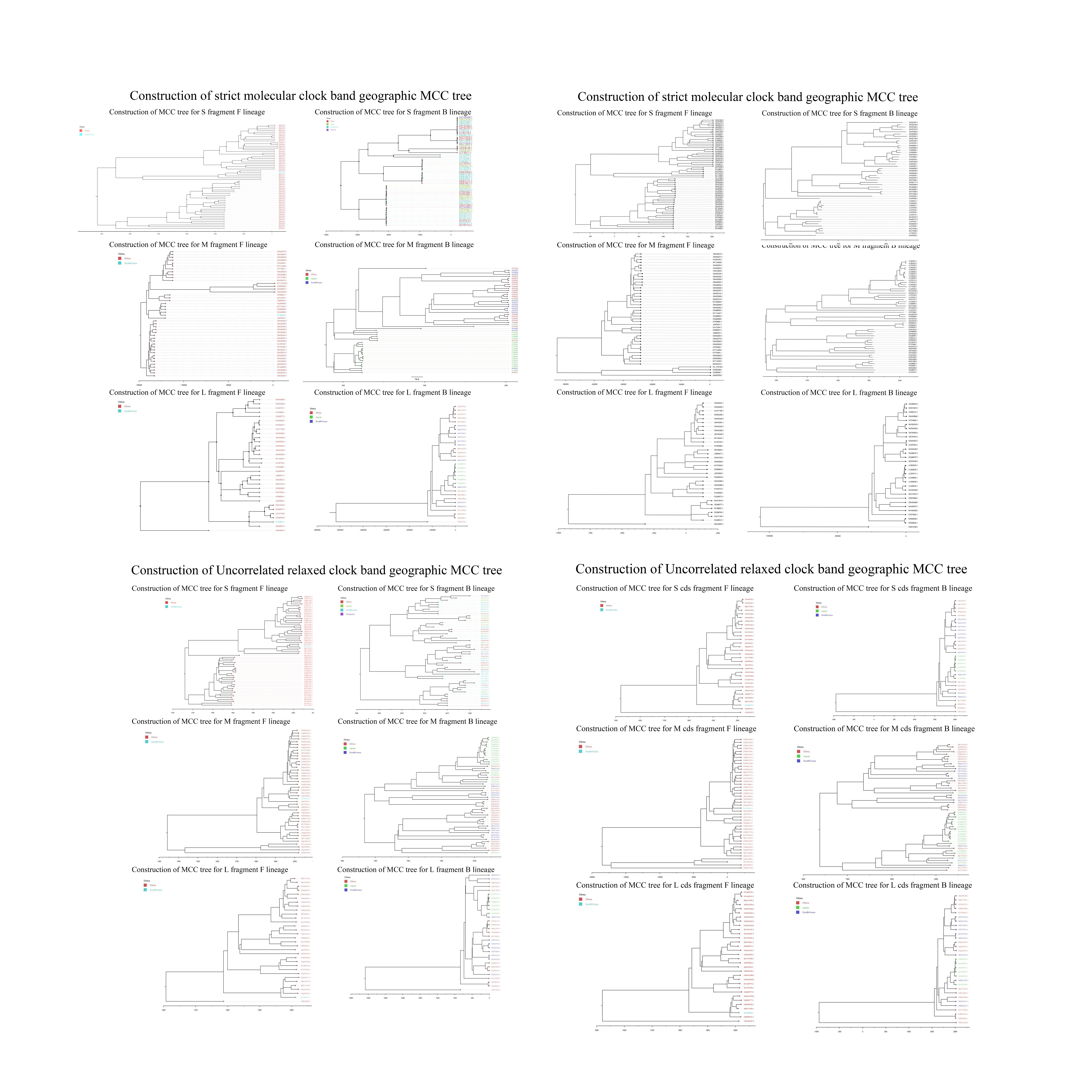

Supplement: Supplementary file 1 [file microorganisms-13-00292-s001.zip › Supplementary Figure 2.jpg]

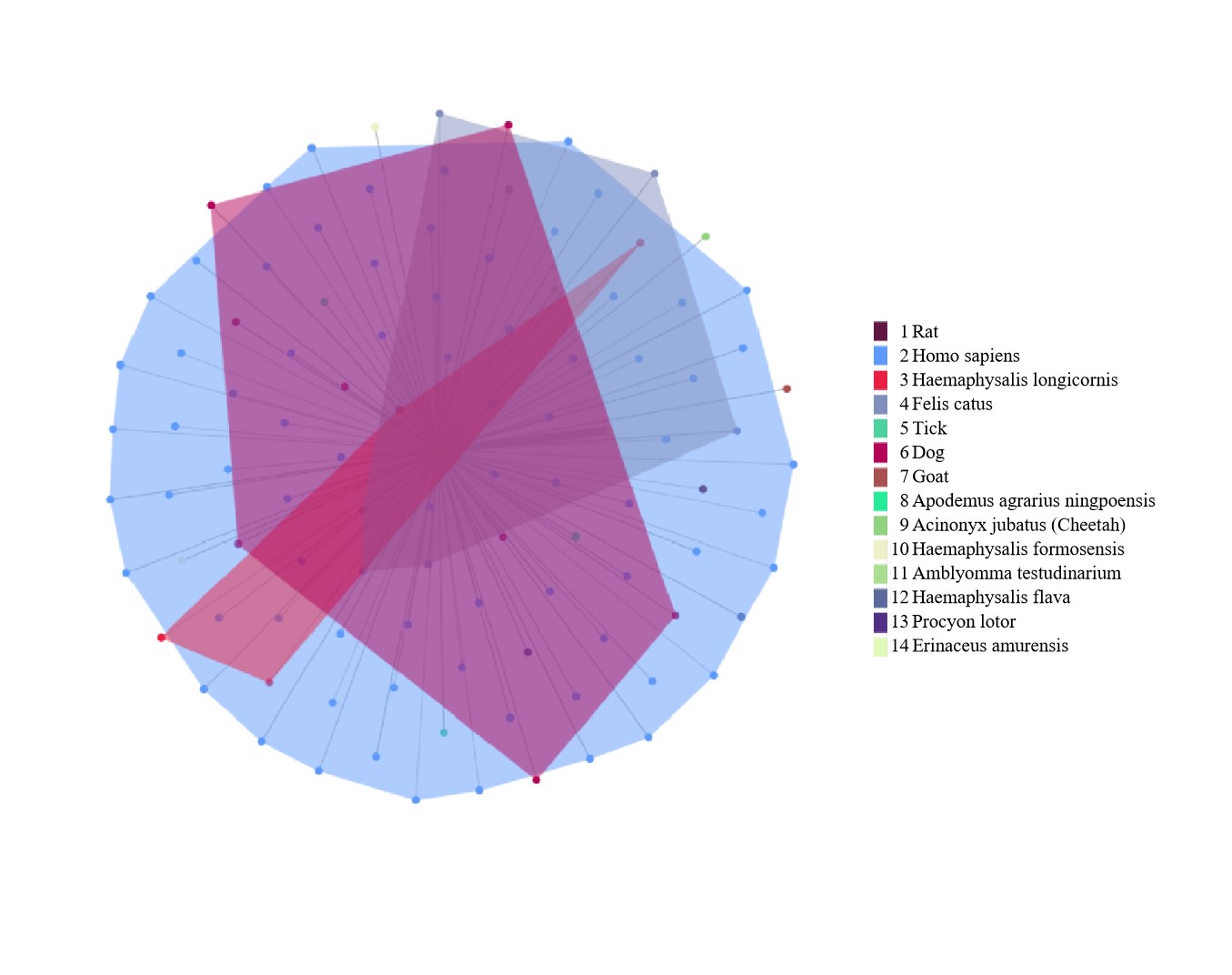

Supplement: Supplementary file 1 [file microorganisms-13-00292-s001.zip › Supplementary Figure 3.jpg]
